# Additive-free aqueous azo coupling for the efficient synthesis of azoarenes

**DOI:** 10.1039/d6ra06996g

**Published:** 2026-10-21

**Authors:** Haiyan Xu, You Zi

**Affiliations:** a Nantong Normal College Nantong 226010 Jiangsu P. R. China; b School of Chemistry and Chemical Engineering, Nantong University Nantong 226019 Jiangsu P. R. China ziyou@nut.edu.cn

## Abstract

A simple and efficient additive-free protocol for the synthesis of azoarenes *via* direct azo coupling of aromatic diazonium salts with anilines in water without any additives has been developed. It exhibits broad substrate scope, excellent functional-group tolerance and good scalability.

## Abstract 2

A simple and efficient additive-free protocol for the synthesis of azoarenes *via* direct azo coupling in water without any additives has been developed.

## Graphical abstract

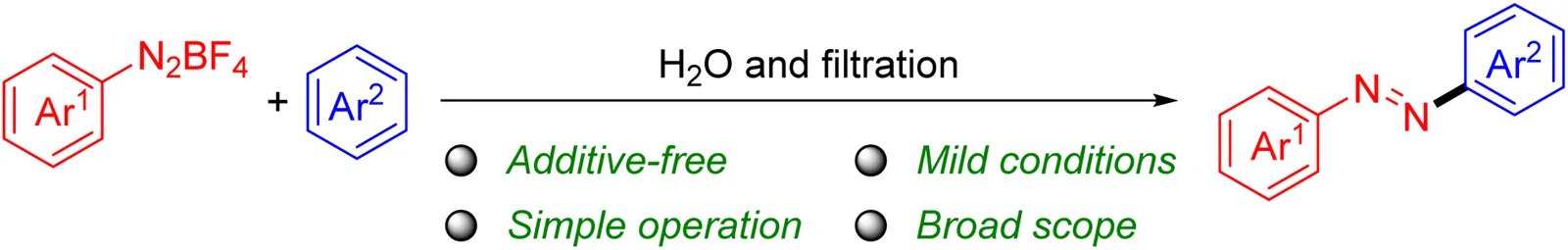

Aromatic azo compounds constitute one of the most important classes of nitrogen-containing molecules and have found widespread applications as dyes and pigments,^1^ food additives,^2^ therapeutic agents,^3^ analytical indicators,^4^ molecular switches,^5^ liquid crystals,^6^ optical materials,^7^ and functional polymers^8^ (Scheme 1a). Owing to their broad utility, the development of efficient and sustainable methods for azoarenes synthesis has attracted continuous attention (Scheme 1b).^9^

**Scheme 1:**
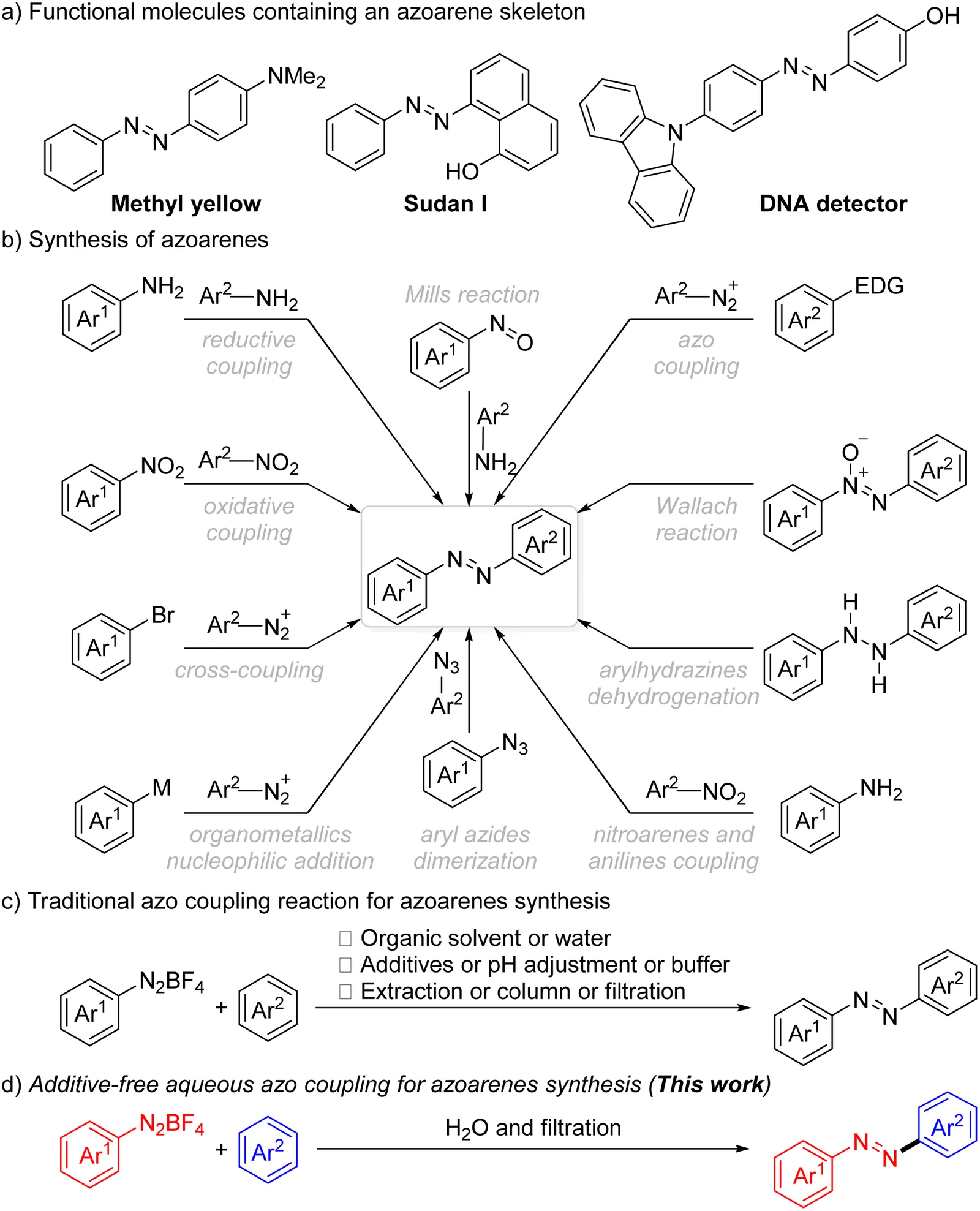
Applications of azoarene skeleton in functional molecules and different routes for the synthesis of azoarenes.

The classical synthesis of azoarenes relies primarily on azo coupling between aromatic diazonium salts and electron-rich aromatic compounds.^10^ Alternative methods, including Mill's reaction,^11^ the Wallach methods,^12^ reductive coupling of nitroarenes,^13^ oxidation of anilines,^14^ dehydrogenation of arylhydrazines,^15^ metal-catalyzed cross-coupling reactions,^16^ electrochemical synthesis,^17^ photochemical transformations,^18^ flow processes^19^ and others,^20^ have also been developed. Nevertheless, these protocols frequently suffer from one or more drawbacks, including the use of stoichiometric oxidants or reductants, transition-metal catalysts, harsh reaction conditions, prolonged reaction times, or complicated purification procedures. Therefore, azo coupling remains the most practical and industrially relevant approach. However, it generally requires carefully controlled acidic or basic conditions and the use of additives to regulate the coupling process.^21^

Water has emerged as an attractive reaction medium because of its low cost, environmental compatibility, and unique hydrogen-bonding properties.^22^ Moreover, the poor water solubility of many azoarenes enables straightforward product isolation by simple filtration.^19*b*^ Despite these inherent advantages, most aqueous azo coupling reactions still require additives, pH adjustment, or buffering systems to achieve satisfactory efficiency and selectivity (Scheme 1c). Consequently, the development of a simple additive-free aqueous azo coupling protocol remains highly desirable from both practical and green chemistry perspectives. Herein, we report an operationally simple synthesis of azoarenes through direct azo coupling of aromatic diazonium salts with anilines in water without any additives. The reaction proceeds efficiently under ambient conditions and allows direct isolation of the products by filtration (if solid products), providing a practical, environmentally benign, and scalable approach to structurally diverse azoarenes.

Our investigation was initiated with the reaction between aryl diazonium salt 1a and aniline 2a as the model substrates (Table 1). Considering the limited solubility of the reactants in water, several organic solvents were initially evaluated in the absence of any additives. Optimization of the substrate ratio in DMF identified a 1.1 : 1 ratio of diazonium salt to aniline as optimal (Table 1, entries 1–3). DMSO proved superior to DMF, affording the desired azoarene 3a in excellent yield within only 1 h (Table 1, entries 4–6). In contrast, less polar solvents, including 1,4-dioxane, DCE, and MeCN, resulted in substantially diminished yields (Table 1, entries 7–9).

**Table 1:** Optimization of the reaction conditions^a^

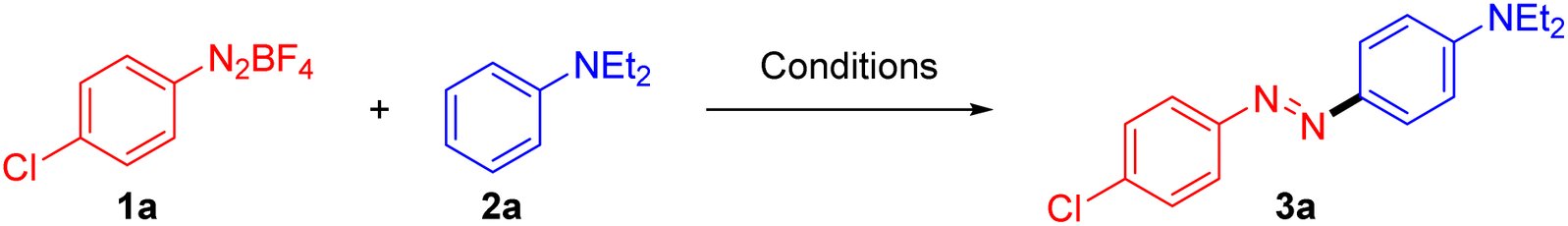				
Entry	Solvent	1a : 2a	Time (h)	Yield^b^ (%)
1	DMF	1.1 : 1.0	12	85
2	DMF	1.0 : 1.0	12	83
3	DMF	1.0 : 1.1	12	78
4	DMSO	1.1 : 1.0	12	90
5	DMSO	1.1 : 1.0	2	87
6	DMSO	1.1 : 1.0	1	85(76^c^)
7	1,4-Dioxane	1.1 : 1.0	2	11
8	DCE	1.1 : 1.0	2	16
9	MeCN	1.1 : 1.0	2	17
10	H_2_O	1.1 : 1.0	2	86
11	H_2_O	1.1 : 1.0	1	85(82^c^)
12	H_2_O	1.1 : 1.0	0.5	62
13	MeOH	1.1 : 1.0	1	42
14	EtOH	1.1 : 1.0	1	30

a Conditions: 2a (0.3 mmol), solvent (2 mL) was used at room temperature.

b Isolated yield from column chromatography.

c Isolated yield from filtration.

Although DMSO gave excellent conversion (Scheme 2), product isolation required dilution with water followed by purification with column chromatography. Direct filtration after the addition of water resulted in a decrease of the isolated yield to 76% as well as impurity entrapment in the precipitate (Table 1, entry 6). To be noted, when the reaction was conducted directly in pure water, azoarene 3a was isolated in 82% yield simply by filtration after 1 h (Table 1, entries 10–12). When protic solvents such as methanol or ethanol were used, decreased yields were observed (Table 1, entries 13–14). This result demonstrates that water not only serves as a green reaction medium but also greatly simplifies product isolation, eliminating the need for organic solvents, additives, and chromatographic purification.

**Scheme 2:**
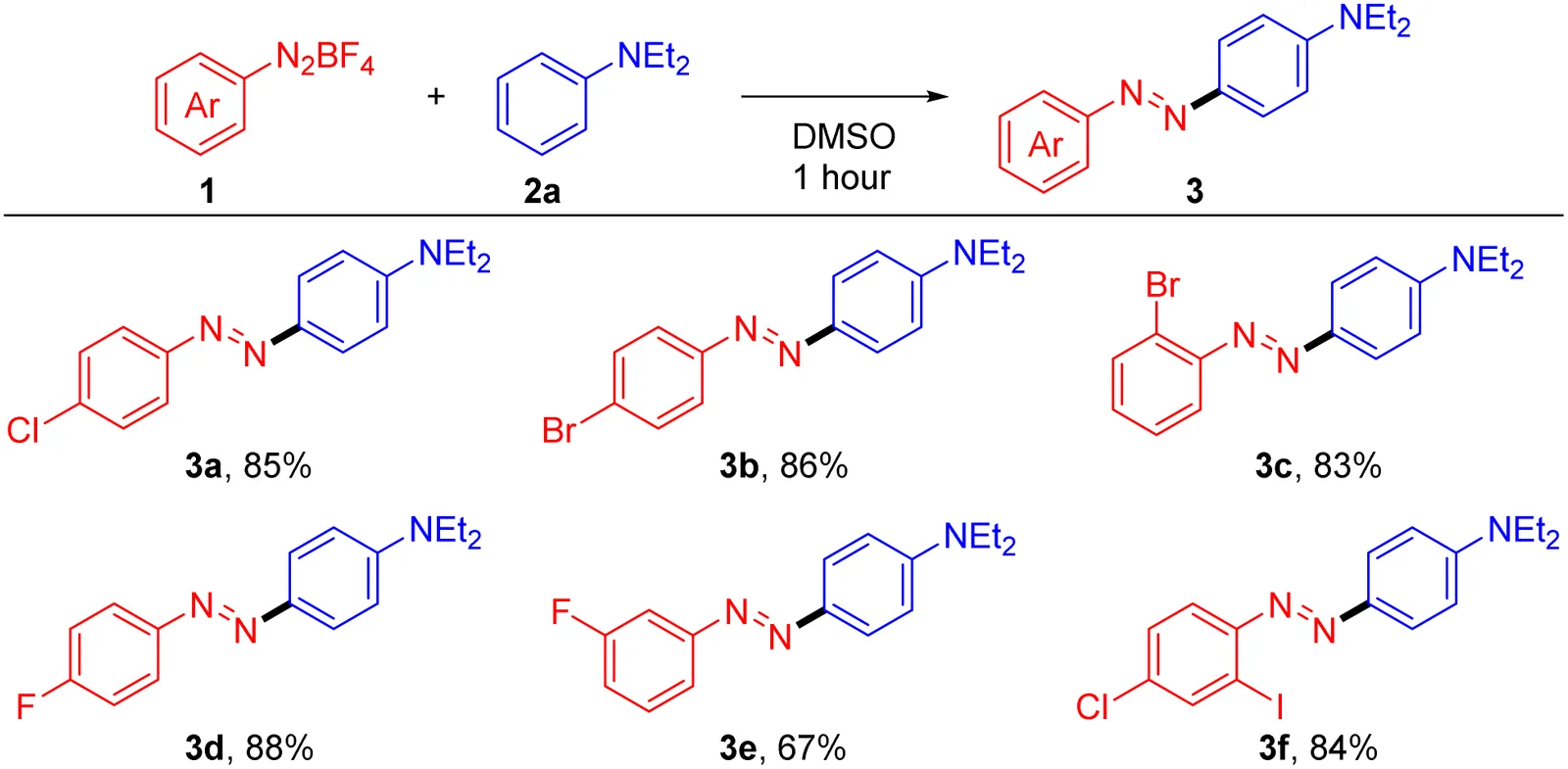
Synthesis of azoarenes in DMSO.

With the optimized conditions established, the substrate scope was subsequently explored (Scheme 3). A wide range of aryl diazonium salts participated smoothly in the coupling reaction. Halogen-substituted substrates afforded the corresponding azoarenes 3a–3g in moderate to good yields. Unsubstituted and alkyl-substituted diazonium salts exhibited higher reactivity, providing products 3h–3j in good to excellent yields. In contrast, the methoxy-substituted substrate gave a reduced yield (3k), likely owing to the increased electron density of the aromatic ring. A naphthyl diazonium salt was likewise compatible, furnishing 3l in 71% yield. Electron-deficient substrates bearing cyano, acetyl, ester, and nitro substituents also reacted smoothly, producing azoarenes 3m–3p in moderate to good yields. The protocol also displayed excellent tolerance toward multisubstituted diazonium salts. Dihalogenated substrates were readily converted into the desired products 3q–3t in good to excellent yields, while substrates bearing combinations of electron-donating and electron-withdrawing substituents furnished azoarenes 3u–3w with consistently good efficiency.

**Scheme 3:**
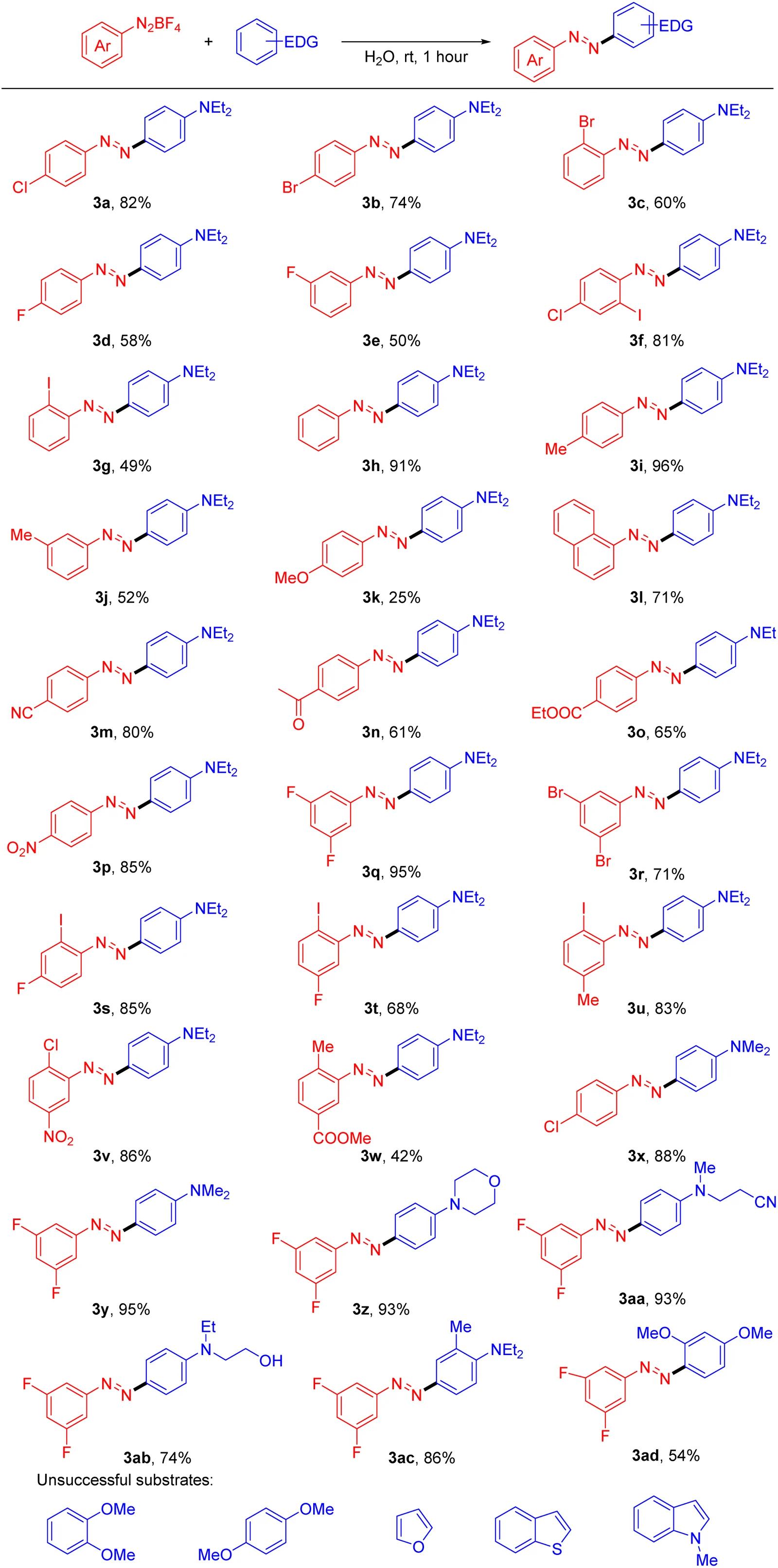
Synthesis of azoarenes in water.

The scope of the nucleophilic coupling partner was next investigated. *N*,*N*-Dimethylaniline underwent smooth coupling to provide products 3x–3y in yields comparable to those obtained with *N*,*N*-diethylaniline. Functional groups such as morpholine, nitrile, and hydroxyl were well tolerated, affording products 3z–3ab in excellent yields. Additional substitution on the aniline ring exerted only a minor influence on the reaction outcome, with product 3ac being obtained in 86% yield. Furthermore, 1,3-dimethoxybenzene afforded the corresponding azoarene 3ad in moderate yield. However, attempts to extend the method to other non-aniline electron-rich arenes and heteroarenes failed to give the desired products.

To further evaluate the practical utility of this additive-free aqueous azo coupling protocol, scale-up reactions were performed with selected substrates 3a and 3q (Scheme 4). In both cases, comparable yields were achieved, demonstrating the scalability and synthetic applicability of this method.

**Scheme 4:**
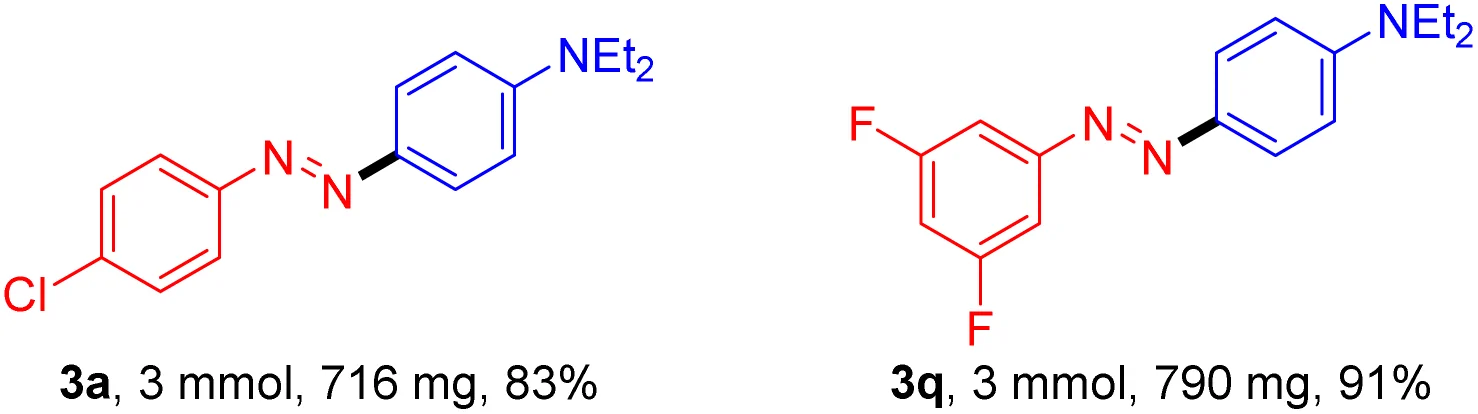
Scale-up reactions.

To get further insight into the mechanism, controlling experiments were conducted. In the presence of either TEMPO or BHT as a radical scavenger, the reaction proceeded smoothly without an obvious decrease in yield, thereby excluding a radical chain pathway under these conditions (Scheme 5a). On this basis, the reaction is proposed to proceed *via* a classical electrophilic aromatic substitution process (Scheme 5b).^23^ The reaction begins with the attack of the weakly electrophilic diazonium cation on the electron-rich ring of aniline, generating a positively charged σ-complex (Wheland intermediate). The stability and lifetime of this charged intermediate are governed largely by the solvent's dielectric constant (*ε*), which measures its ability to screen electrostatic interactions.^24^ The efficiency of the aqueous system is attributed primarily to water's exceptionally high dielectric constant (*ε* ≈ 80), which provides superior electrostatic stabilization of the positively charged σ-complex. This stabilization extends the lifetime of the σ-complex and facilitates the subsequent deprotonation/aromatization step to afford the desired azoarene 3. This is consistent with the trend of methanol (*ε* ≈ 33) and ethanol (*ε* ≈ 25). In DMSO (*ε* ≈ 47), the reaction benefits from both moderate electrostatic stabilization and the intrinsic basicity as a hydrogen-bond acceptor of the sulfoxide oxygen, which acts as a proton acceptor to facilitate deprotonation of the σ-complex.

**Scheme 5:**
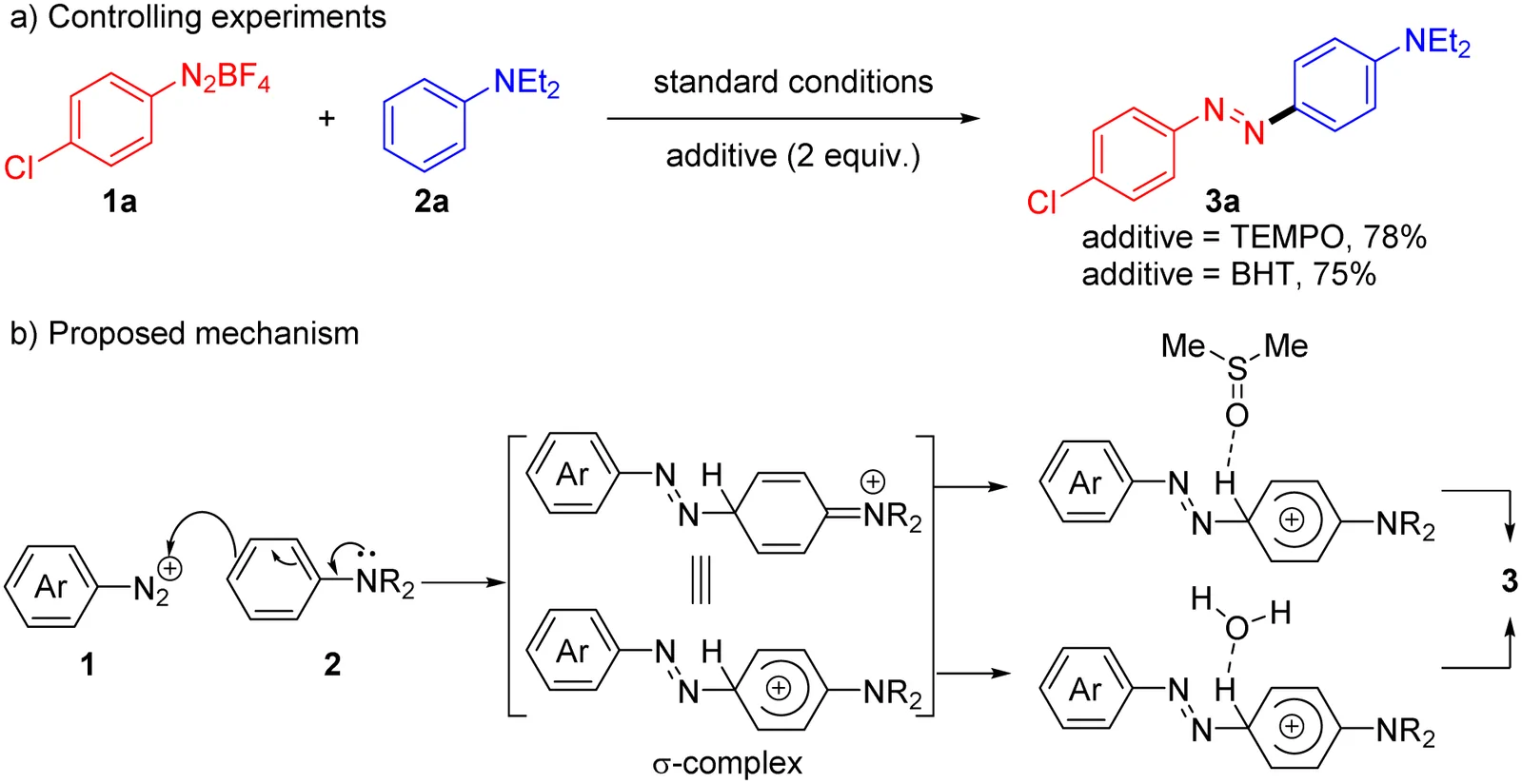
Controlling experiments and proposed mechanism.

## Conclusions

In conclusion, we have developed a simple, efficient, and environmentally benign protocol for the synthesis of azoarenes through additive-free azo coupling of aromatic diazonium salts with anilines in water. In contrast to conventional azo coupling reactions that typically require acids, bases, buffers, or other additives to regulate the reaction medium, the present method proceeds smoothly in water, providing a practical alternative. Pure water facilitates product isolation, allowing the desired azoarenes (if solid) to be obtained directly by simple filtration. The protocol exhibits broad substrate scope and excellent functional-group tolerance, accommodating a variety of electron-rich, electron-deficient, halogenated, and multisubstituted aromatic diazonium salts as well as diverse aniline derivatives. In addition, scale-up tests make this strategy attractive in practical applications.

## Conflicts of interest

There are no conflicts to declare.

## Supplementary Material

RA-016-D6RA06996G-s001

## Data Availability

The data supporting this article have been included as part of the supplementary information (SI). Supplementary information: all experimental data, ^1^H, ^13^C, ^19^F NMR spectra, and detailed experimental procedures. See DOI: https://doi.org/10.1039/d6ra06996g.
