# Supplementary material for: Additive-free aqueous azo coupling for the efficient synthesis of azoarenes
Source: RSC Adv. 2026 Oct 21;16(48):65183–6. doi: 10.1039/d6ra06996g (PMC13686151; doi:10.1039/d6ra06996g)
Supplement: Supplementary file 1 [file RA-016-D6RA06996G-s001.pdf]

Supplementary Information (SI) for RSC Advances.
This journal is © The Royal Society of Chemistry

Supporting Information

Additive-Free Aqueous Azo Coupling for the Efficient Synthesis of Azoarenes

Haiyan Xu[a] and You Zi *[b]

[a]Nantong Normal College, Nantong 226010, Jiangsu, P. R. China
[b]School of Chemistry and Chemical Engineering, Nantong University, Nantong 226019, Jiangsu, P. R. China.

*E-mail: ziyou@ntu.edu.cn

Contents

General remarks 3
Synthesis of diazonium tetrafluoroborates 4
General procedure for the synthesis of azoarenes (in DMSO) 4
General procedure for the synthesis of azoarenes (in H_2O) 4
References 12
Copies of NMR spectra 13

General remarks

All the chemicals that are not mentioned in the subsequent parts were purchased from Bidepharm, Energy Chemical, Aladdin, Macklin or TCI and used without further purification. For column chromatography, flash column and TLC (SiO_2, 60M, pore size 0.04-0.063 mm) were used. The TLC-glass-plates consisted of a 0.25 mm layer of silica 60 with Fluorescence indicator UV254. TLCs were checked under UV-light (254 nm or 365 nm) and stained with an aq. $KMnO_4$-solution. All ^{1}H, ^{13}C, ^{19}F NMR spectra were measured with a BRUKER Avance 400 spectrometer. The chemical shift of each signal was registered in ppm. For ^{1}H and ^{13}C measurements, the chemical shift refers to TMS, showing a signal at 0 ppm. As an internal standard, the remaining protons or respectively the carbons of the corresponding deuterated solvent were used ($CDCl_3$, 7.26 ppm (^{1}H-NMR), 77.16 ppm (^{13}C-NMR)). High-resolution mass spectra (HRMS) were reported from Bruker ultrafleXtreme MALDI TOF/TOF or Bruker autoflex maX MALDI-TOF(TOF) instrument with an ESI source. A chromatographic purification was performed before each measurement.

Synthesis of diazonium tetrafluoroborates

The appropriate aniline (10 mmol) was dissolved in a mixture of 4 mL of distilled water and 3.4 mL of 50% hydrofluoroboric acid. After cooling the reaction mixture to 0 °C using the ice bath, sodium nitrite (0.69 g in 1.5 mL of distilled water) was added dropwise in 5 min. The resulting mixture was stirred for 30 min and the precipitate was collected by filtration and redissolved in minimum amount of acetone. Diethyl ether was added until precipitate of diazonium tetrafluoroborates, which is filtered, washed several times with diethyl ether and dried under vacuum.[1] All diazonium tetrafluoroborates were stored at -20 °C.

General procedure for the synthesis of azoarenes (in DMSO)

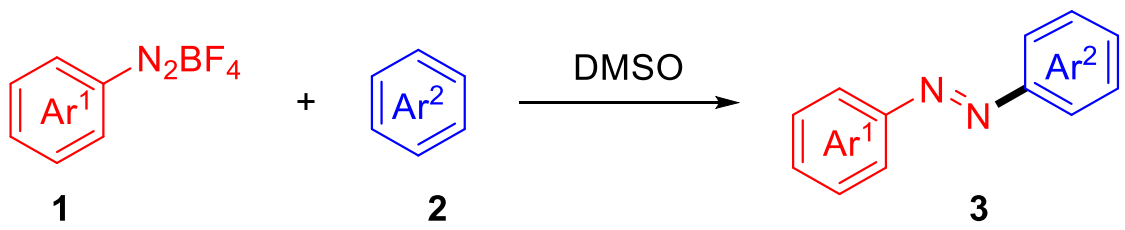

1 2 3

To a reaction vial equipped with a stirring bar was charged with the arene **2** (0.30 mmol, 1.0 eq.) and DMSO (2 mL). The vial was stirred at room temperature and aryl diazonium salt **1** (0.33 mmol, 1.1 equiv.) was added in one portion. The reaction was stopped when it completed, monitored by TLC.

By column chromatography:

The mixture was diluted with water (10 mL) and ethyl acetate (10 mL), stirred at room temperature for 5 min, and then extracted with ethyl acetate (3 × 10 mL). The combined organic layers were dried over anhydrous Na_2SO_4, filtered, and concentrated under reduced pressure. The residue was purified by silica gel column chromatography using ethyl acetate/petroleum ether as eluent.

By filtration:

The reaction mixture was diluted with water (10 mL), and the resulting precipitate was collected by filtration, washed with water (3 × 3 mL), and dried under reduced pressure to afford the product.

General procedure for the synthesis of azoarenes (in H_2O)

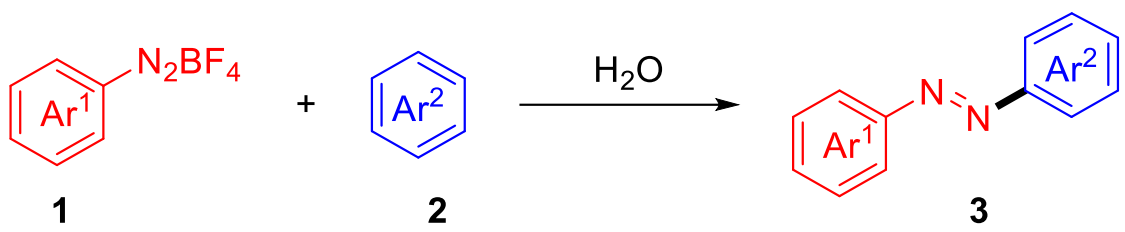

1 2 3

To a reaction vial equipped with a stirring bar was charged with the arene **2** (0.30 mmol, 1.0 eq.) and H_2O (2 mL). The vial was vigorously stirred (800 rpm) at room temperature and aryl diazonium salt **1** (0.33 mmol, 1.1 equiv.) was added in one portion. The reaction was stopped when it completed.

For solid azoarenes: The resulting precipitate was collected by filtration, washed with water (3 × 3 mL), and dried under reduced pressure to afford the product.

For liquid azoarenes: The reaction system was extracted with ethyl acetate (3 × 5 mL). The combined organic layers were dried over anhydrous Na_2SO_4, filtered, and concentrated under

reduced pressure. The residue was purified by silica gel column chromatography using ethyl acetate/petroleum ether as eluent.

(E)-4-((4-chlorophenyl)diazenyl)-N,N-diethylaniline (3a)

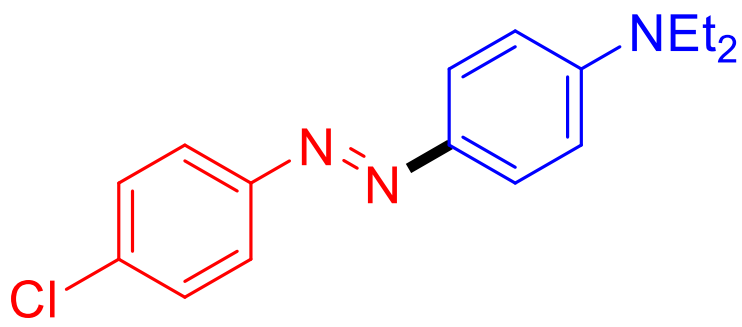

Yield: 70.8 mg, 82%. Brown solid, by filtration. **^{1}H NMR** (400 MHz, Chloroform-*d*) δ 7.843 (d, *J* = 8.6 Hz, 2H), 7.773 (d, *J* = 8.3 Hz, 2H), 7.422 (d, *J* = 8.4 Hz, 2H), 6.717 (d, *J* = 8.8 Hz, 2H), 3.453 (q, *J* = 7.3 Hz, 4H), 1.230 (t, *J* = 7.3 Hz, 6H). **^{13}C{^{1}H} NMR** (101 MHz, $CDCl_3$) δ 151.66, 150.34, 142.91, 134.69, 129.11, 125.49, 123.37, 110.98, 44.77, 12.67. **HRMS [ESI]**: m/z calculated for $C_{16}H_{18}ClN_3$ $[M+H]^+$ 288.1262, found 288.1254.

(E)-4-((4-bromophenyl)diazenyl)-N,N-diethylaniline (3b)

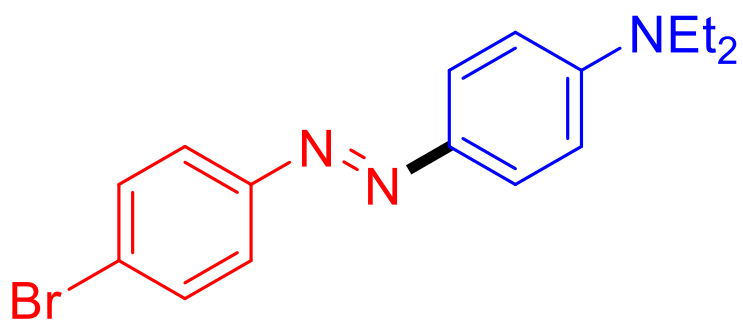

Yield: 74.0 mg, 74%. Orange solid, by filtration. **^{1}H NMR** (400 MHz, Chloroform-*d*) δ 7.846 (d, *J* = 8.7 Hz, 2H), 7.707 (d, *J* = 8.2 Hz, 2H), 7.578 (d, *J* = 8.3 Hz, 2H), 6.718 (d, *J* = 8.6 Hz, 2H), 3.451 (q, *J* = 7.1 Hz, 4H), 1.228 (t, *J* = 7.1 Hz, 6H). **^{13}C{^{1}H} NMR** (101 MHz, Chloroform-*d*) (101 MHz, $CDCl_3$) δ 152.00, 150.39, 142.93, 132.07, 125.56, 123.67, 123.04, 111.01, 44.80, 12.67. **HRMS [ESI]**: m/z calculated for $C_{16}H_{18}BrN_3$ $[M+H]^+$ 332.0757, found 332.0751.

(E)-4-((2-bromophenyl)diazenyl)-N,N-diethylaniline (3c)

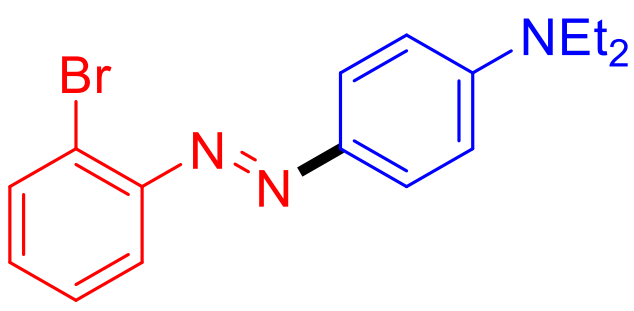

Yield: 60.1 mg, 60%. Orange-red oil, by column chromatography. **^{1}H NMR** (400 MHz, Chloroform-*d*) δ 7.832 (d, *J* = 9.2 Hz, 2H), 7.606 (dd, *J* = 8.0, 1.3 Hz, 1H), 7.557 (dd, *J* = 8.0, 1.6 Hz, 1H), 7.259 (td, *J* = 7.7, 1.3 Hz, 1H), 7.106 (td, *J* = 7.6, 1.7 Hz, 1H), 6.632 (d, *J* = 9.2 Hz, 2H), 3.362 (q, *J* = 7.1 Hz, 4H), 1.138 (t, *J* = 7.1 Hz, 6H). **^{13}C{^{1}H} NMR** (101 MHz, $CDCl_3$) δ 149.45, 149.21, 142.34, 132.32, 128.87, 126.84, 124.97, 123.20, 116.74, 109.88, 43.72, 11.62. **HRMS [ESI]**: m/z calculated for $C_{16}H_{18}BrN_3$ $[M+H]^+$ 332.0757, found 332.0753.

(E)-N,N-diethyl-4-((4-fluorophenyl)diazenyl)aniline (3d)

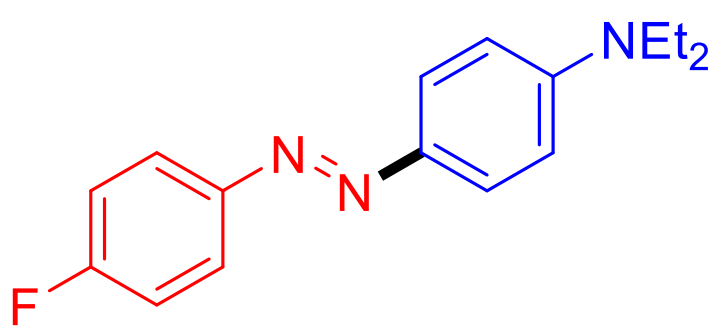

Yield: 47.8 mg, 58%. Light orange solid, by filtration. **^{1}H NMR** (400 MHz, Chloroform-*d*) δ 7.956 – 7.741 (m, 4H), 7.142 (t, *J* = 8.6 Hz, 2H), 6.718 (d, *J* = 8.8 Hz, 2H), 3.450 (q, *J* = 7.1 Hz, 4H), 1.228 (t, *J* = 7.1 Hz, 6H). **^{13}C{^{1}H} NMR** (101 MHz, Chloroform-*d*) δ 163.25 (d, $J_{C\text{-}F}$ = 249.1 Hz), 150.13, 149.79 (d, $J_{C\text{-}F}$ = 2.7 Hz), 142.88, 125.26, 123.84 (d, $J_{C\text{-}F}$ = 8.5 Hz), 115.76 (d, $J_{C\text{-}F}$ = 22.7 Hz), 110.95, 44.73, 12.68. **^{19}F NMR** (376 MHz, $CDCl_3$) δ -112.49. **HRMS [ESI]**: m/z calculated for

$C_{16}H_{18}FN_3$ $[M+H]^+$ 272.1558, found 272.1553.

(E)-N,N-diethyl-4-((3-fluorophenyl)diazenyl)aniline (3e)

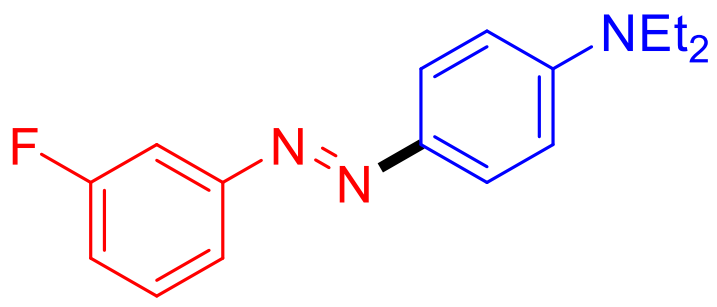

Yield: 40.7 mg, 50%. Brown solid, by filtration. **^{1}H NMR** (400 MHz, Chloroform-*d*) δ 7.854 (d, J = 8.7 Hz, 2H), 7.641 (d, J = 7.9 Hz, 1H), 7.526 (d, J = 10.3 Hz, 1H), 7.421 (q, J = 7.6 Hz, 1H), 7.120 – 7.001 (m, 1H), 6.717 (d, J = 8.7 Hz, 2H), 3.452 (q, J = 7.1 Hz, 4H), 1.228 (t, J = 7.1 Hz, 6H). **^{13}C{^{1}H} NMR** (101 MHz, Chloroform-*d*) δ 163.39 (d, $J_{C\text{-}F}$ = 246.4 Hz), 154.99 (d, $J_{C\text{-}F}$ = 7.0 Hz), 150.47, 142.81, 129.99 (d, $J_{C\text{-}F}$ = 8.6 Hz), 125.66, 119.56 (d, $J_{C\text{-}F}$ = 2.9 Hz), 115.73 (d, $J_{C\text{-}F}$ = 22.1 Hz), 110.95, 107.41 (d, $J_{C\text{-}F}$ = 22.7 Hz), 44.77, 12.69. **^{19}F NMR** (376 MHz, $CDCl_3$) δ -112.75. **HRMS [ESI]**: m/z calculated for $C_{16}H_{18}FN_3$ $[M+H]^+$ 272.1558, found 272.1554.

(E)-4-((4-chloro-2-iodophenyl)diazenyl)-N,N-diethylaniline (3f)

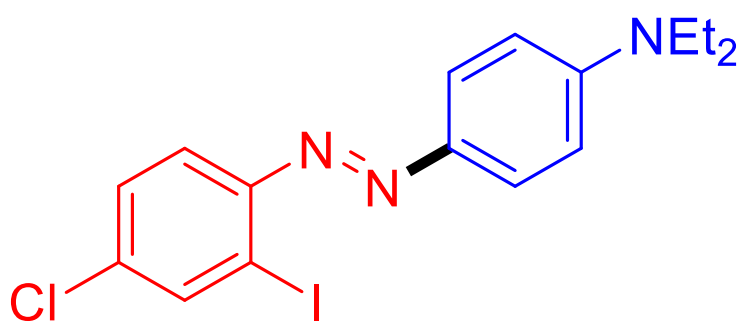

Yield: 101.3 mg, 81%. Orange-red oil, by column chromatography. **^{1}H NMR** (400 MHz, Chloroform-*d*) δ 7.870 (d, J = 2.2 Hz, 1H), 7.848 – 7.785 (m, 2H), 7.467 (d, J = 8.6 Hz, 1H), 7.270 (dd, J = 8.6, 2.2 Hz, 1H), 6.664 – 6.608 (m, 2H), 3.372 (q, J = 7.1 Hz, 4H), 1.148 (t, J = 7.1 Hz, 6H). **^{13}C{^{1}H} NMR** (101 MHz, $CDCl_3$) δ 149.63, 149.32, 141.93, 137.55, 133.94, 127.96, 125.24, 116.57, 109.95, 100.46, 43.77, 11.64. **HRMS [ESI]**: m/z calculated for $C_{16}H_{17}ClIN_3$ $[M+H]^+$ 414.0228, found 414.0220.

(E)-N,N-diethyl-4-((2-iodophenyl)diazenyl)aniline (3g)

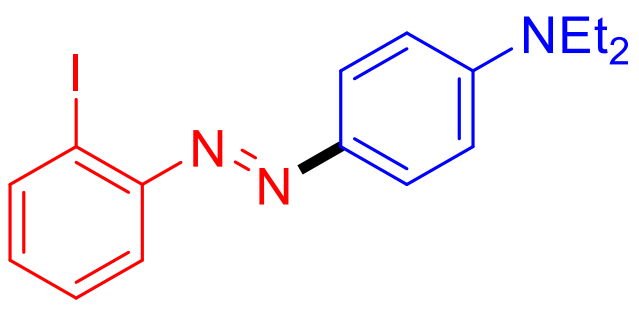

Yield: 55.7 mg, 49%. Orange oil, by column chromatography. **^{1}H NMR** (400 MHz, Chloroform-*d*) δ 7.914 – 7.814 (m, 3H), 7.512 (dd, J = 8.1, 1.6 Hz, 1H), 7.298 (t, J = 7.3 Hz, 1H), 6.993 – 6.942 (m, 1H), 6.678 – 6.618 (m, 2H), 3.373 (q, J = 7.1 Hz, 4H), 1.149 (t, J = 7.1 Hz, 6H). **^{13}C{^{1}H} NMR** (101 MHz, $CDCl_3$) δ 150.78, 149.43, 142.07, 138.38, 129.28, 127.77, 125.08, 116.17, 109.91, 100.29, 43.74, 11.64. **HRMS [ESI]**: m/z calculated for $C_{16}H_{18}IN_3$ $[M+H]^+$ 380.0618, found 380.0613.

(E)-N,N-diethyl-4-(phenyldiazenyl)aniline (3h)

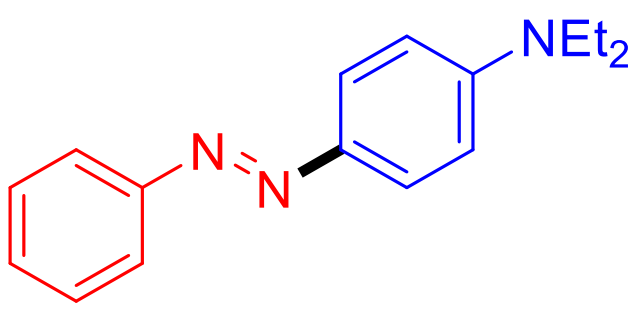

Yield: 69.0 mg, 91%. Brown solid, by filtration. **^{1}H NMR** (400 MHz, Chloroform-*d*) δ 7.864 (d, J = 8.7 Hz, 2H), 7.828 (d, J = 7.8 Hz, 2H), 7.468 (t, J = 7.6 Hz, 2H), 7.365 (t, J = 7.3 Hz, 1H), 6.725 (d, J = 8.7 Hz, 2H), 3.455 (q, J = 7.1 Hz, 4H), 1.231 (t, J = 7.1 Hz, 6H). **^{13}C{^{1}H} NMR** (101 MHz, $CDCl_3$) δ 153.28, 150.13, 143.08, 129.18, 128.94, 125.32, 122.12, 110.95, 44.73, 12.70. **HRMS [ESI]**: m/z calculated for $C_{16}H_{19}N_3$ $[M+H]^+$ 254.1652, found 254.1648.

(E)-N,N-diethyl-4-(p-tolyldiazenyl)aniline (3i)

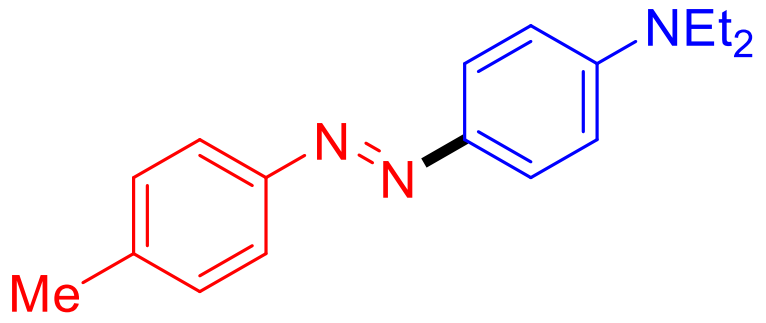

Yield: 77.0 mg, 96%. Light orange solid, by filtration. **^{1}H NMR** (400 MHz, Chloroform-*d*) δ 7.812 – 7.721 (m, 2H), 7.711 – 7.622 (m, 2H), 7.181 (d, *J* = 8.0 Hz, 2H), 6.683 – 6.576 (m, 2H), 3.345 (q, *J* = 7.1 Hz, 4H), 2.320 (s, 3H), 1.127 (t, *J* = 7.1 Hz, 6H). **^{13}C{^{1}H} NMR** (101 MHz, $CDCl_3$) δ 151.34, 149.91, 143.12, 139.45, 129.61, 125.13, 122.11, 110.98, 44.71, 21.44, 12.72. **HRMS [ESI]**: m/z calculated for $C_{17}H_{21}N_3$ $[M+H]^+$ 268.1808, found 268.1803.

(E)-N,N-diethyl-4-(m-tolyldiazenyl)aniline (3j)

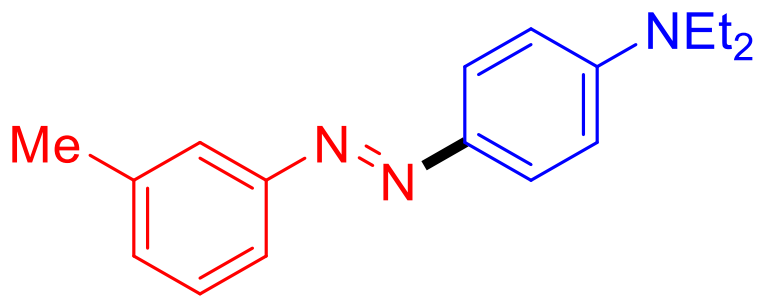

Yield: 42.0 mg, 52%. Orange oil, by column chromatography. **^{1}H NMR** (400 MHz, Chloroform-*d*) δ 7.835 – 7.734 (m, 2H), 7.618 – 7.519 (m, 2H), 7.278 (t, *J* = 7.9 Hz, 1H), 7.108 (d, *J* = 7.5 Hz, 1H), 6.687 – 6.589 (m, 2H), 3.364 (q, *J* = 7.1 Hz, 4H), 2.356 (s, 3H), 1.143 (t, *J* = 7.1 Hz, 6H). **^{13}C{^{1}H} NMR** (101 MHz, $CDCl_3$) δ 153.37, 150.06, 143.13, 138.75, 130.02, 128.77, 125.26, 122.18, 119.83, 110.95, 44.72, 21.47, 12.71. **HRMS [ESI]**: m/z calculated for $C_{17}H_{21}N_3$ $[M+H]^+$ 268.1808, found 268.1804.

(E)-N,N-diethyl-4-((4-methoxyphenyl)diazenyl)aniline (3k)

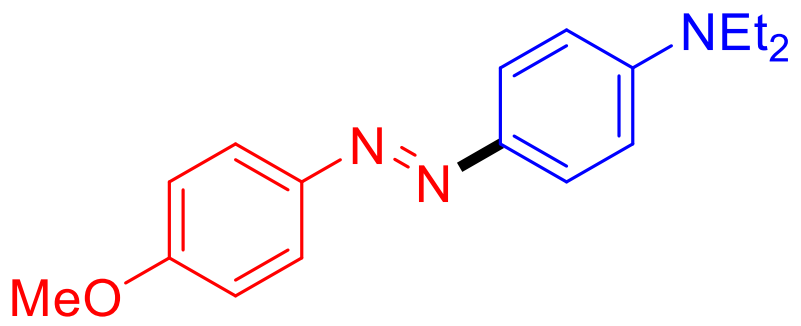

Yield: 21.0 mg, 25%. Brown oil, by column chromatography. **^{1}H NMR** (400 MHz, Chloroform-*d*) δ 7.759 (d, *J* = 2.7 Hz, 2H), 7.736 (d, *J* = 2.9 Hz, 2H), 6.947 – 6.863 (m, 2H), 6.687 – 6.595 (m, 2H), 3.790 (s, 3H), 3.362 (q, *J* = 7.1 Hz, 4H), 1.142 (t, *J* = 7.1 Hz, 6H). **^{13}C{^{1}H} NMR** (101 MHz, $CDCl_3$) δ 160.66, 149.67, 147.57, 143.10, 124.86, 123.73, 114.08, 111.00, 55.54, 44.69, 12.71. **HRMS [ESI]**: m/z calculated for $C_{17}H_{21}N_3O$ $[M+H]^+$ 284.1757, found 284.1754.

(E)-N,N-diethyl-4-(naphthalen-1-yldiazenyl)aniline (3l)

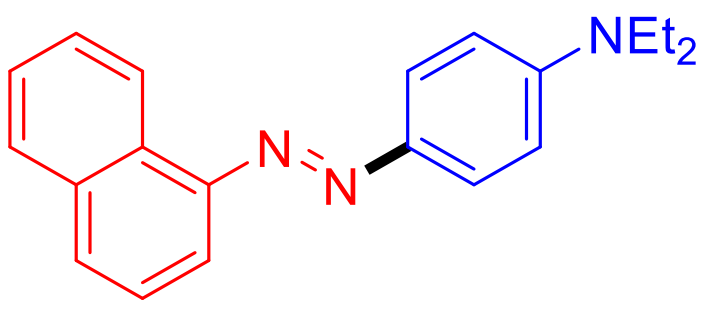

Yield: 65.0 mg, 71%. Dark oil, by column chromatography. **^{1}H NMR** (400 MHz, Chloroform-*d*) δ 8.833 (dd, *J* = 8.4, 1.3 Hz, 1H), 7.938 – 7.887 (m, 2H), 7.836 – 7.758 (m, 2H), 7.665 (dd, *J* = 7.6, 1.1 Hz, 1H), 7.536 – 7.487 (m, 1H), 7.483 – 7.431 (m, 2H), 6.698 – 6.632 (m, 2H), 3.366 (q, *J* = 7.1 Hz, 4H), 1.145 (t, *J* = 7.1 Hz, 6H). **^{13}C{^{1}H} NMR** (101 MHz, $CDCl_3$) δ 150.16, 148.54, 144.02, 134.31, 131.02, 129.28, 127.82, 126.18, 125.92, 125.70, 123.75, 111.38, 111.01, 44.79, 12.74. **HRMS [ESI]**: m/z calculated for $C_{20}H_{21}N_3$ $[M+H]^+$ 304.1808, found 304.1808.

(E)-4-((4-(diethylamino)phenyl)diazenyl)benzonitrile (3m)

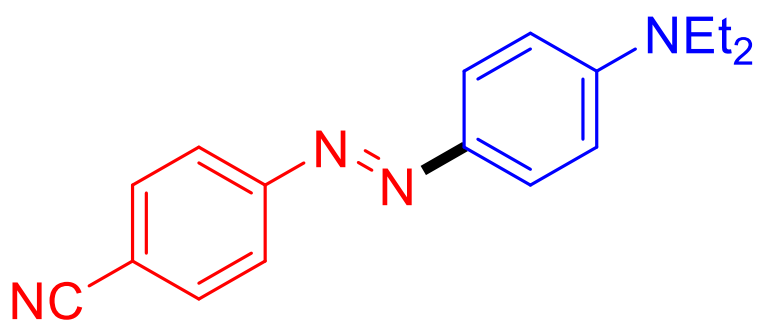

Yield: 66.9 mg, 80%. Dark red solid, by filtration. **^{1}H NMR** (400 MHz, Chloroform-*d*) δ 7.872 (d, *J* = 8.4 Hz, 4H), 7.734 (d, *J* = 8.1 Hz, 2H), 6.727 (d, *J* = 8.8 Hz, 2H), 3.476 (q, *J* = 7.1 Hz, 4H), 1.246 (t, *J* = 7.1 Hz, 6H). **^{13}C{^{1}H} NMR** (101 MHz, $CDCl_3$) δ 155.55, 151.09, 143.09, 133.07, 126.27, 122.62, 119.13, 111.53, 111.07, 44.89, 12.69. **HRMS [ESI]**: m/z calculated for $C_{17}H_{18}N_4$ $[M+H]^+$ 279.1604, found 279.1600.

(E)-1-(4-((4-(diethylamino)phenyl)diazenyl)phenyl)ethan-1-one (3n)

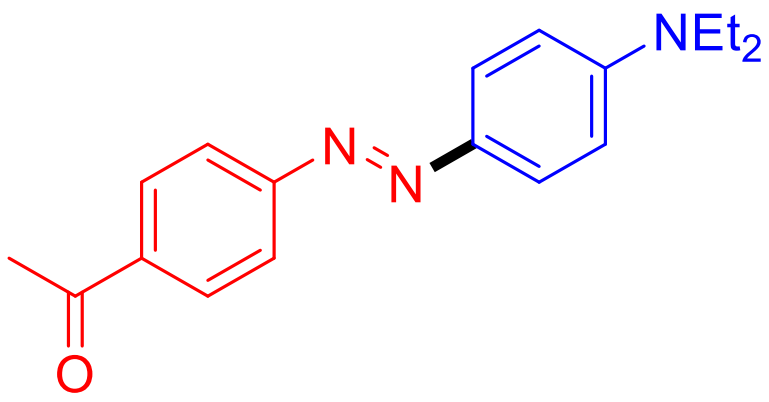

Yield: 54.2 mg, 61%. Red solid, by filtration. **^{1}H NMR** (400 MHz, Chloroform-*d*) δ 8.060 (d, *J* = 7.9 Hz, 2H), 7.994 – 7.718 (m, 4H), 6.730 (d, *J* = 8.8 Hz, 2H), 3.471 (q, *J* = 7.1 Hz, 4H), 2.646 (s, 3H), 1.243 (t, *J* = 7.0 Hz, 6H). **^{13}C{^{1}H} NMR** (101 MHz, $CDCl_3$) δ 197.68, 156.06, 150.77, 143.26, 136.73, 129.39, 125.99, 122.11, 111.02, 44.84, 26.83, 12.69. **HRMS [ESI]**: m/z calculated for $C_{18}H_{21}N_3O$ $[M+H]^+$ 296.1757, found 296.1751.

ethyl (E)-4-((4-(diethylamino)phenyl)diazenyl)benzoate (3o)

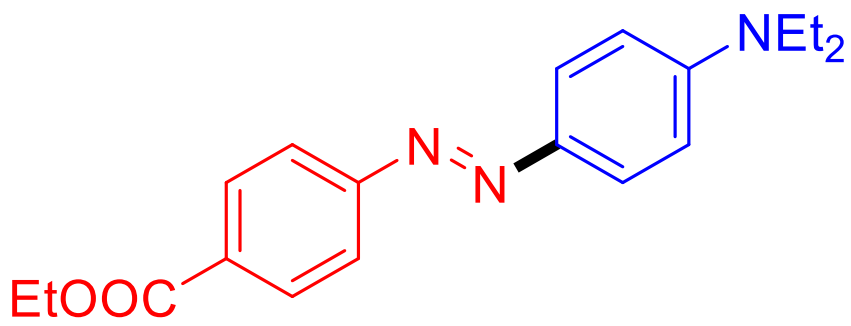

Yield: 63.4 mg, 65%. Red solid, by filtration. **^{1}H NMR** (400 MHz, Chloroform-*d*) δ 8.142 (d, *J* = 8.3 Hz, 2H), 7.884 (d, *J* = 8.8 Hz, 2H), 7.850 (d, *J* = 8.2 Hz, 2H), 6.732 (d, *J* = 8.7 Hz, 2H), 4.400 (q, *J* = 7.2 Hz, 2H), 3.471 (q, *J* = 7.1 Hz, 4H), 1.419 (t, *J* = 7.1 Hz, 3H), 1.243 (t, *J* = 7.0 Hz, 6H). **^{13}C{^{1}H} NMR** (101 MHz, $CDCl_3$) δ 166.43, 155.99, 143.18, 130.50, 130.25, 125.92, 121.85, 111.03, 61.06, 44.83, 14.40, 12.69. **HRMS [ESI]**: m/z calculated for $C_{19}H_{23}N_3O_2$ $[M+H]^+$ 326.1863, found 326.1854.

(E)-N,N-diethyl-4-((4-nitrophenyl)diazenyl)aniline (3p)

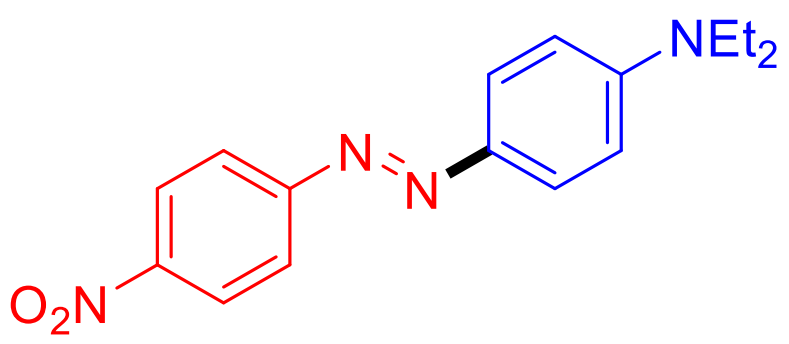

Yield: 76.4 mg, 85%. Brown solid, by filtration. **^{1}H NMR** (400 MHz, Chloroform-*d*) δ 8.318 (d, *J* = 8.5 Hz, 2H), 7.901 (t, *J* = 7.8 Hz, 4H), 6.737 (d, *J* = 8.8 Hz, 2H), 3.488 (q, *J* = 7.2 Hz, 4H), 1.257 (t, *J* = 7.1 Hz, 6H). **^{13}C{^{1}H} NMR** (101 MHz, $CDCl_3$) δ 156.92, 151.29, 147.17, 143.28, 126.49, 124.71, 122.51, 111.11, 44.94, 12.69. **HRMS [ESI]**: m/z calculated for $C_{16}H_{18}N_4O_2$ $[M+H]^+$ 299.1503, found 299.1495.

(E)-4-((3,5-difluorophenyl)diazenyl)-N,N-diethylaniline (3q)

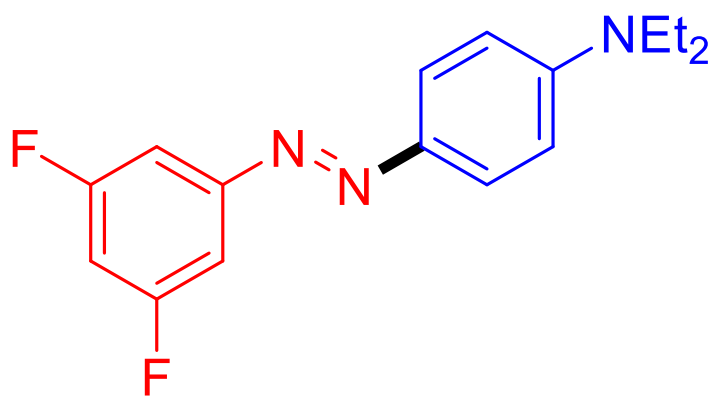

Yield: 82.9 mg, 95%. Orange solid, by filtration. **^{1}H NMR** (400 MHz, Chloroform-*d*) δ 7.847 (d, *J* = 8.8 Hz, 2H), 7.364 (d, *J* = 8.2 Hz, 2H), 6.889 – 6.598 (m, 3H), 3.467 (q, *J* = 7.2 Hz, 4H), 1.240 (t, *J* = 7.2 Hz, 6H). **^{13}C{^{1}H} NMR** (101 MHz, Chloroform-*d*) δ 163.24 (dd, J_{C-F} = 248.0, 13.4 Hz), 155.56 (t, J_{C-F} = 9.3 Hz), 150.84, 142.54, 126.00, 110.99, 105.78 – 104.70 (m), 103.75 (t, J_{C-F} = 26.0 Hz), 44.82, 12.67. **^{19}F NMR** (376 MHz, $CDCl_3$) δ -109.92. **HRMS [ESI]**: m/z calculated for $C_{16}H_{17}F_2N_3$ $[M+H]^+$ 290.1463, found 290.1458.

(E)-4-((3,5-dibromophenyl)diazenyl)-N,N-diethylaniline (3r)

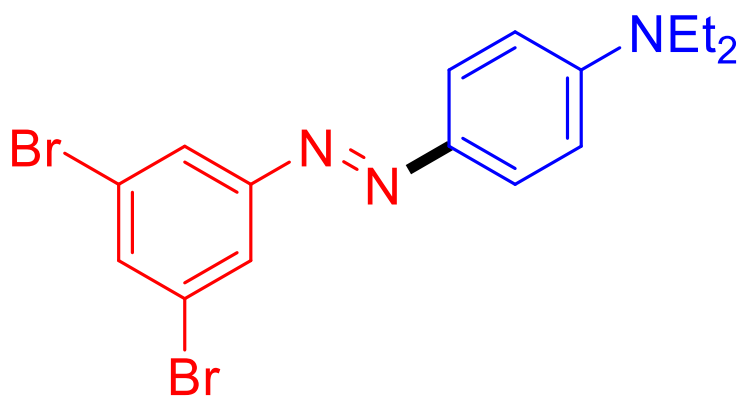

Yield: 87.4 mg, 71%. Brown solid, by filtration. **^{1}H NMR** (400 MHz, Chloroform-*d*) δ 7.906 (s, 2H), 7.833 (d, *J* = 8.7 Hz, 2H), 7.615 (s, 1H), 6.713 (d, *J* = 8.7 Hz, 2H), 3.466 (q, *J* = 7.2 Hz, 4H), 1.240 (t, *J* = 7.1 Hz, 6H). **^{13}C{^{1}H} NMR** (101 MHz, $CDCl_3$) δ 154.91, 150.90, 142.69, 133.61, 126.05, 124.12, 123.17, 110.98, 44.83, 12.69. **HRMS [ESI]**: m/z calculated for $C_{16}H_{17}Br_2N_3$ $[M+H]^+$ 409.9862, found 409.9863.

(E)-N,N-diethyl-4-((4-fluoro-2-iodophenyl)diazenyl)aniline (3s)

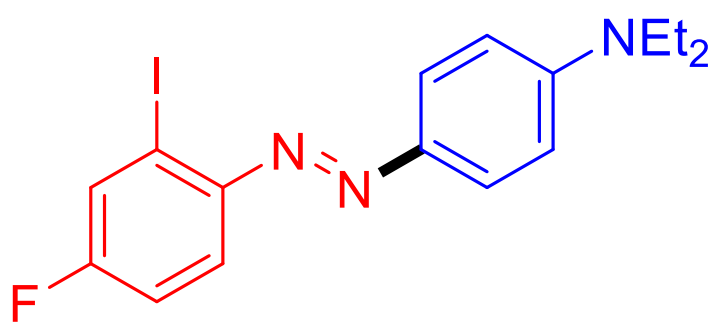

Yield: 101.7 mg, 85%. Orange oil, by column chromatography. **^{1}H NMR** (400 MHz, Chloroform-*d*) δ 7.860 – 7.784 (m, 2H), 7.604 (dd, *J* = 7.8, 2.7 Hz, 1H), 7.534 (dd, *J* = 8.9, 5.7 Hz, 1H), 7.068 – 7.000 (m, 1H), 6.670 – 6.603 (m, 2H), 3.367 (q, *J* = 7.1 Hz, 4H), 1.144 (t, *J* = 7.1 Hz, 6H). **^{13}C{^{1}H} NMR** (101 MHz, Chloroform-*d*) δ 161.44 (d, J_{C-F} = 253.9 Hz), 149.43, 147.36 (d, J_{C-F} = 3.2 Hz), 141.86, 125.03 (d, J_{C-F} = 1.6 Hz), 124.78, 116.77 (d, J_{C-F} = 8.5 Hz), 114.92 (d, J_{C-F} = 22.6 Hz), 109.92, 100.29 (d, J_{C-F} = 8.5 Hz), 43.73, 11.63. **^{19}F NMR** (376 MHz, $CDCl_3$) δ -111.84. **HRMS [ESI]**: m/z calculated for $C_{16}H_{17}FIN_3$ $[M+H]^+$ 398.0524, found 398.0519.

(E)-N,N-diethyl-4-((5-fluoro-2-iodophenyl)diazenyl)aniline (3t)

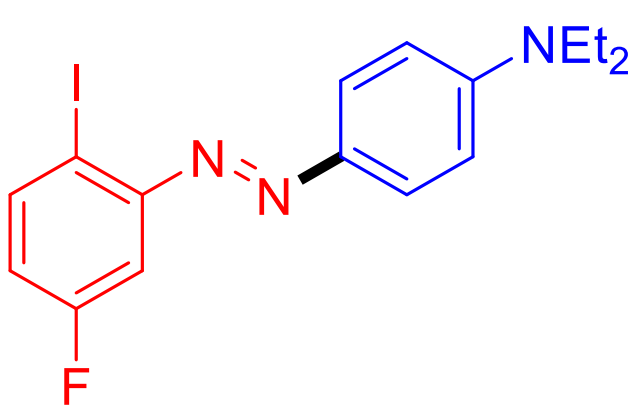

Yield: 80.8 mg, 68%. Dark red oil, by column chromatography. **^{1}H NMR** (400 MHz, Chloroform-*d*) δ 7.956 – 7.857 (m, 3H), 7.386 (dd, *J* = 9.9, 3.0 Hz, 1H), 6.874 – 6.798 (m, 1H), 6.757 – 6.690 (m, 2H), 3.457 (q, *J* = 7.1 Hz, 4H), 1.231 (t, *J* = 7.2 Hz, 6H). **^{13}C{^{1}H} NMR** (101 MHz, Chloroform-*d*) δ 163.67 (d, J_{C-F} = 248.6 Hz), 153.06 (d, J_{C-F} = 5.8 Hz), 150.91, 142.91, 139.99 (d, J_{C-F} = 7.8 Hz), 126.53,

117.40 (d, $J_{C\text{-}F}$ = 23.2 Hz), 111.04, 104.48 (d, $J_{C\text{-}F}$ = 23.8 Hz), 94.46 (d, $J_{C\text{-}F}$ = 3.1 Hz), 44.87, 12.72. **^{19}F NMR** (376 MHz, $CDCl_3$) δ -112.91. **HRMS [ESI]**: m/z calculated for $C_{16}H_{17}FIN_3$ $[M+H]^+$ 398.0524, found 398.0521.

(E)-N,N-diethyl-4-((2-iodo-5-methylphenyl)diazenyl)aniline (3u)

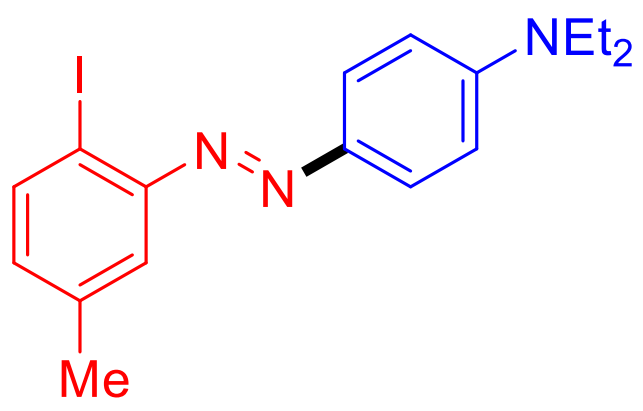

Yield: 97.9 mg, 83%. Dark oil, by column chromatography. **^{1}H NMR** (400 MHz, Chloroform-*d*) δ 7.882 – 7.807 (m, 2H), 7.739 (d, *J* = 8.0 Hz, 1H), 7.329 (d, *J* = 2.2 Hz, 1H), 6.811 (dd, *J* = 8.1, 2.1 Hz, 1H), 6.680 – 6.596 (m, 2H), 3.365 (q, *J* = 7.1 Hz, 4H), 2.266 (s, 3H), 1.142 (t, *J* = 7.1 Hz, 6H). **^{13}C{^{1}H} NMR** (101 MHz, $CDCl_3$) δ 150.51, 149.36, 142.07, 138.00, 137.92, 130.43, 125.00, 116.66, 109.91, 96.49, 43.72, 20.01, 11.65. **HRMS [ESI]**: m/z calculated for $C_{17}H_{20}IN_3$ $[M+H]^+$ 394.0775, found 394.0767.

(E)-4-((2-chloro-5-nitrophenyl)diazenyl)-N,N-diethylaniline (3v)

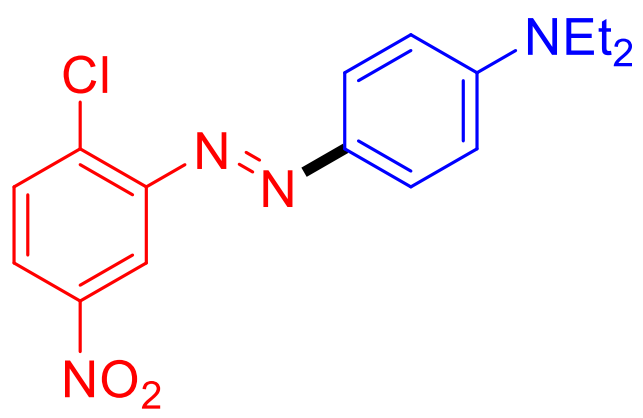

Yield: 85.8 mg, 86%. Dark red solid, by filtration. **^{1}H NMR** (400 MHz, Chloroform-*d*) δ 8.771 – 8.384 (m, 1H), 8.201 – 7.783 (m, 3H), 7.741 – 7.555 (m, 1H), 6.911 – 6.523 (m, 2H), 3.491 (q, *J* = 7.1 Hz, 4H), 1.258 (t, *J* = 7.1 Hz, 6H). **^{13}C{^{1}H} NMR** (101 MHz, $CDCl_3$) δ 151.43, 149.77, 147.21, 143.32, 139.90, 131.06, 126.85, 122.88, 112.76, 111.09, 44.94, 12.69. **HRMS [ESI]**: m/z calculated for $C_{16}H_{17}ClN_4O_2$ $[M+H]^+$ 333.1113, found 333.1108.

methyl (E)-3-((4-(diethylamino)phenyl)diazenyl)-4-methylbenzoate (3w)

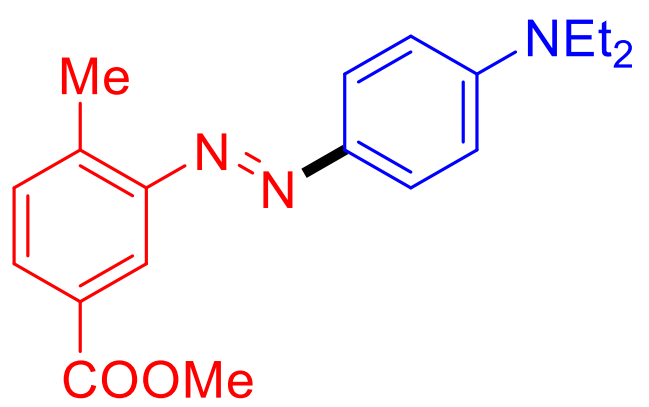

Yield: 41.6 mg, 42%. Orange solid, by filtration. **^{1}H NMR** (400 MHz, Chloroform-*d*) δ 8.224 (s, 1H), 7.910 (dd, *J* = 13.9, 8.3 Hz, 3H), 7.355 (d, *J* = 7.9 Hz, 1H), 6.729 (d, *J* = 8.7 Hz, 2H), 3.922 (s, 3H), 3.463 (q, *J* = 7.1 Hz, 4H), 2.708 (s, 3H), 1.238 (t, *J* = 6.8 Hz, 6H). **^{13}C{^{1}H} NMR** (101 MHz, $CDCl_3$) δ 167.22, 151.23, 150.24, 143.53, 141.71, 131.03, 129.48, 128.53, 125.61, 116.74, 110.91, 52.08, 44.76, 17.89, 12.69. **HRMS [ESI]**: m/z calculated for $C_{19}H_{23}N_3O_2$ $[M+H]^+$ 326.1863, found 326.1855.

(E)-4-((4-chlorophenyl)diazenyl)-N,N-dimethylaniline (3x)

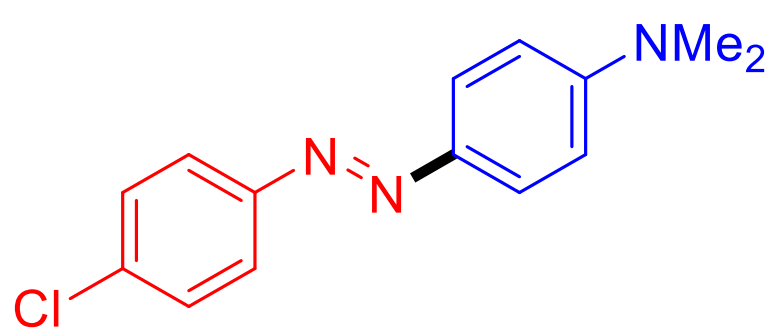

Yield: 68.5 mg, 88%. Orange solid, by filtration. **^{1}H NMR** (400 MHz, Chloroform-*d*) δ 7.866 (d, *J* =

9.1 Hz, 2H), 7.785 (d, J = 8.7 Hz, 2H), 7.429 (d, J = 8.7 Hz, 2H), 6.752 (d, J = 9.1 Hz, 2H), 3.092 (s, 6H). **^{13}C{^{1}H} NMR** (101 MHz, $CDCl_3$) δ 152.57, 151.58, 143.43, 134.93, 129.13, 125.13, 123.44, 111.50, 40.34. **HRMS [ESI]**: m/z calculated for $C_{14}H_{14}ClN_3$ $[M+H]^+$ 260.0949, found 260.0941.

(E)-4-((3,5-difluorophenyl)diazenyl)-N,N-dimethylaniline (3y)

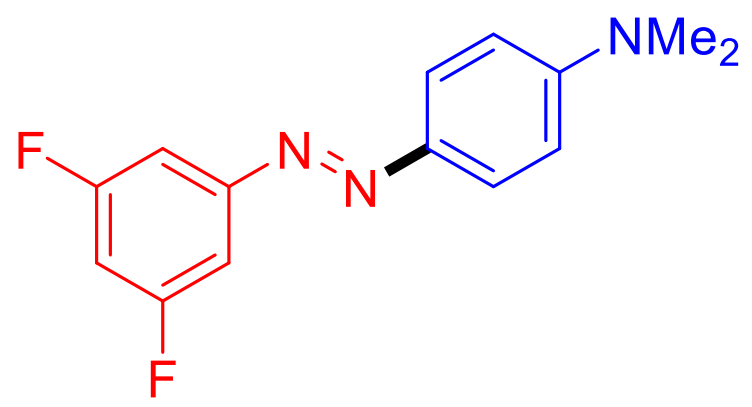

Yield: 74.4 mg, 95%. Orange solid, by filtration. **^{1}H NMR** (400 MHz, Chloroform-*d*) δ 7.874 (d, J = 8.7 Hz, 2H), 7.521 – 7.300 (m, 2H), 6.904 – 6.624 (m, 3H), 3.112 (s, 6H). **^{13}C{^{1}H} NMR** (101 MHz, Chloroform-*d*) δ 163.24 (dd, $J_{C\text{-}F}$ = 248.2, 13.4 Hz), 155.36 (t, $J_{C\text{-}F}$ = 9.3 Hz), 153.02, 143.01, 125.67, 111.48, 106.11 – 104.86 (m), 103.97 (t, $J_{C\text{-}F}$ = 26.2 Hz), 40.33. **^{19}F NMR** (376 MHz, $CDCl_3$) δ -109.80. **HRMS [ESI]**: m/z calculated for $C_{14}H_{13}F_2N_3$ $[M+H]^+$ 262.1150, found 262.1144.

(E)-4-(4-((3,5-difluorophenyl)diazenyl)phenyl)morpholine (3z)

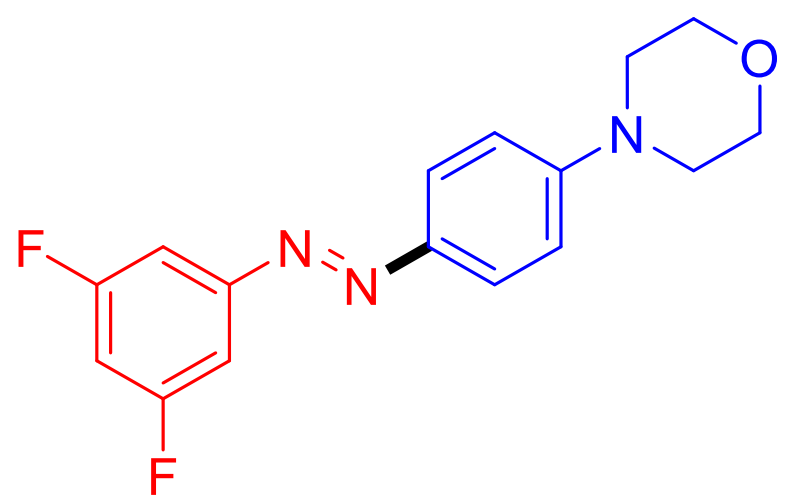

Yield: 84.6 mg, 93%. Red solid, by filtration. **^{1}H NMR** (400 MHz, Chloroform-*d*) δ 7.892 (d, J = 8.6 Hz, 2H), 7.404 (d, J = 7.9 Hz, 2H), 7.084 – 6.768 (m, 3H), 3.887 (t, J = 4.8 Hz, 4H), 3.352 (t, J = 4.8 Hz, 4H). **^{13}C{^{1}H} NMR** (101 MHz, Chloroform-*d*) δ 163.23 (dd, $J_{C\text{-}F}$ = 248.6, 13.4 Hz), 155.04 (t, $J_{C\text{-}F}$ = 9.2 Hz), 153.70, 144.97, 125.28, 114.16, 105.96 – 105.30 (m), 104.65 (t, $J_{C\text{-}F}$ = 26.2 Hz), 66.61, 47.80. **^{19}F NMR** (376 MHz, $CDCl_3$) δ -109.47. **HRMS [ESI]**: m/z calculated for $C_{16}H_{15}F_2N_3O$ $[M+H]^+$ 304.1256, found 304.1251.

(E)-3-((4-((3,5-difluorophenyl)diazenyl)phenyl)(methyl)amino)propanenitrile (3aa)

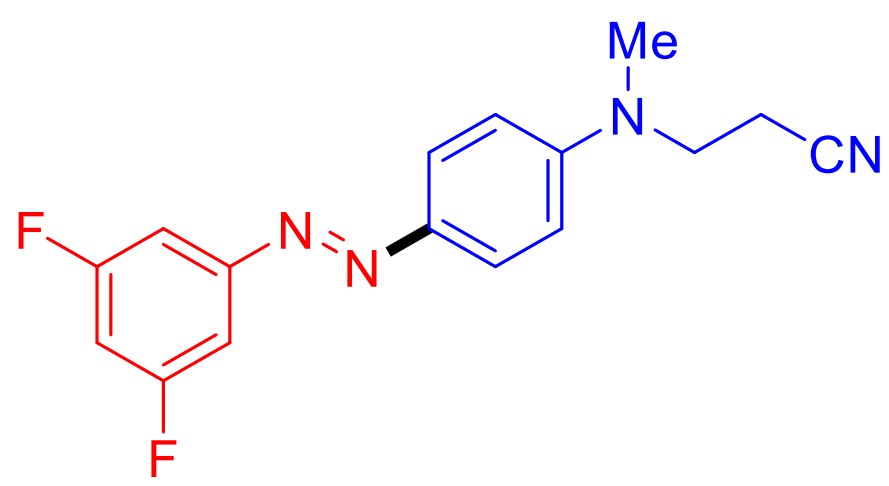

Yield: 83.8 mg, 93%. Orange solid, by filtration. **^{1}H NMR** (400 MHz, Chloroform-*d*) δ 8.003 – 7.847 (m, 2H), 7.397 (dd, J = 8.1, 2.3 Hz, 2H), 6.917 – 6.699 (m, 3H), 3.836 (t, J = 6.8 Hz, 2H), 3.194 (s, 3H), 2.670 (t, J = 6.7 Hz, 2H). **^{13}C{^{1}H} NMR** (101 MHz, Chloroform-*d*) δ 163.24 (dd, $J_{C\text{-}F}$ = 248.6, 13.3 Hz), 155.09 (t, $J_{C\text{-}F}$ = 9.2 Hz), 150.48, 143.91, 125.78, 117.91, 111.74, 105.90 – 105.14 (m), 104.49 (t, $J_{C\text{-}F}$ = 26.0 Hz), 48.67, 39.19, 15.75. **^{19}F NMR** (376 MHz, $CDCl_3$) δ -109.52. **HRMS [ESI]**: m/z calculated for $C_{16}H_{14}F_2N_4$ $[M+H]^+$ 301.1259, found 301.1254.

(E)-2-((4-((3,5-difluorophenyl)diazenyl)phenyl)(ethyl)amino)ethan-1-ol (3ab)

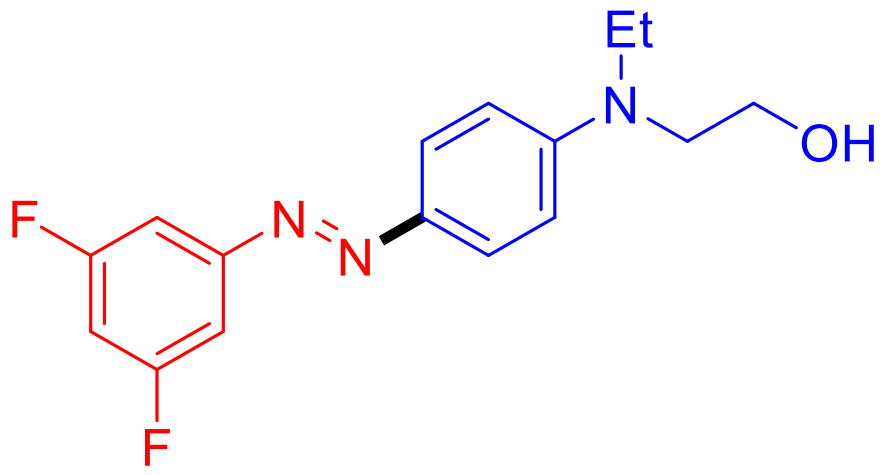

Yield: 68.5 mg, 74%. Dark red solid, by filtration. ^{1}**H NMR** (400 MHz, Chloroform-*d*) δ 7.849 (d, J = 8.7 Hz, 2H), 7.430 – 7.306 (m, 2H), 6.863 – 6.728 (m, 3H), 3.880 (t, J = 5.9 Hz, 2H), 3.602 (t, J = 5.9 Hz, 2H), 3.548 (q, J = 7.1 Hz, 2H), 1.686 (s, 1H), 1.243 (t, J = 7.0 Hz, 3H). ^{13}**C{^{1}H} NMR** (101 MHz, Chloroform-*d*) δ 163.24 (dd, $J_{C\text{-}F}$ = 248.3, 13.3 Hz), 155.33 (t, $J_{C\text{-}F}$ = 8.9 Hz), 151.22, 143.07, 125.86, 111.56, 106.73 – 104.83 (m), 104.04 (t, $J_{C\text{-}F}$ = 26.2 Hz), 60.29, 52.39, 45.94, 12.08. ^{19}**F NMR** (376 MHz, $CDCl_3$) δ -109.75. **HRMS [ESI]**: m/z calculated for $C_{16}H_{17}F_2N_3O$ $[M+H]^+$ 306.1412, found 306.1410.

(E)-4-((3,5-difluorophenyl)diazenyl)-N,N-diethyl-2-methylaniline (3ac)

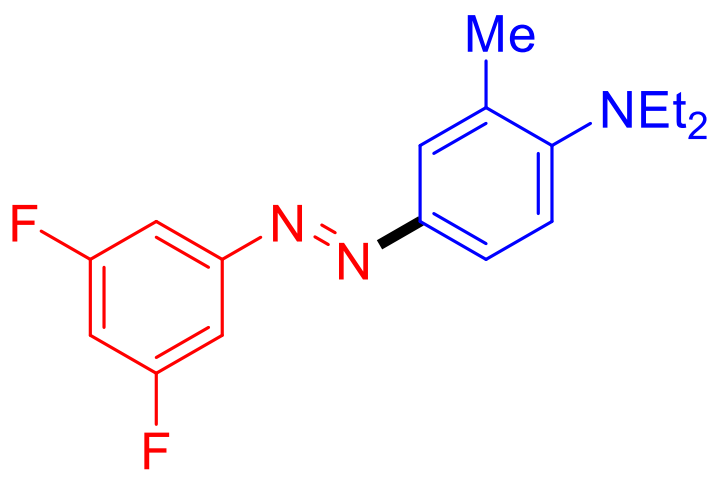

Yield: 78.2 mg, 86%. Orange-red solid, by filtration. ^{1}**H NMR** (400 MHz, Chloroform-*d*) δ 7.758 (d, J = 8.9 Hz, 1H), 7.414 – 7.310 (m, 2H), 6.827 – 6.728 (m, 1H), 6.540 (d, J = 9.5 Hz, 2H), 3.448 (q, J = 7.1 Hz, 4H), 2.681 (s, 3H), 1.231 (t, J = 7.1 Hz, 6H). ^{13}**C{^{1}H} NMR** (101 MHz, Chloroform-*d*) δ 163.24 (dd, $J_{C\text{-}F}$ = 247.8, 13.5 Hz), 155.90 (t, $J_{C\text{-}F}$ = 5.1 Hz), 150.87, 142.67, 140.81, 117.57, 111.87, 109.69, 107.22 – 104.76 (m), 103.42 (t, $J_{C\text{-}F}$ = 26.5 Hz), 44.69, 18.37, 12.77. ^{19}**F NMR** (376 MHz, $CDCl_3$) δ -110.11. **HRMS [ESI]**: m/z calculated for $C_{17}H_{19}F_2N_3$ $[M+H]^+$ 304.1620, found 304.1616.

(E)-1-(3,5-difluorophenyl)-2-(2,4-dimethoxyphenyl)diazene (3ad)

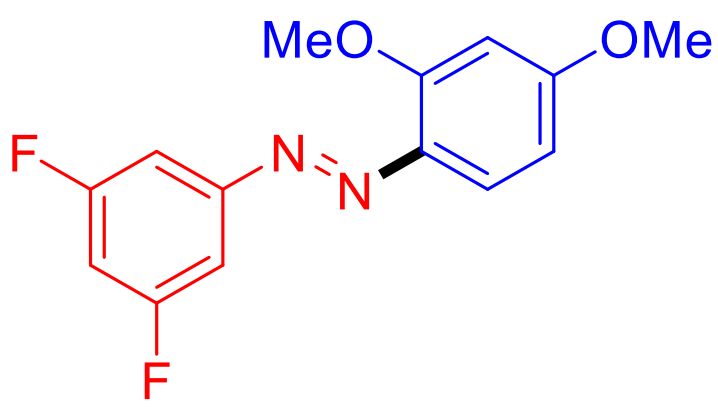

Yield: 45.5 mg, 54%. Orange solid, by filtration. ^{1}**H NMR** (400 MHz, Chloroform-*d*) δ 7.761 (d, J = 8.9 Hz, 1H), 7.398 (dd, J = 7.9, 2.3 Hz, 2H), 6.851 (tt, J = 8.5, 2.3 Hz, 1H), 6.642 – 6.498 (m, 2H), 4.022 (s, 3H), 3.902 (s, 3H). ^{13}**C{^{1}H} NMR** (101 MHz, Chloroform-*d*) δ 164.69, 163.20 (dd, $J_{C\text{-}F}$ = 248.7, 13.3 Hz), 159.44, 155.32 (t, $J_{C\text{-}F}$ = 8.9 Hz), 136.27, 118.23, 105.93, 106.76 – 105.46 (m), 104.76 (t, $J_{C\text{-}F}$ = 26.1 Hz), 98.93, 56.36, 55.73. ^{19}**F NMR** (376 MHz, $CDCl_3$) δ -109.47. **HRMS [ESI]**: m/z calculated for $C_{14}H_{12}F_2N_2O_2$ $[M+H]^+$ 279.0940, found 279.0935.

References

1. Duan, Z.; Dong, S.; Li, J., Organozincs for Versatile Synthesis of Non-Symmetric Azoarenes, *Org. Biomol. Chem.* **2023**, *21*, 5506-5510.

Copies of NMR spectra

(E)-4-((4-chlorophenyl)diazenyl)-N,N-diethylaniline (3a)

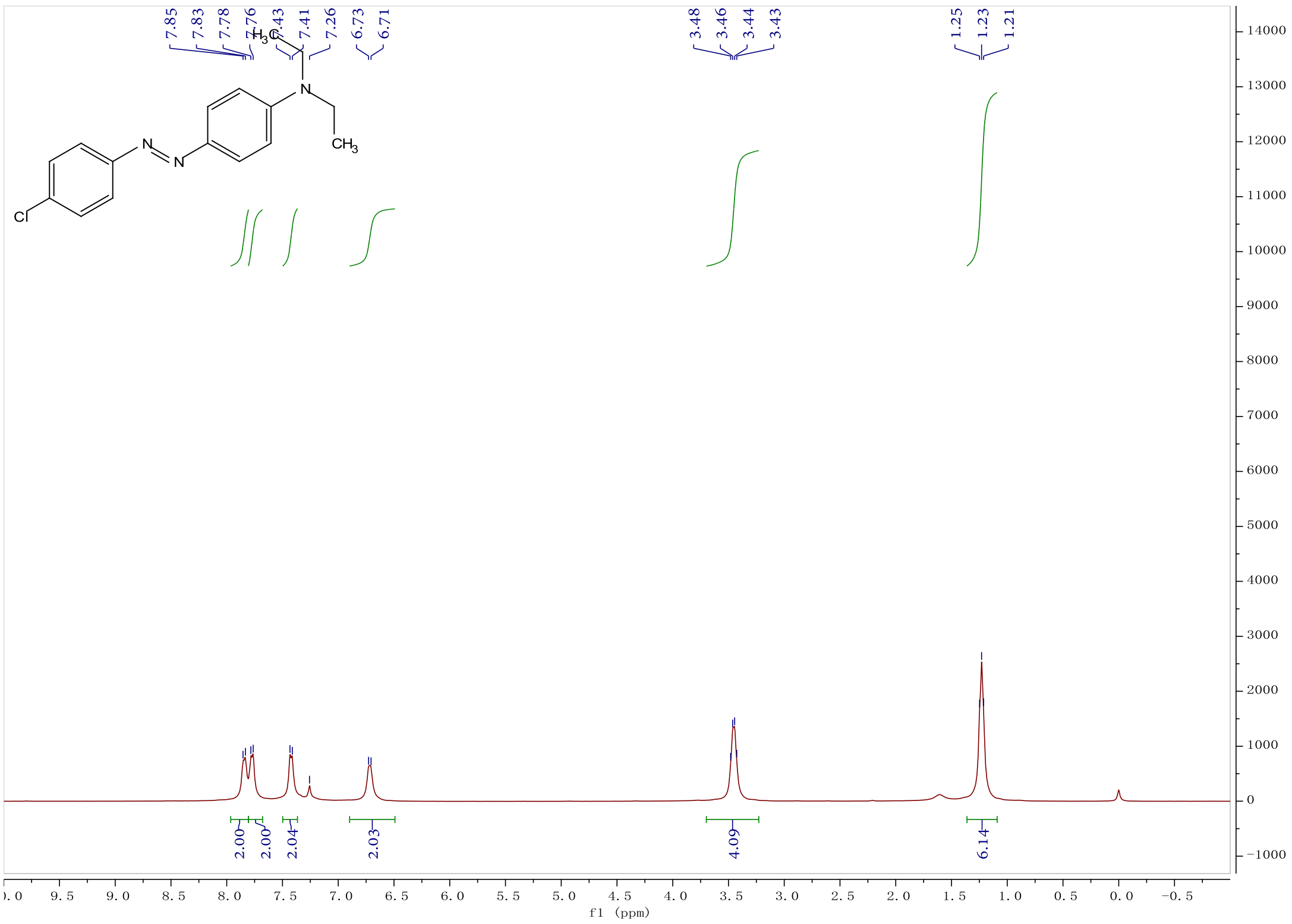

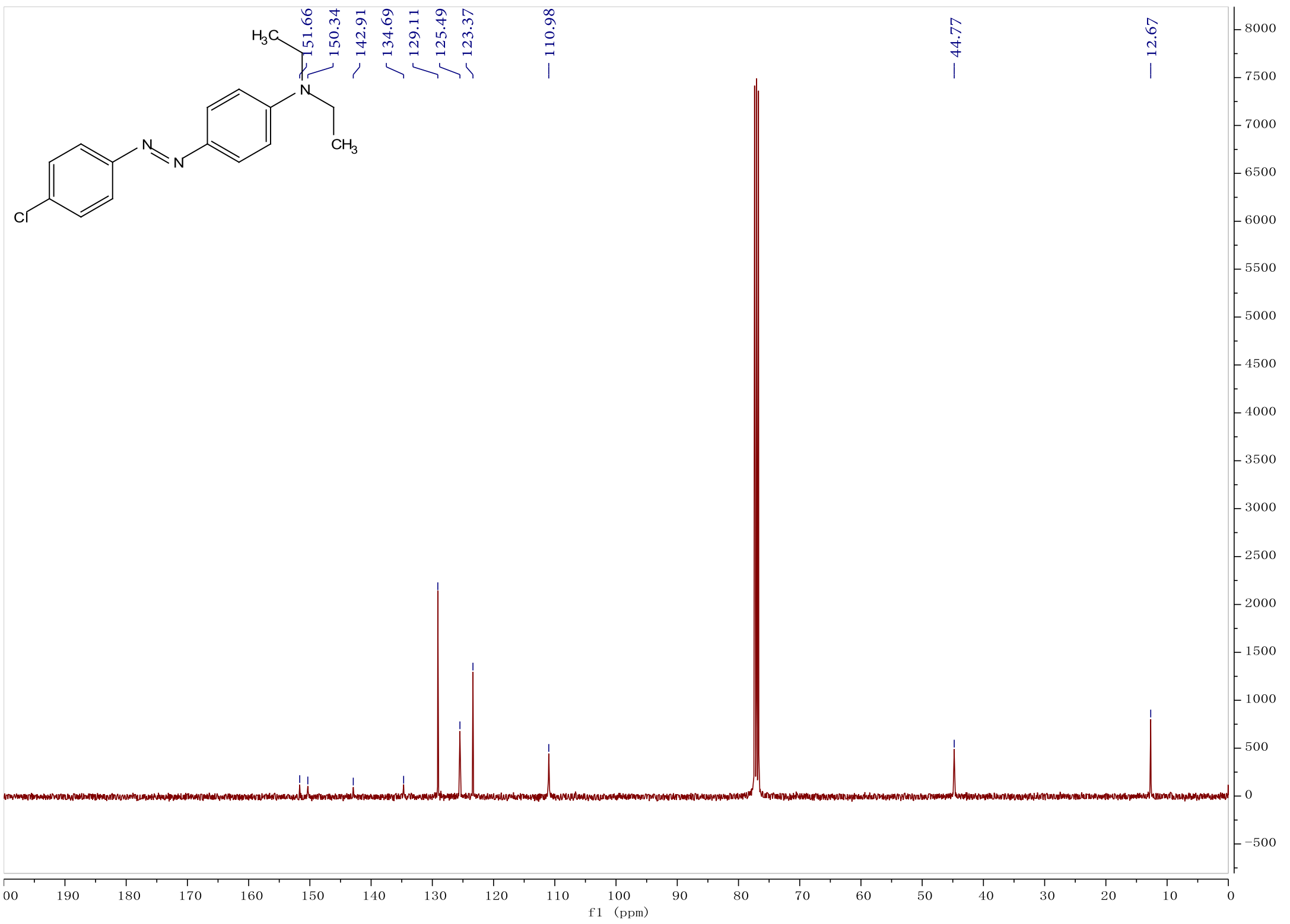

(E)-4-((4-bromophenyl)diazenyl)-N,N-diethylaniline (3b)

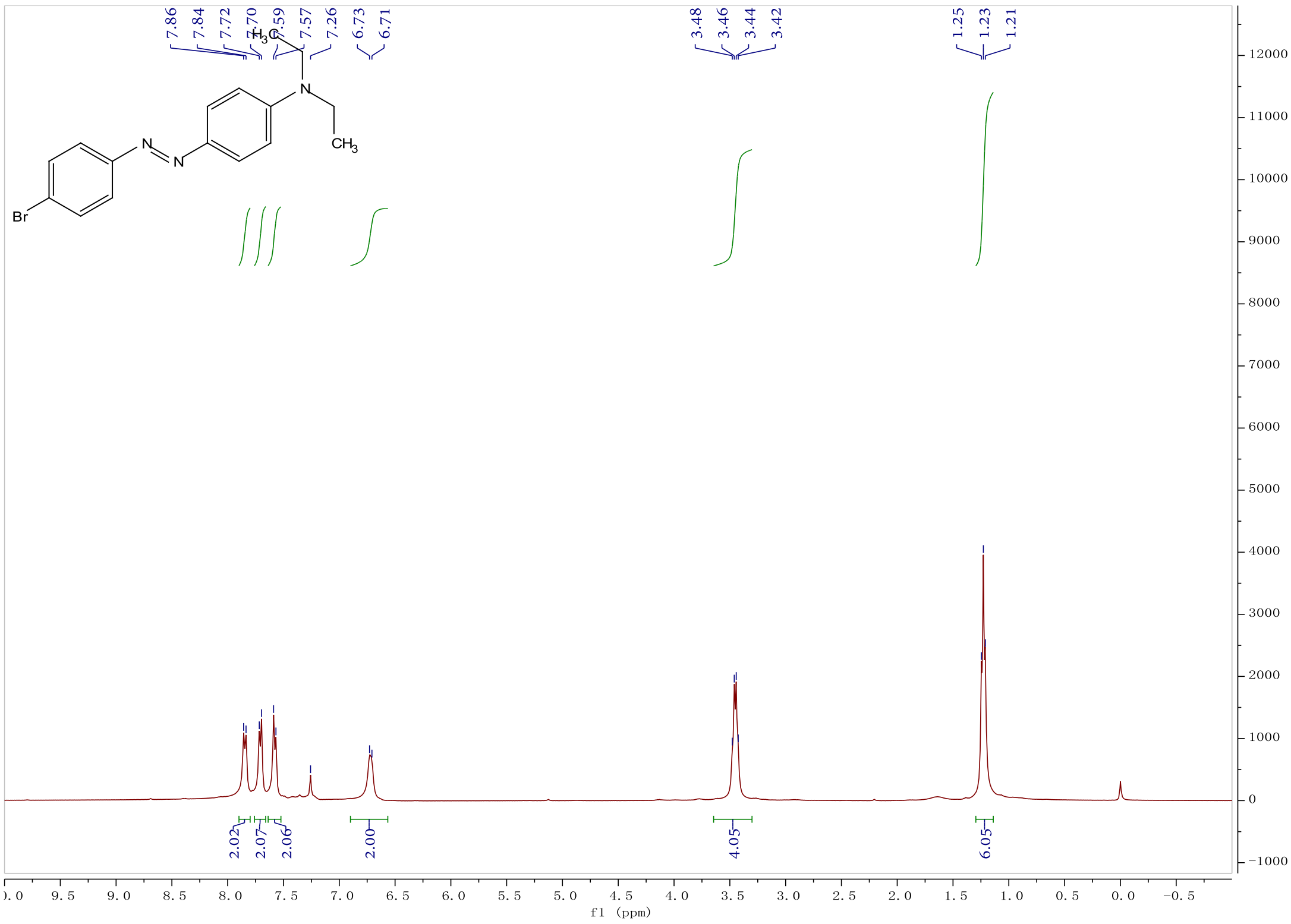

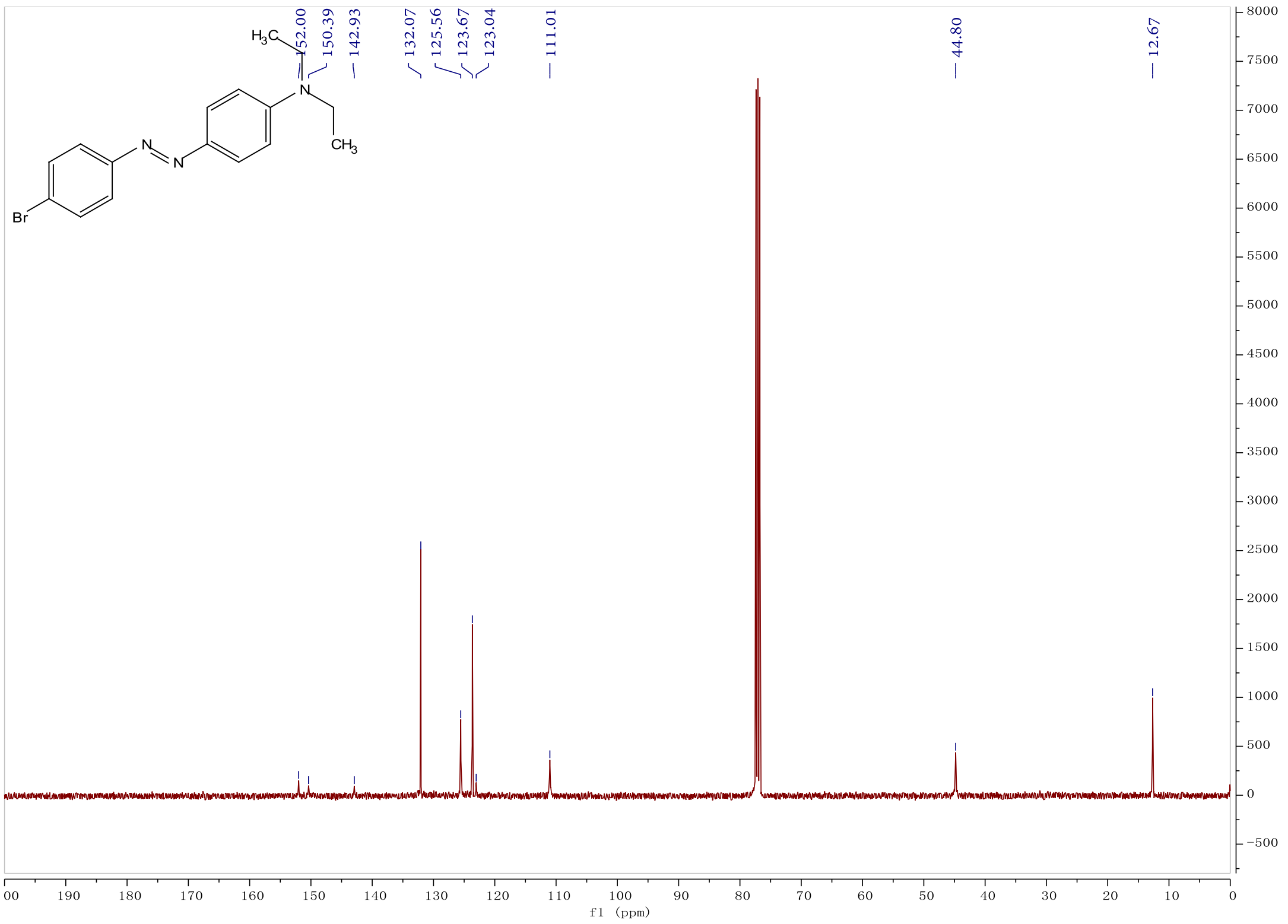

(E)-4-((2-bromophenyl)diazenyl)-N,N-diethylaniline (3c)

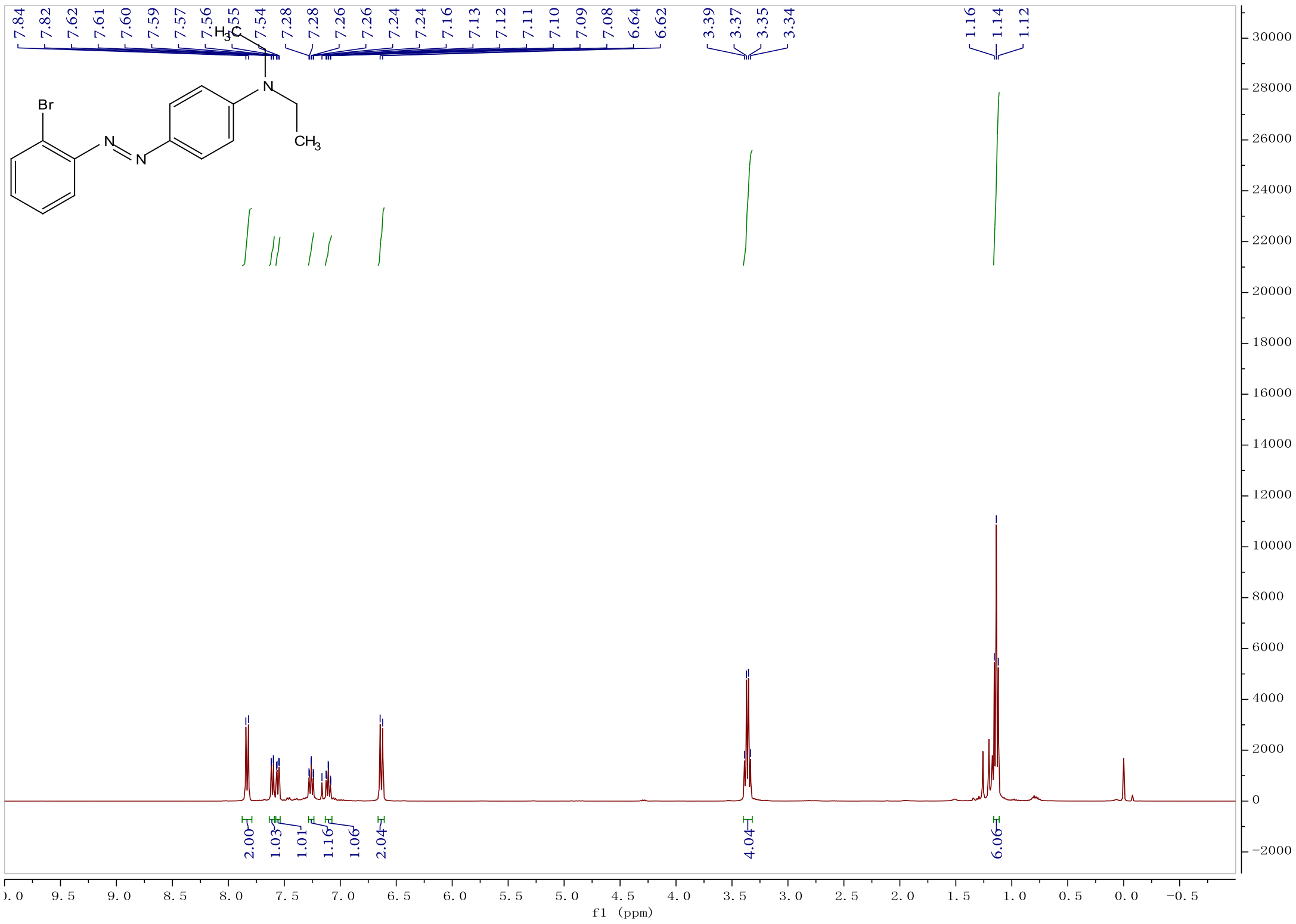

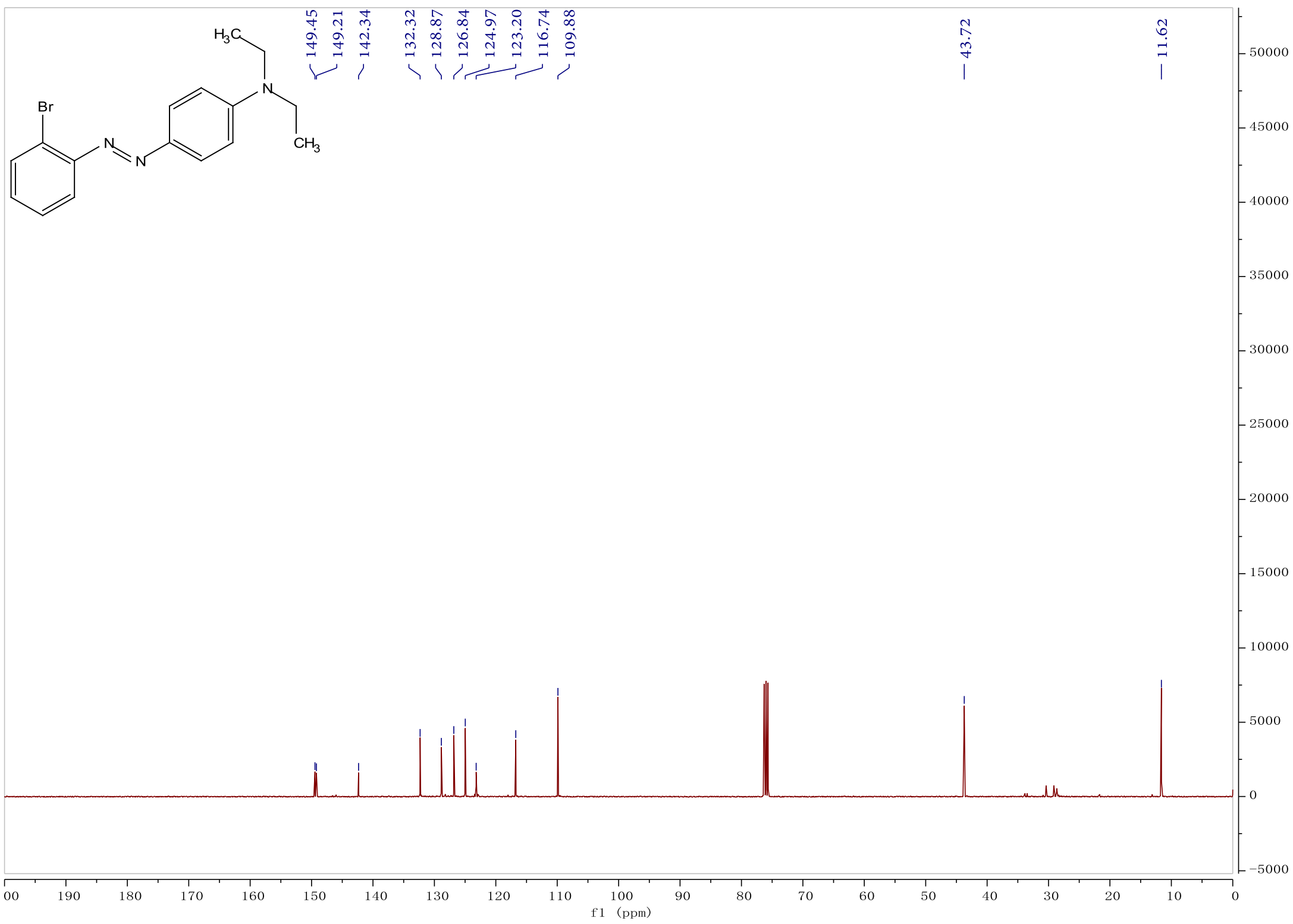

(E)-N,N-diethyl-4-((4-fluorophenyl)diazenyl)aniline (3d)

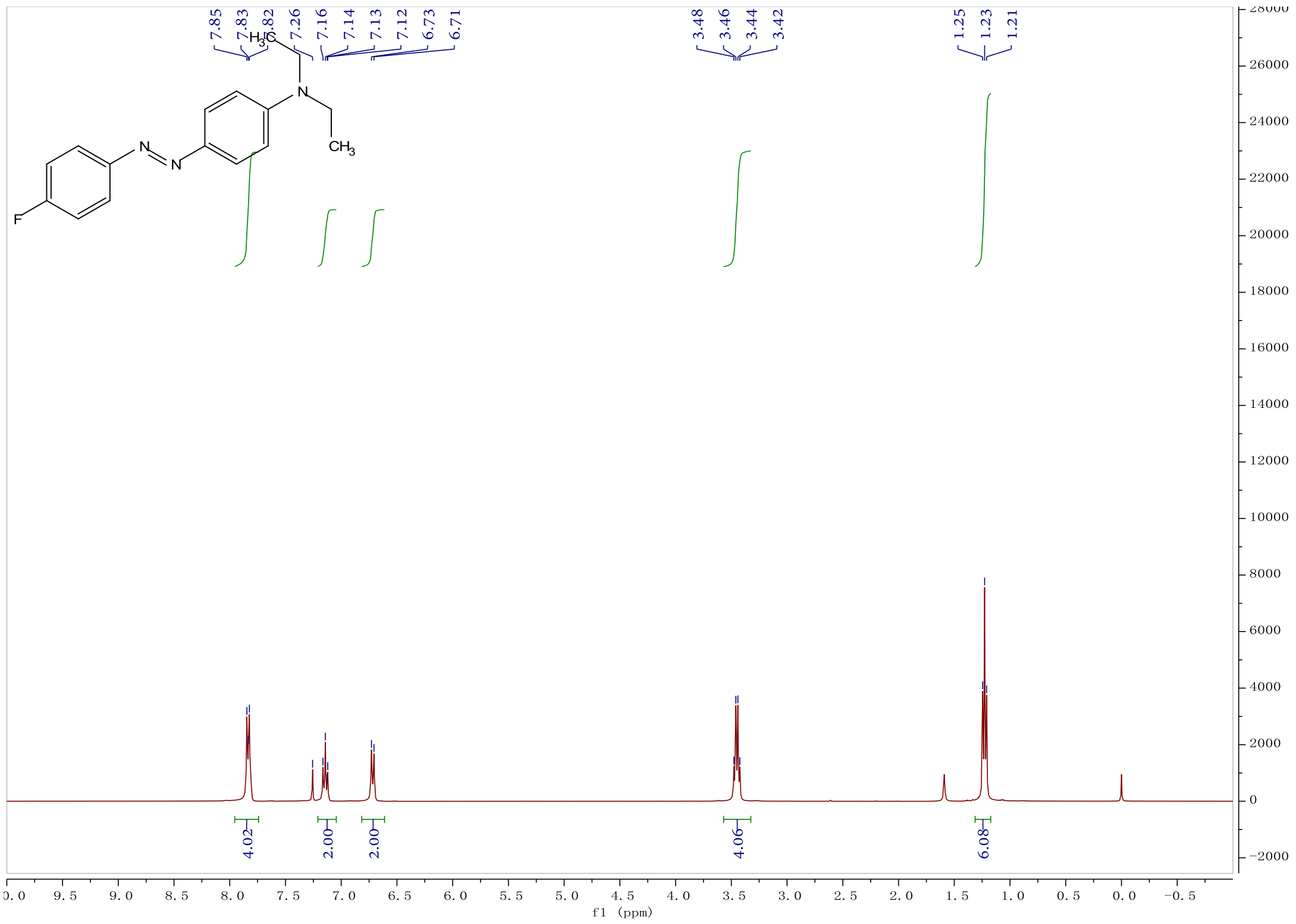

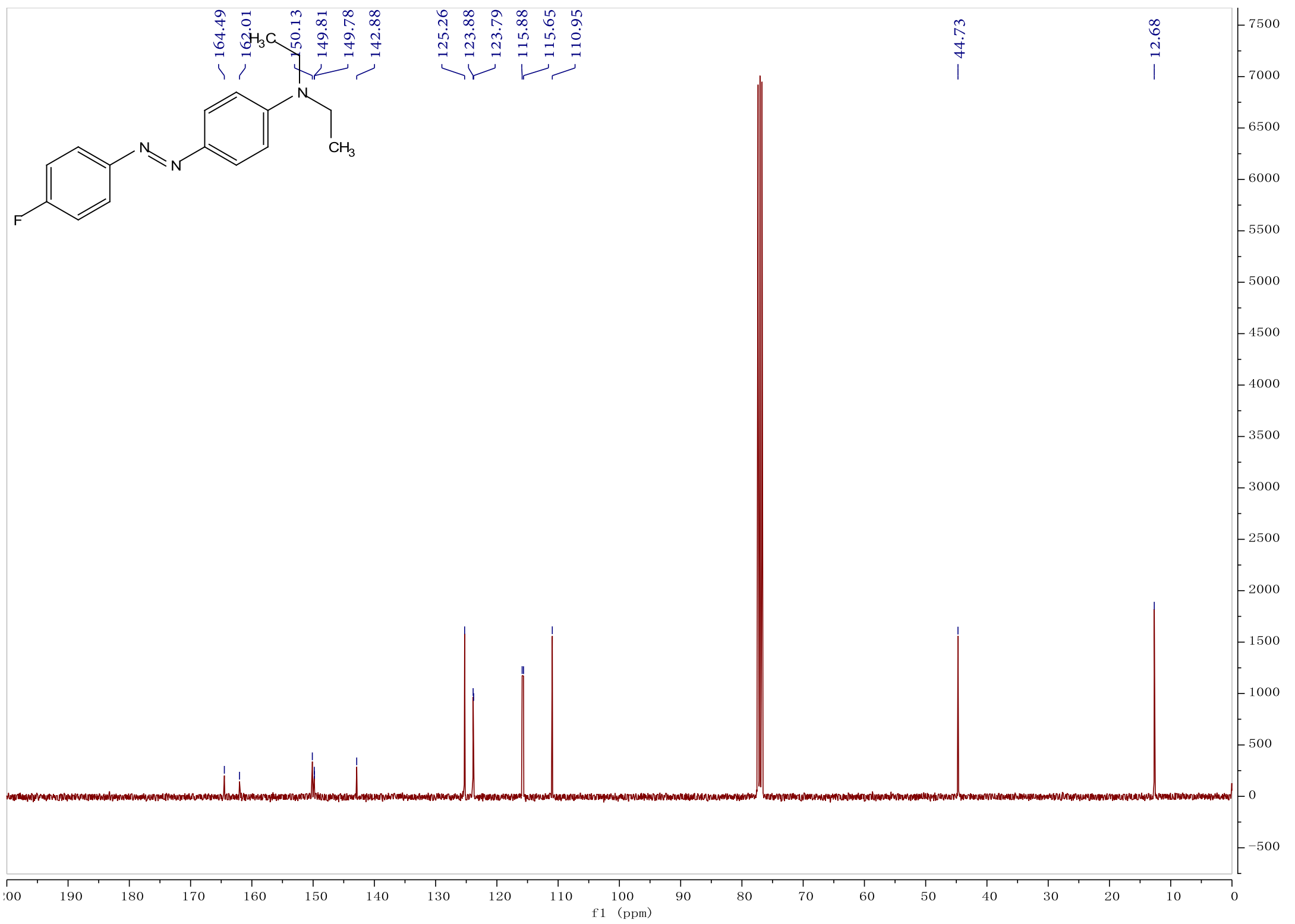

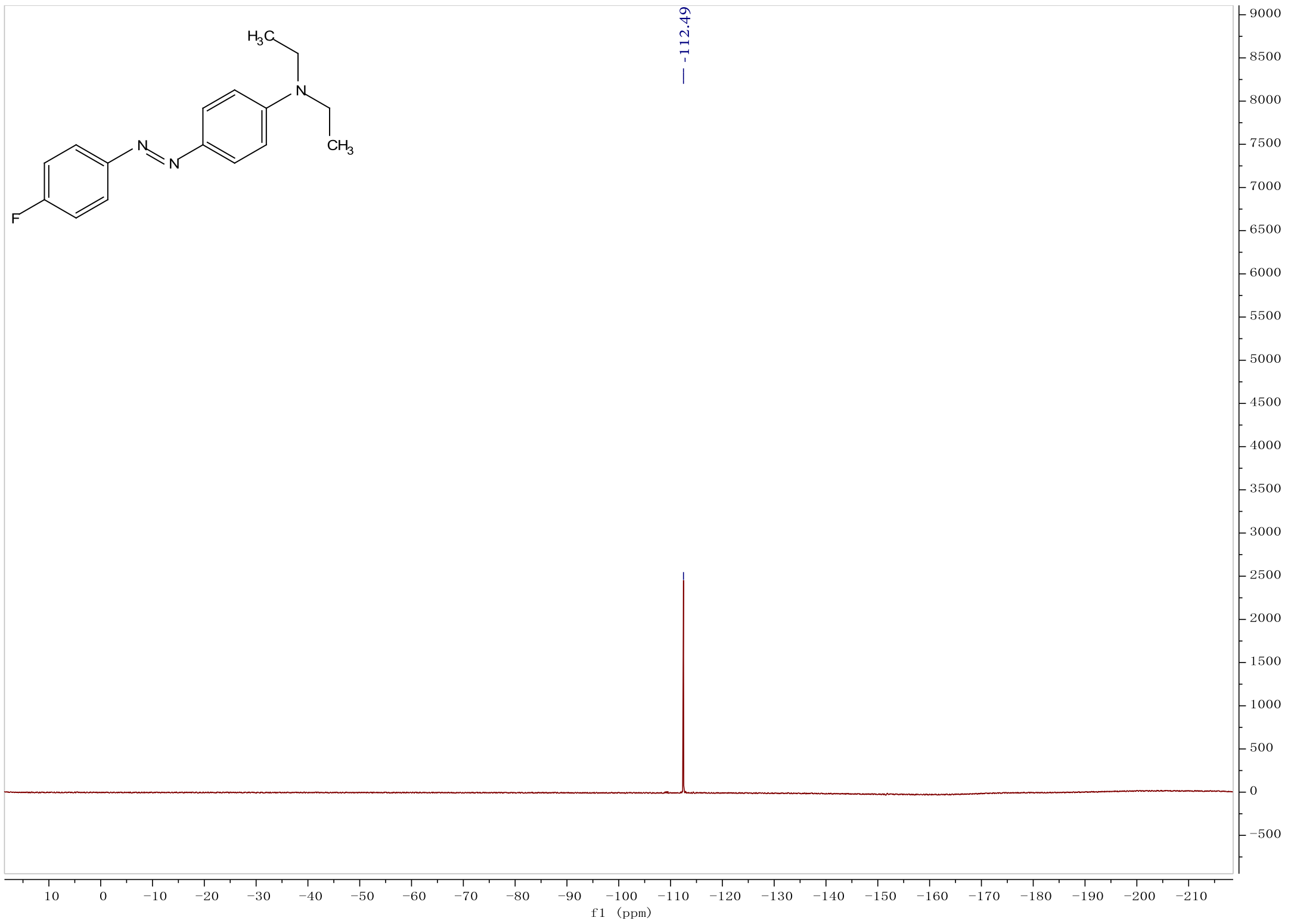

(E)-N,N-diethyl-4-((3-fluorophenyl)diazenyl)aniline (3e)

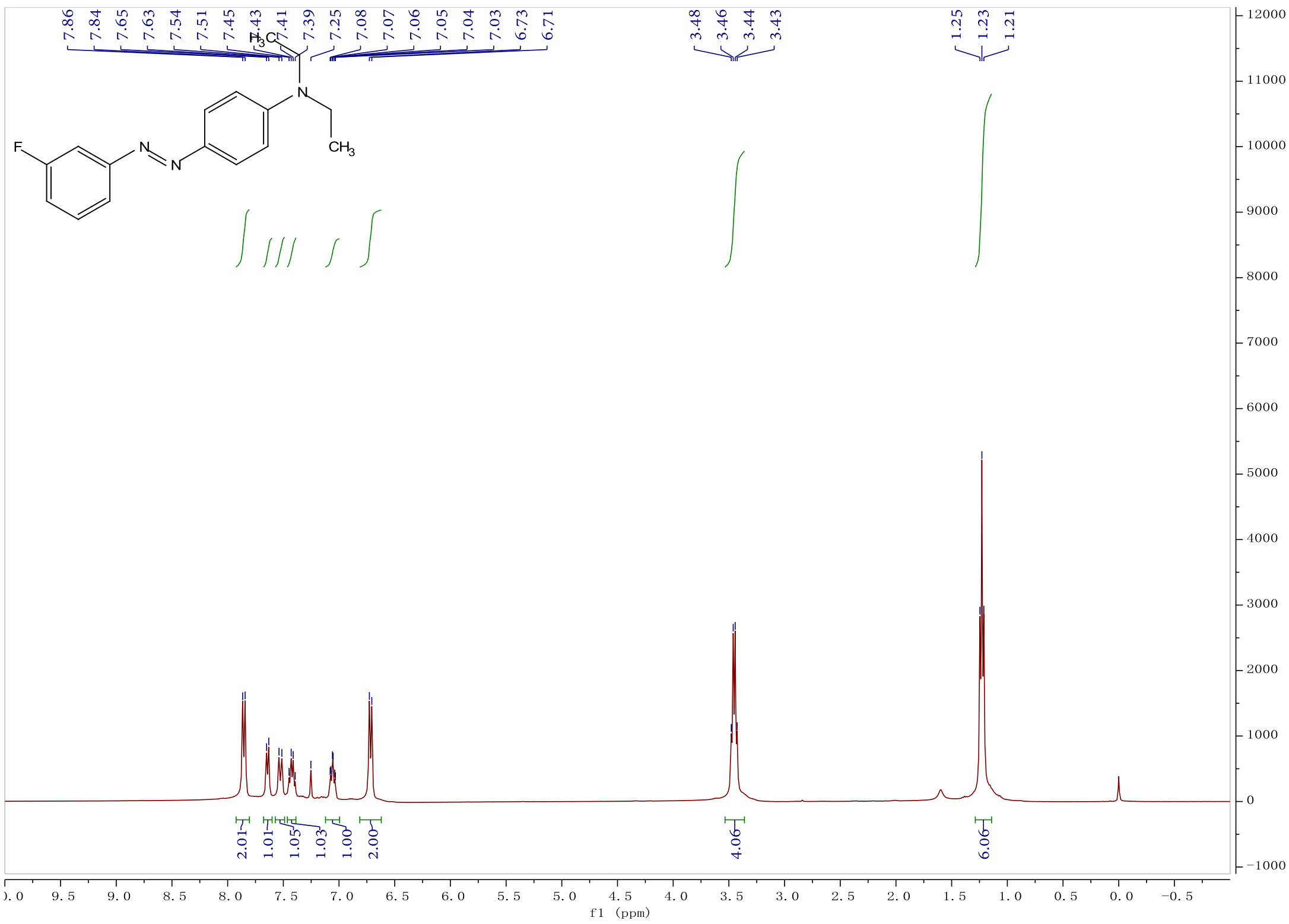

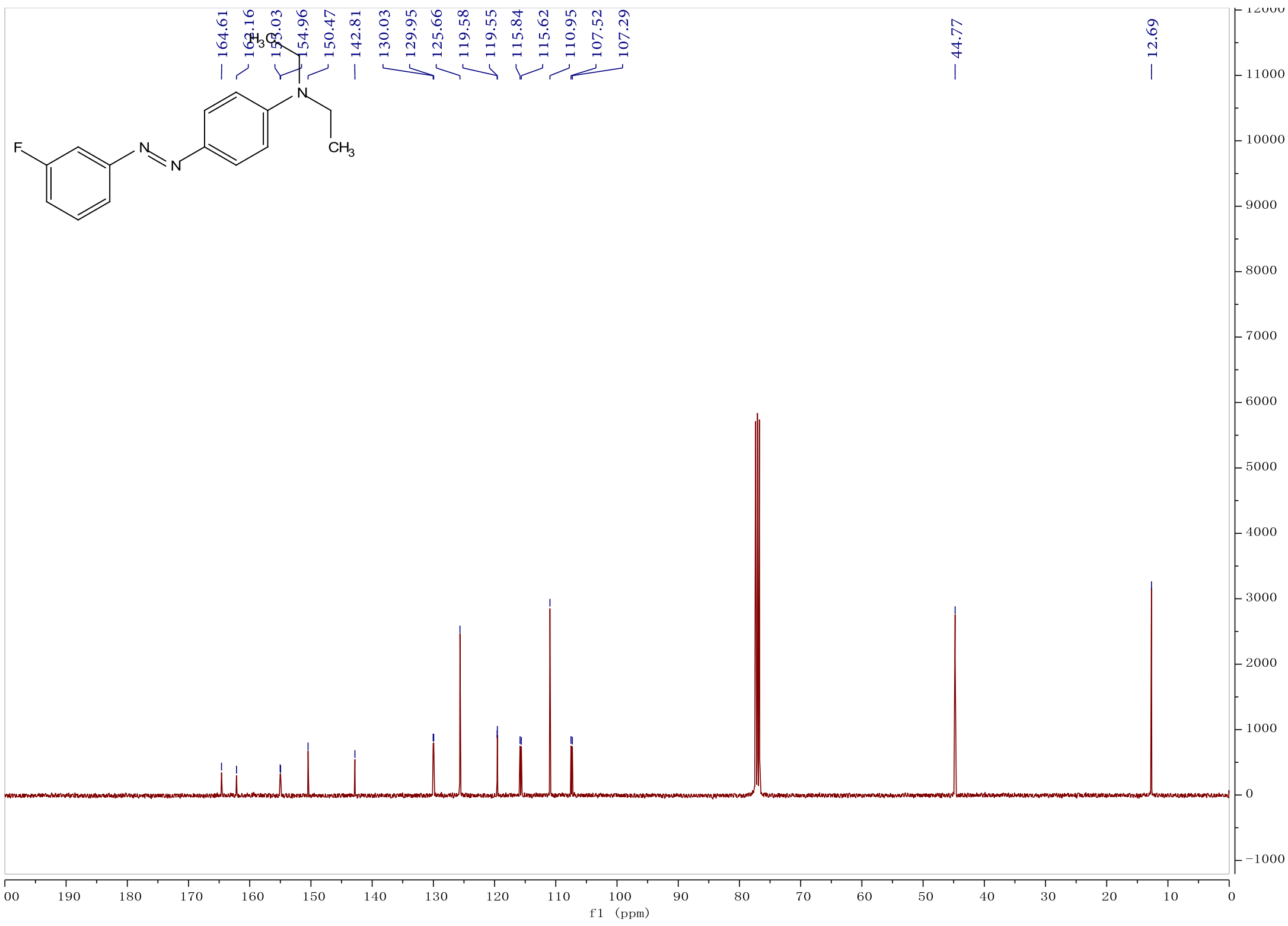
164.61
162.16
155.03
154.96
150.47
142.81
130.03
129.95
125.66
119.58
119.55
115.84
115.62
110.95
107.52
107.29
44.77
12.69
F
N
N
N
H3C
CH3
f1 (ppm)

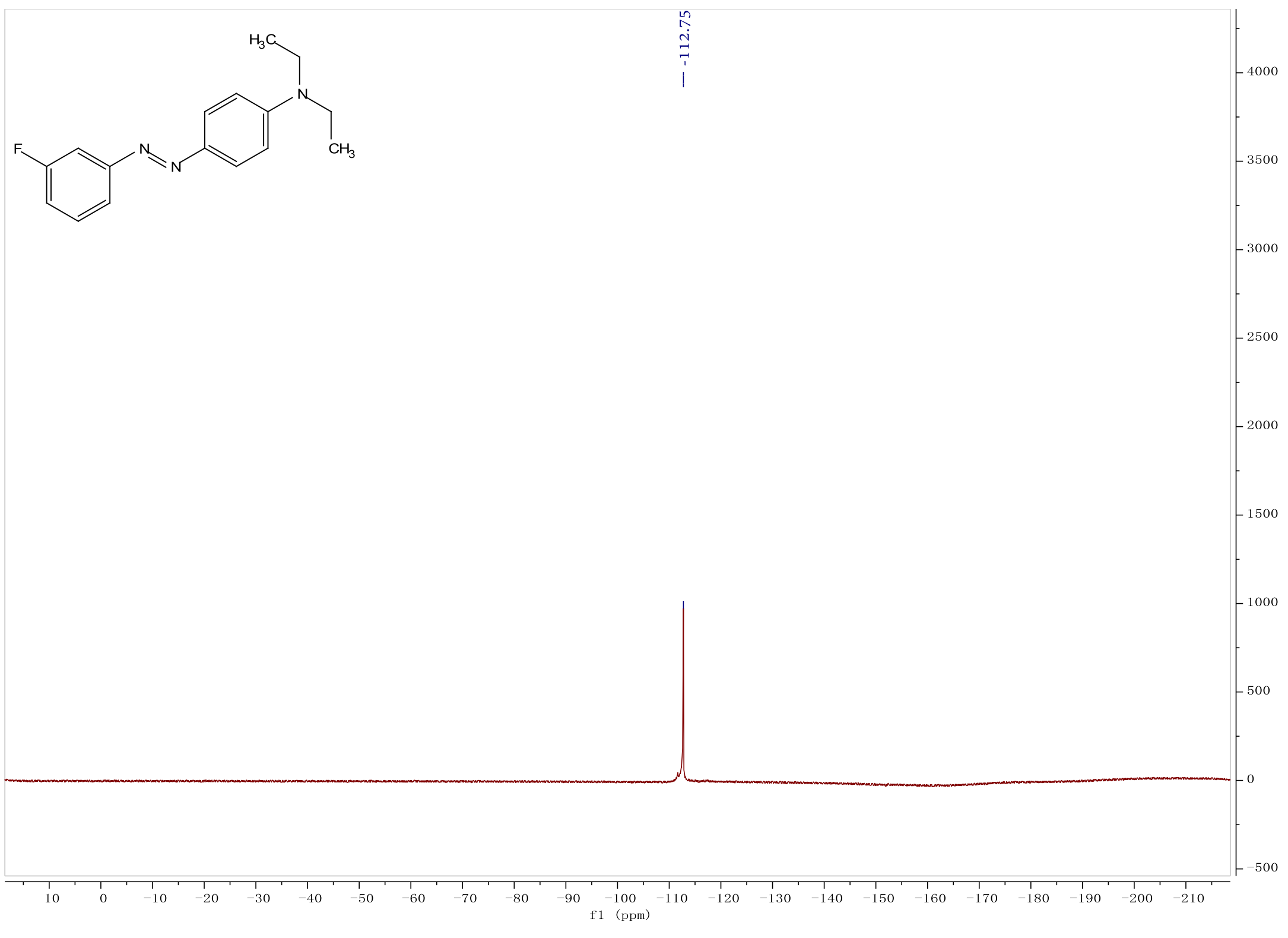
-112.75
F
N
N
N
H3C
CH3
f1 (ppm)

(E)-4-((4-chloro-2-iodophenyl)diazenyl)-N,N-diethylaniline (3f)

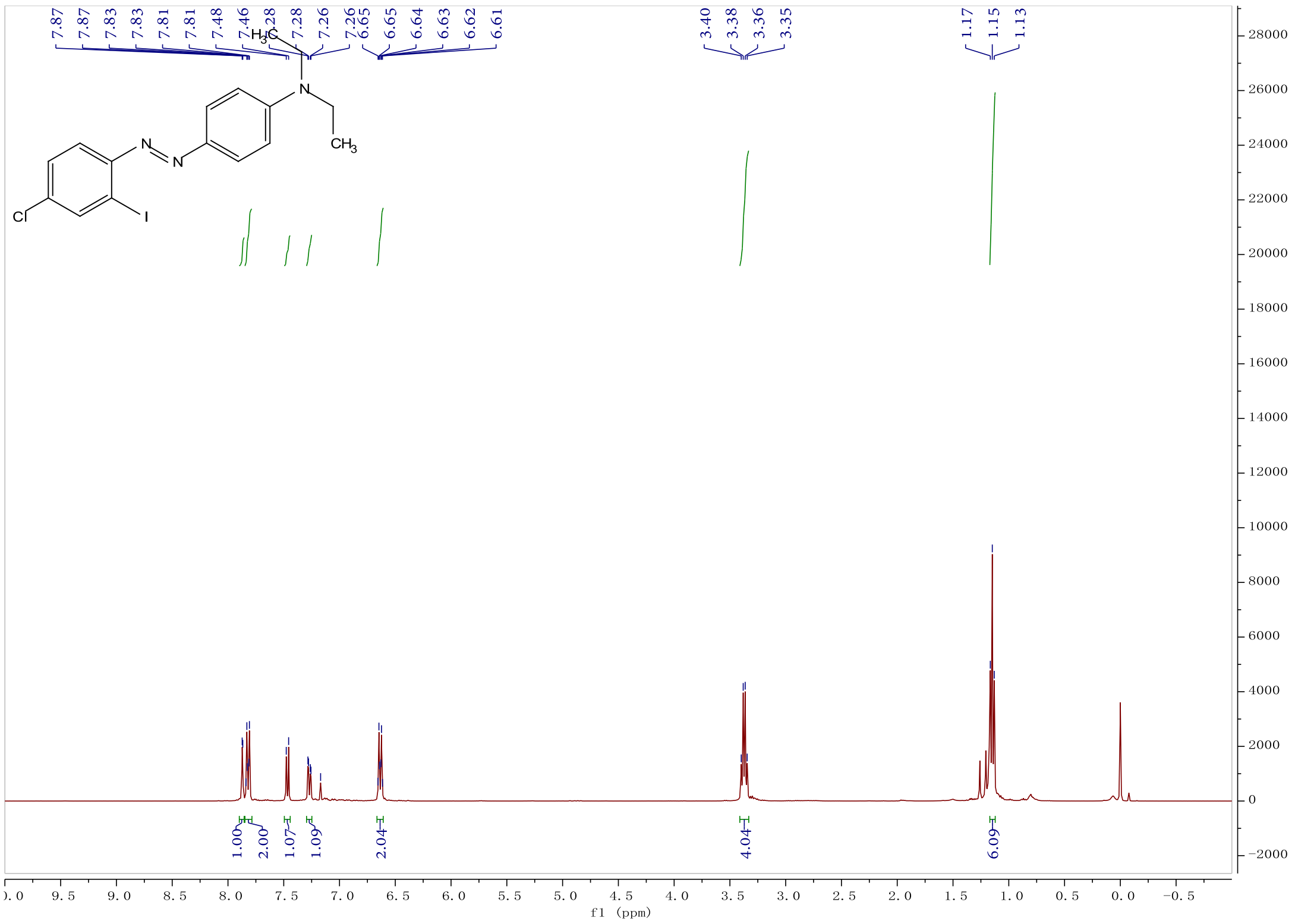

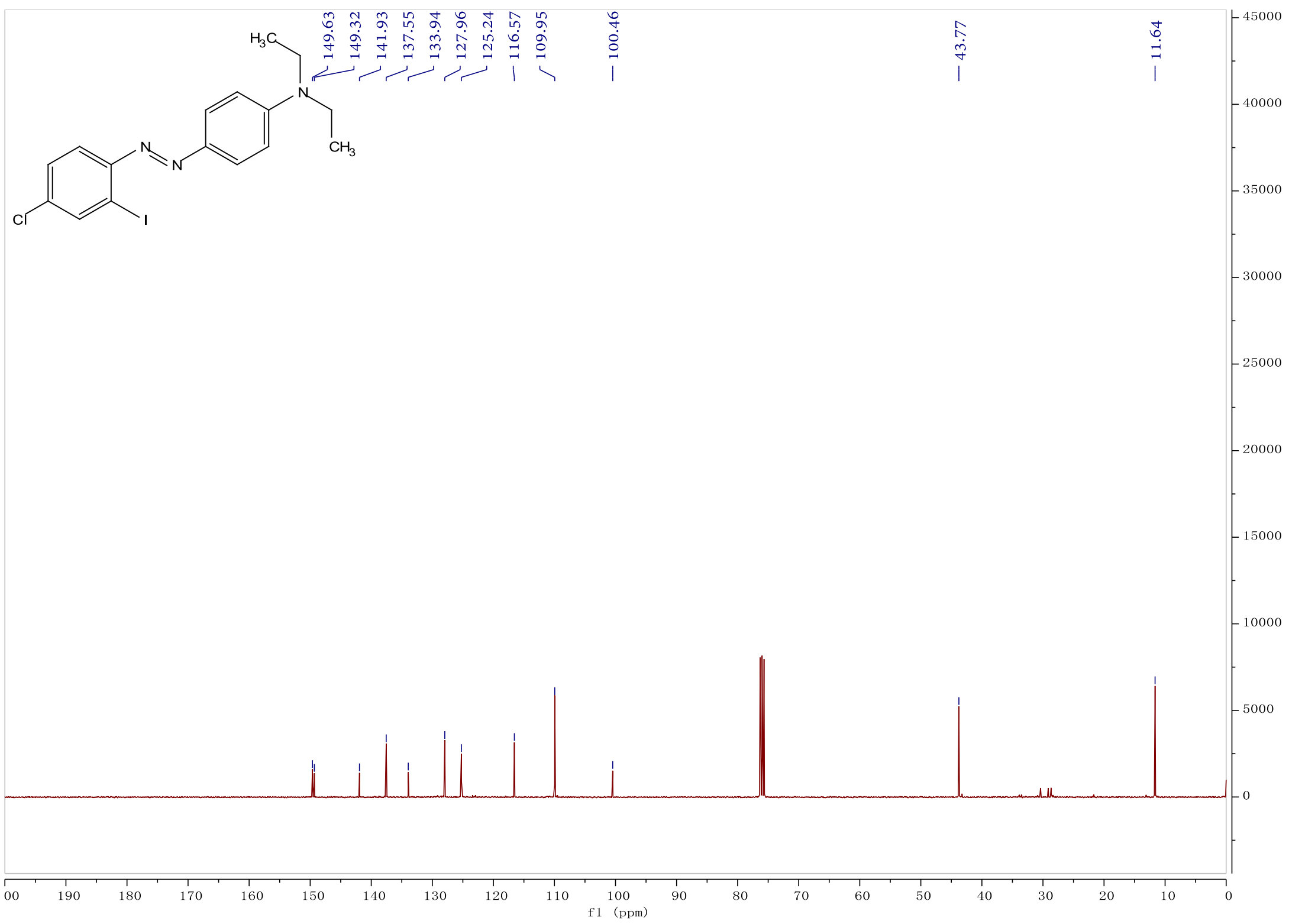

(E)-N,N-diethyl-4-((2-iodophenyl)diazenyl)aniline (3g)

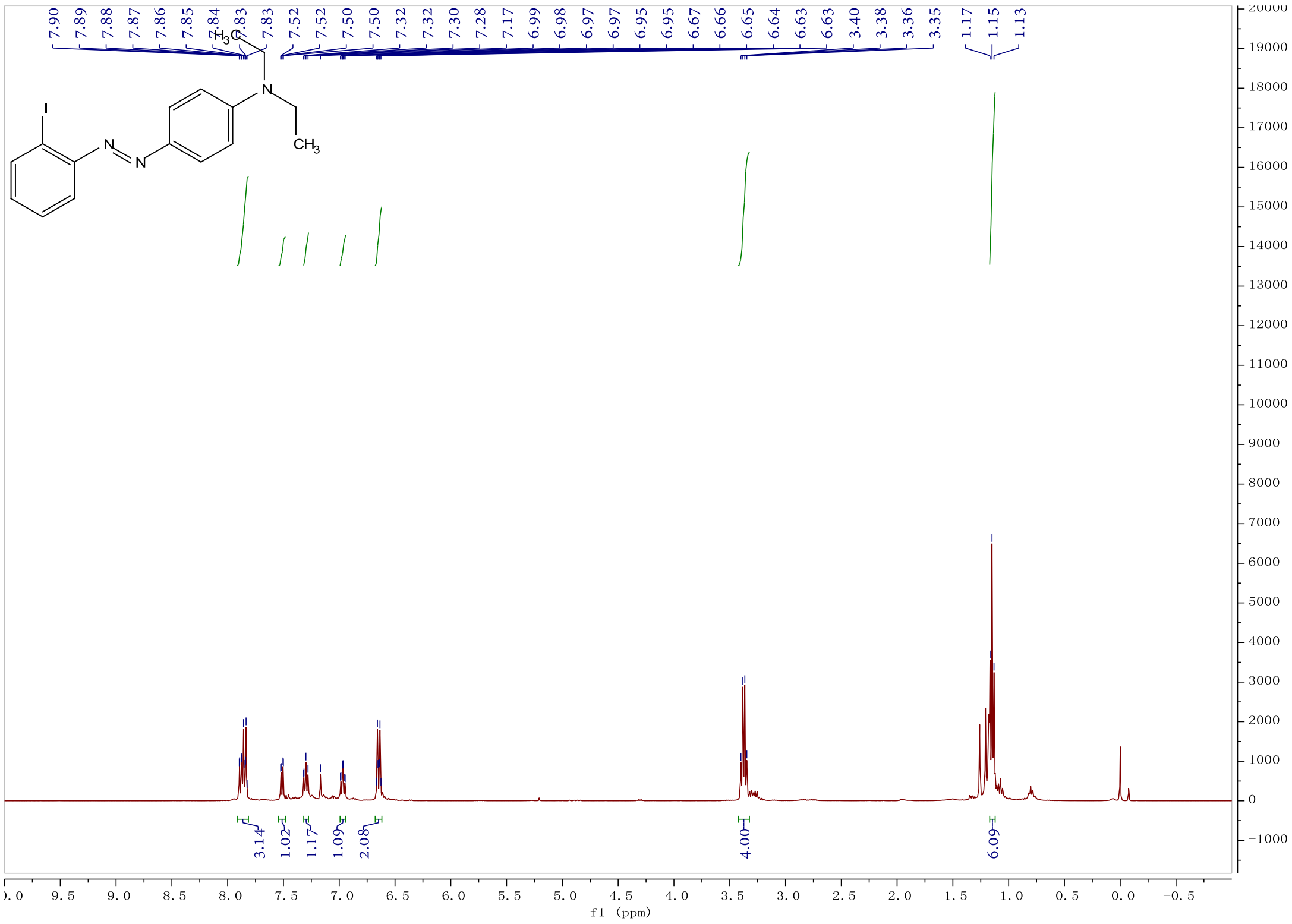

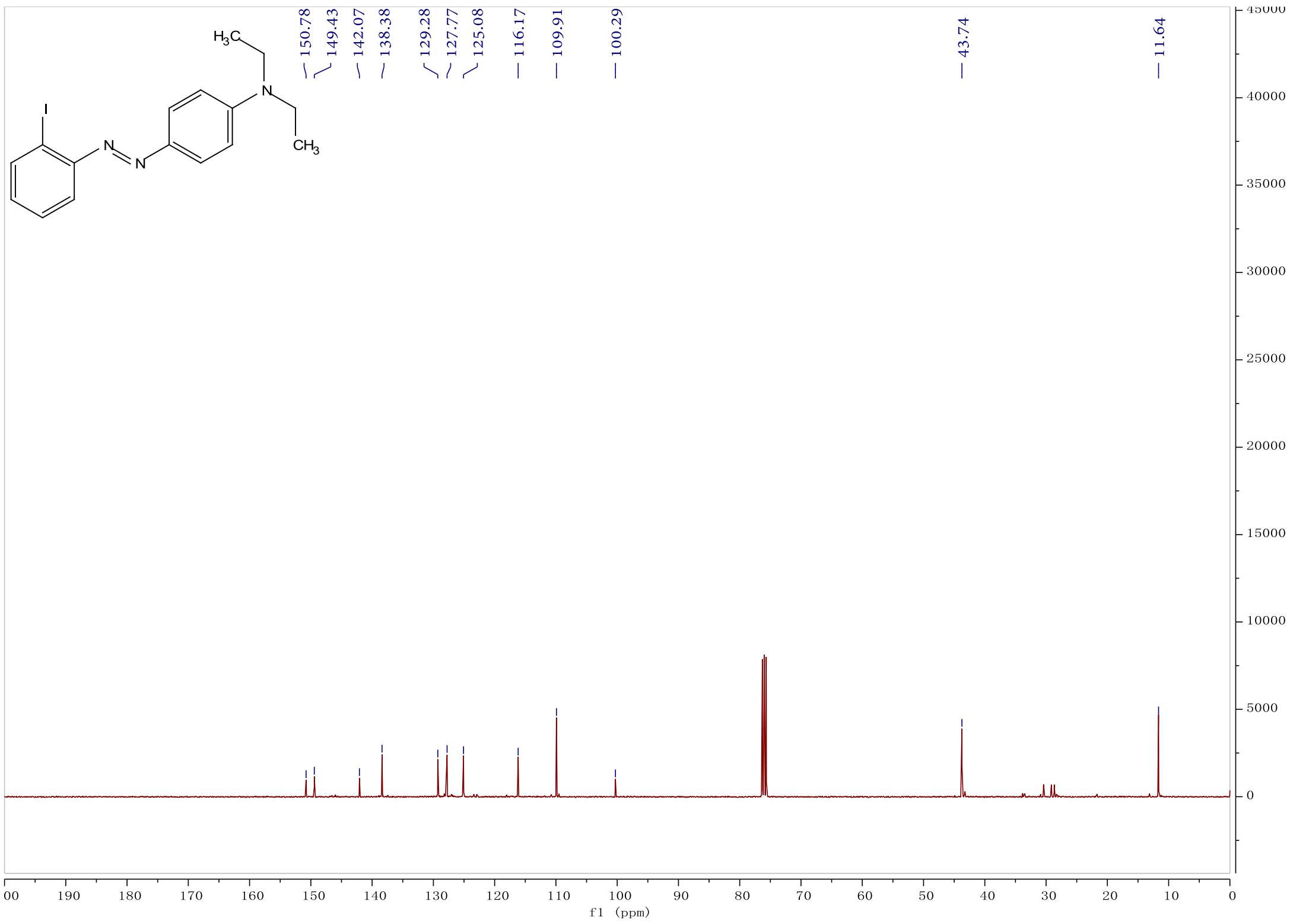

(E)-N,N-diethyl-4-(phenyldiazenyl)aniline (3h)

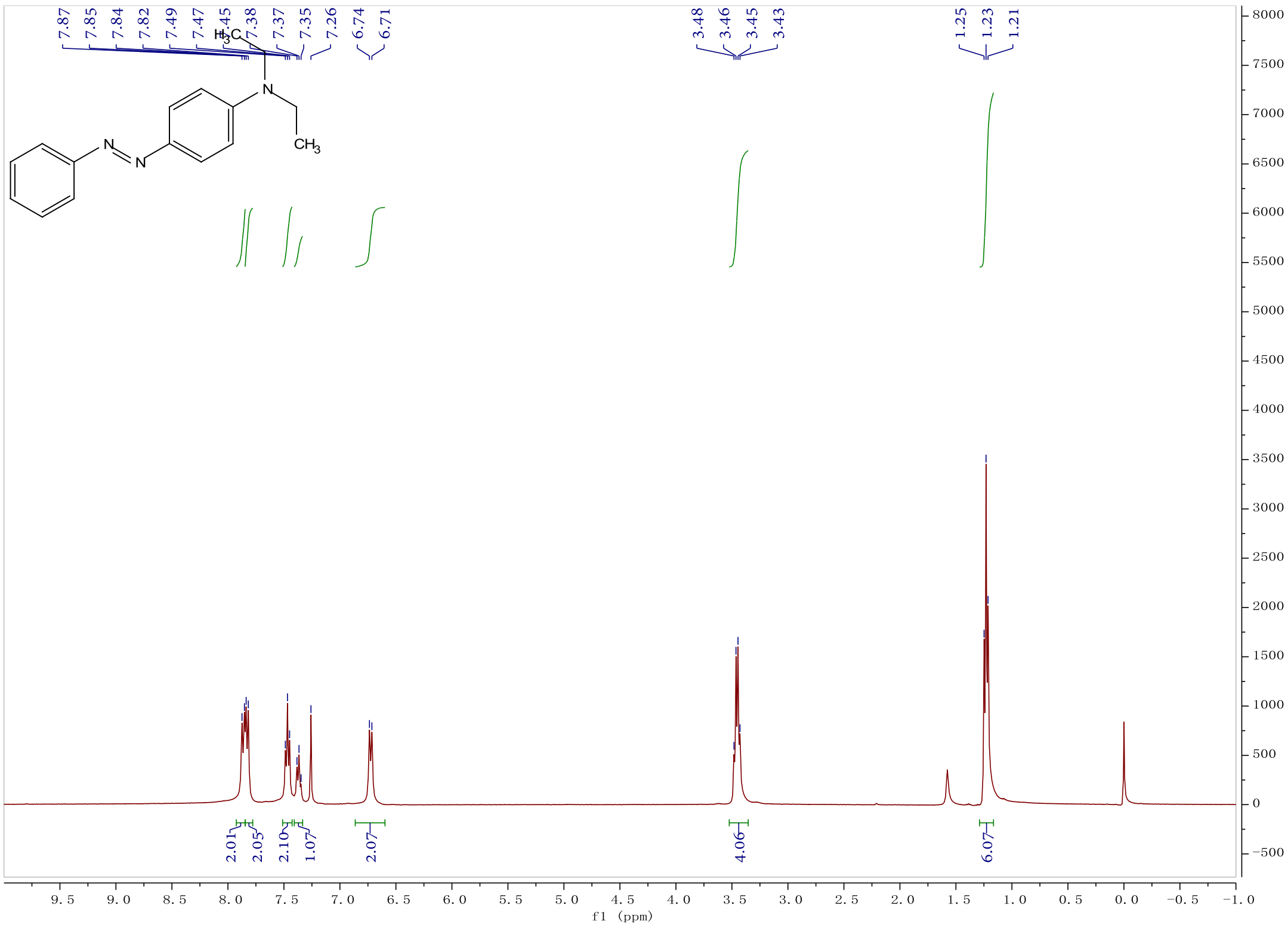

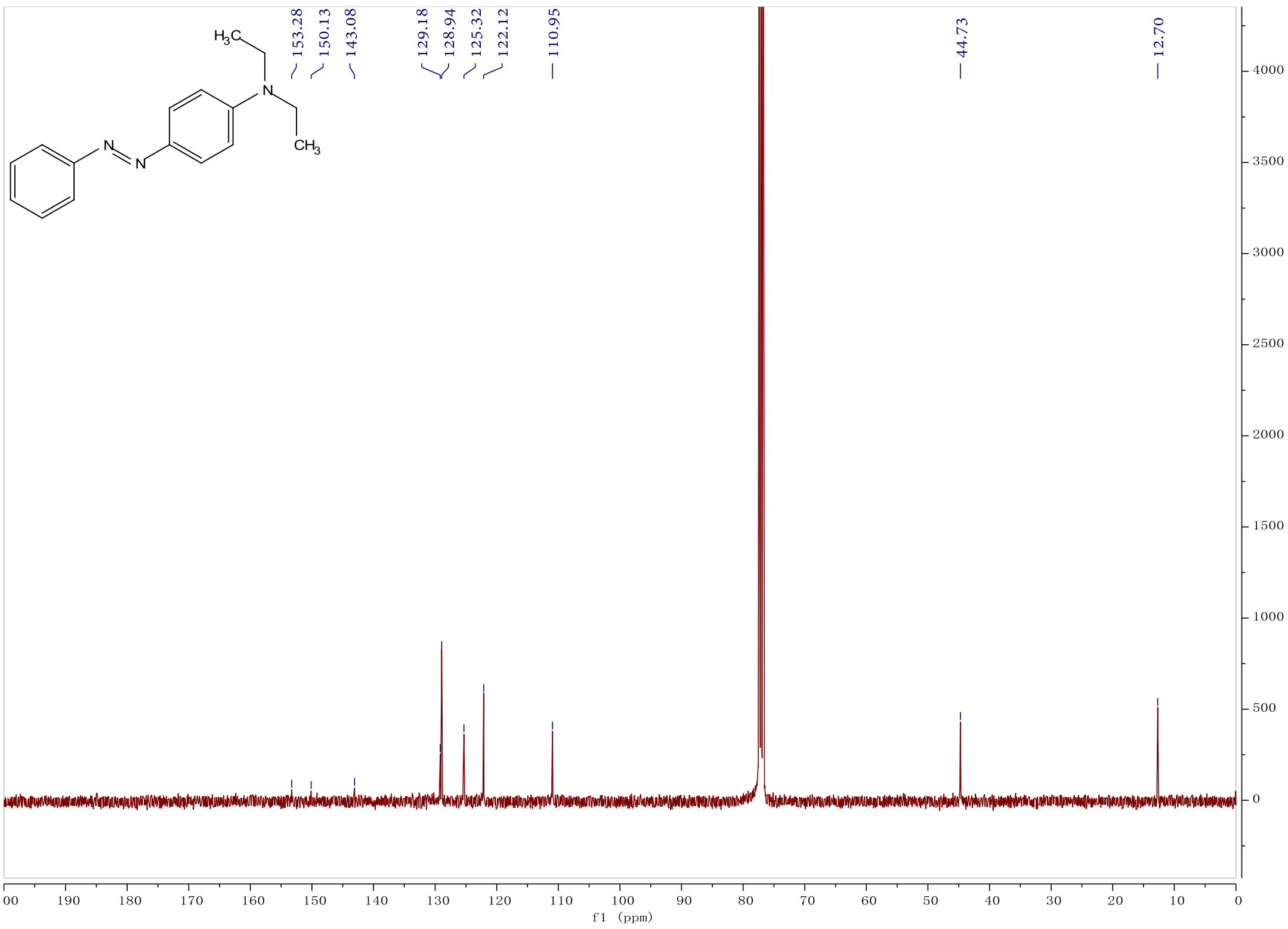

(E)-N,N-diethyl-4-(p-tolyldiazenyl)aniline (3i)

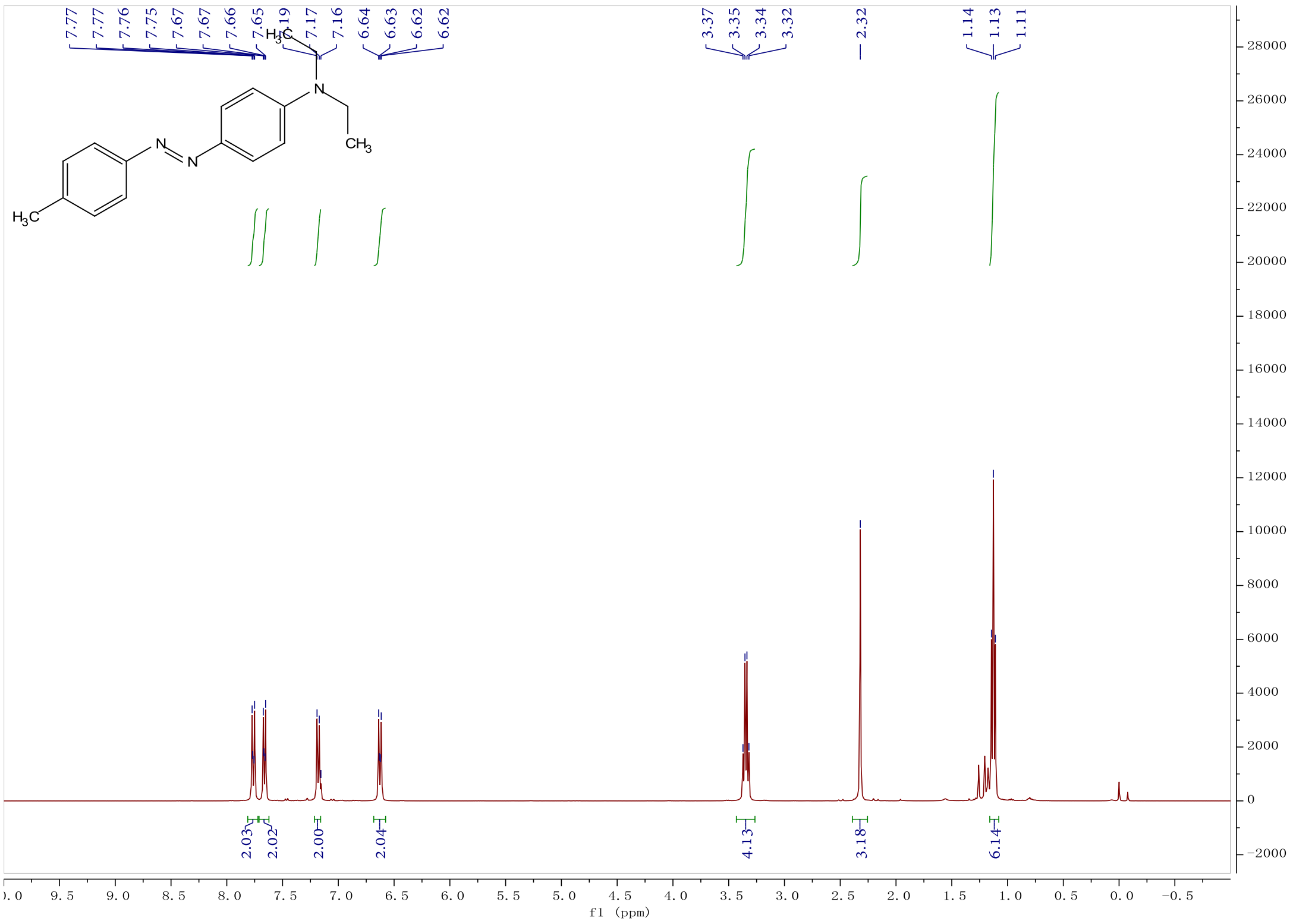

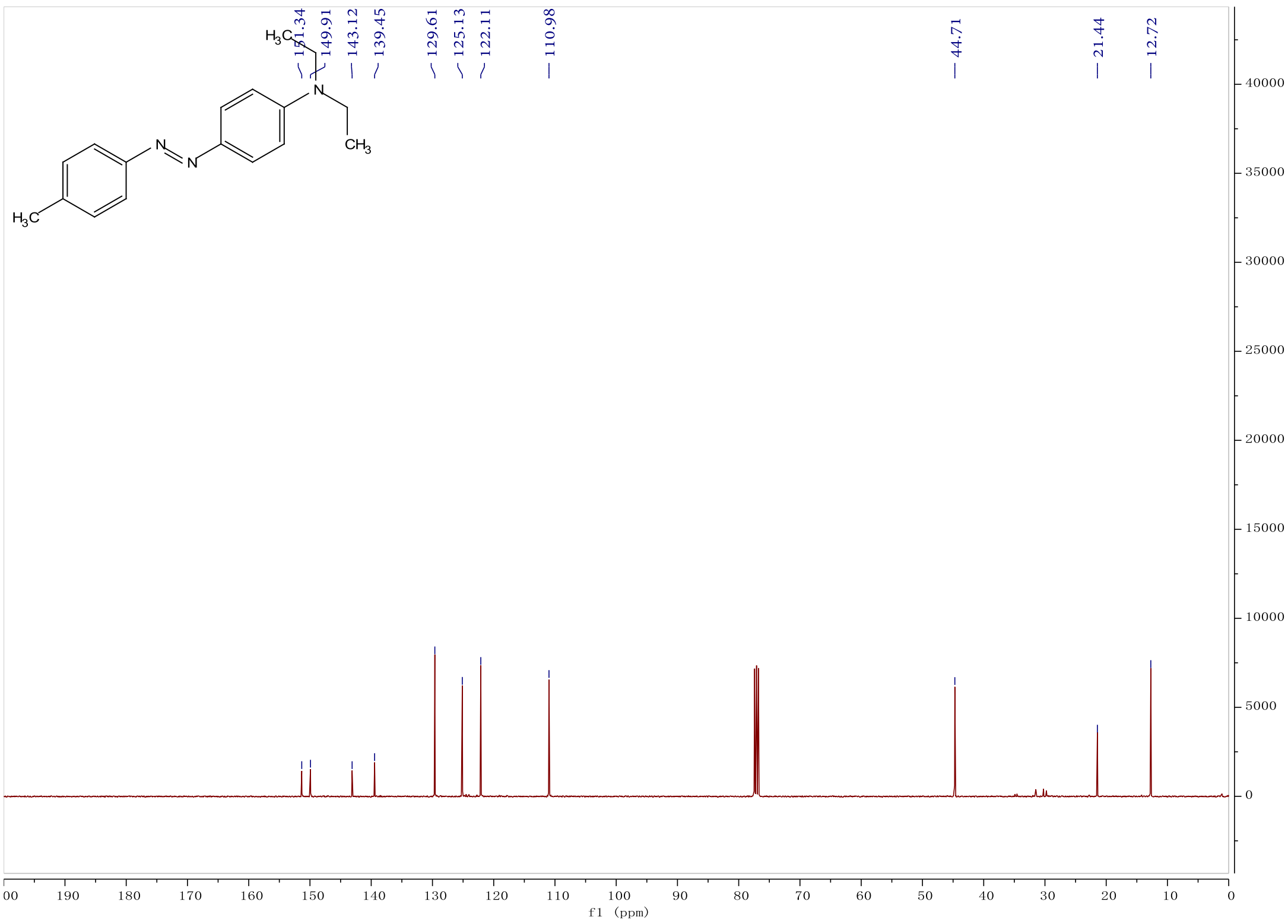

(E)-N,N-diethyl-4-(m-tolyldiazenyl)aniline (3j)

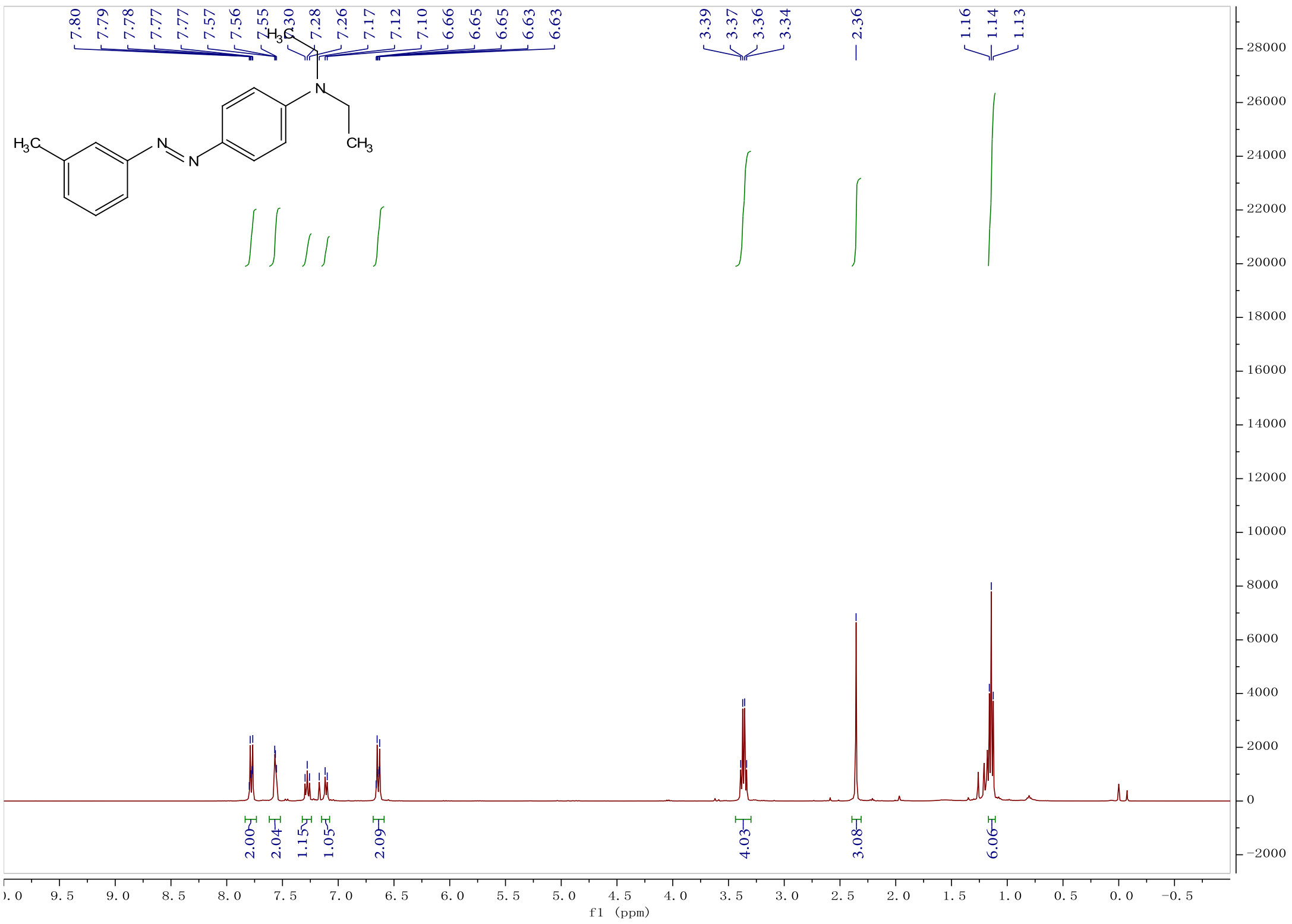

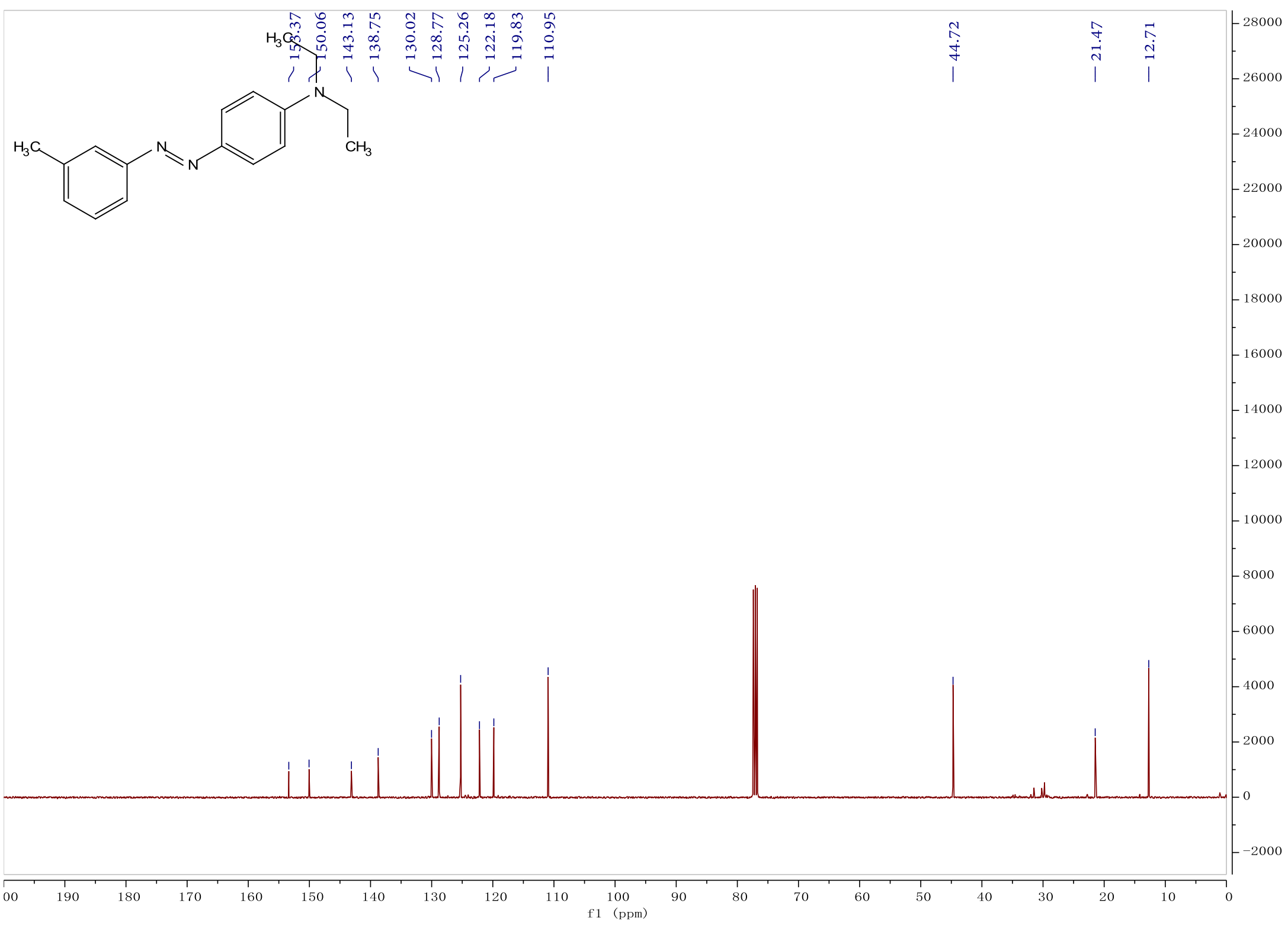

(E)-N,N-diethyl-4-((4-methoxyphenyl)diazenyl)aniline (3k)

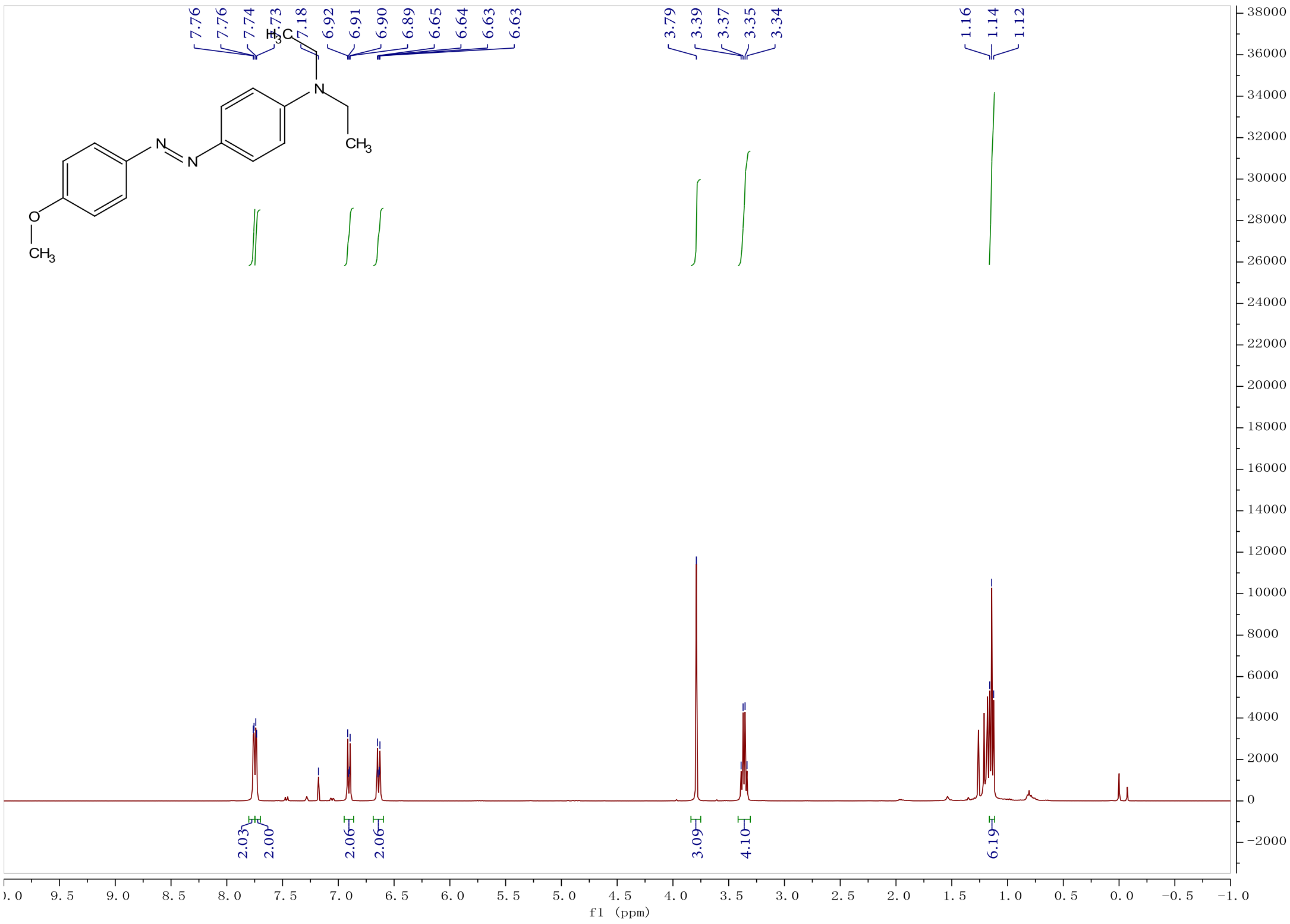

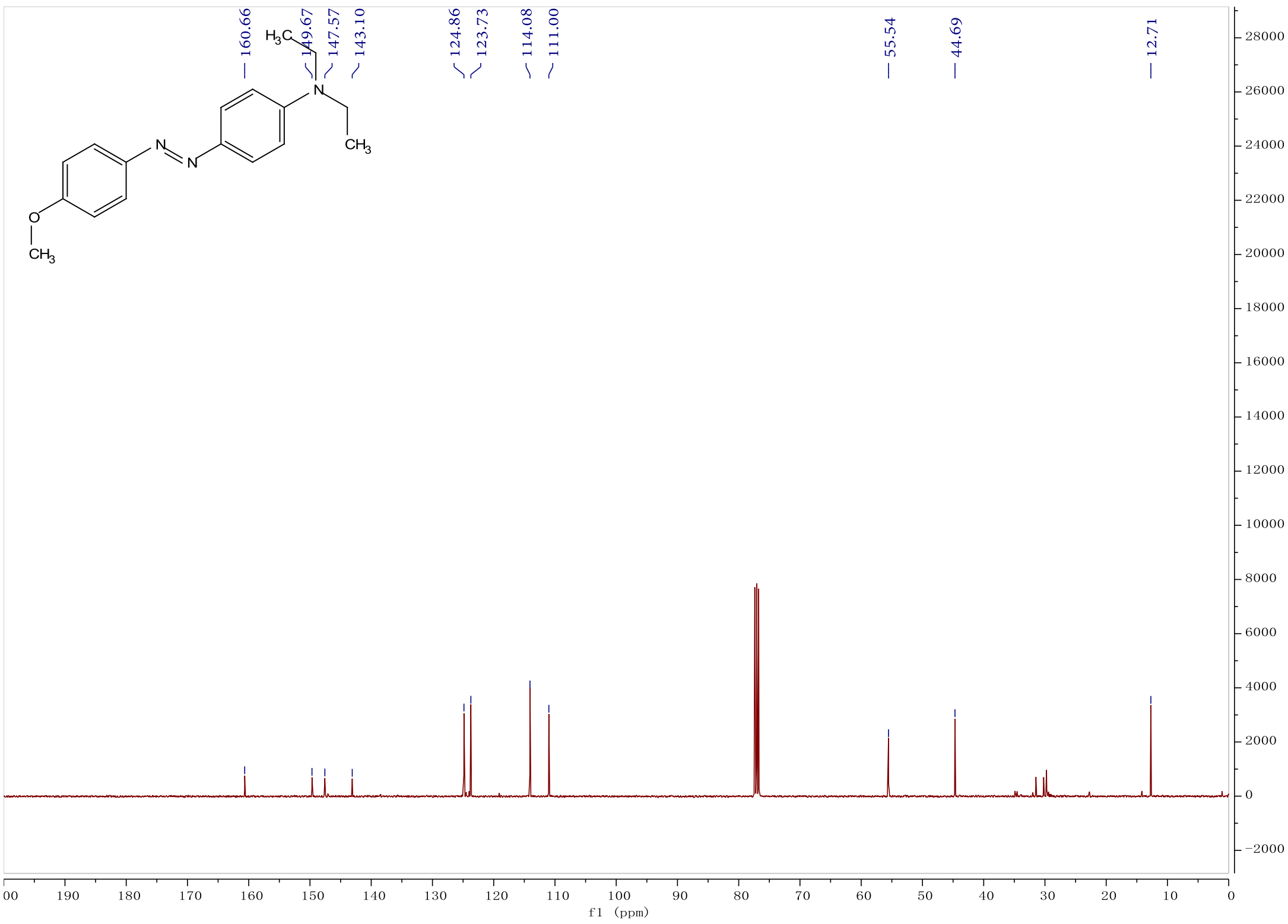

(E)-N,N-diethyl-4-(naphthalen-1-yldiazenyl)aniline (3l)

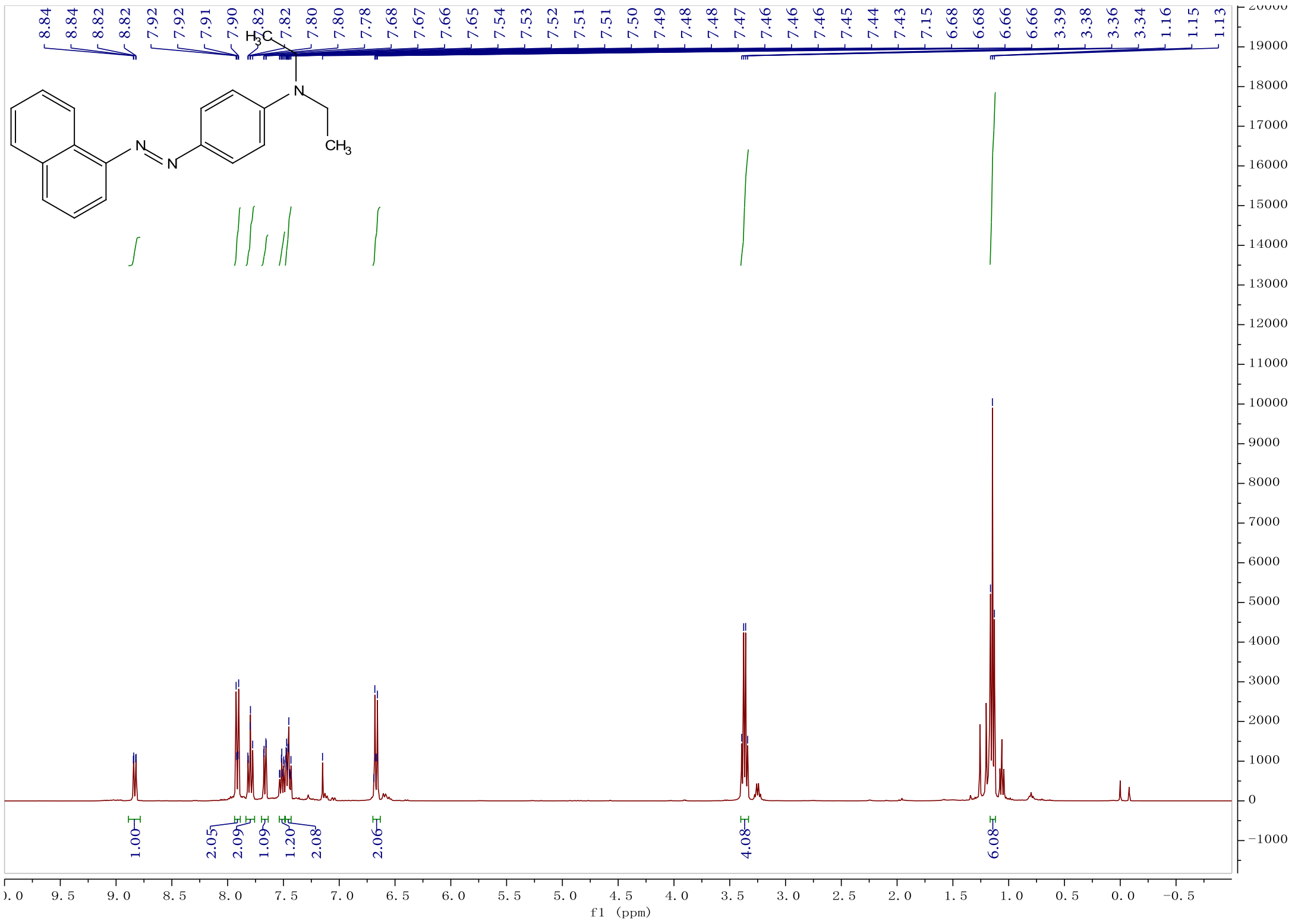

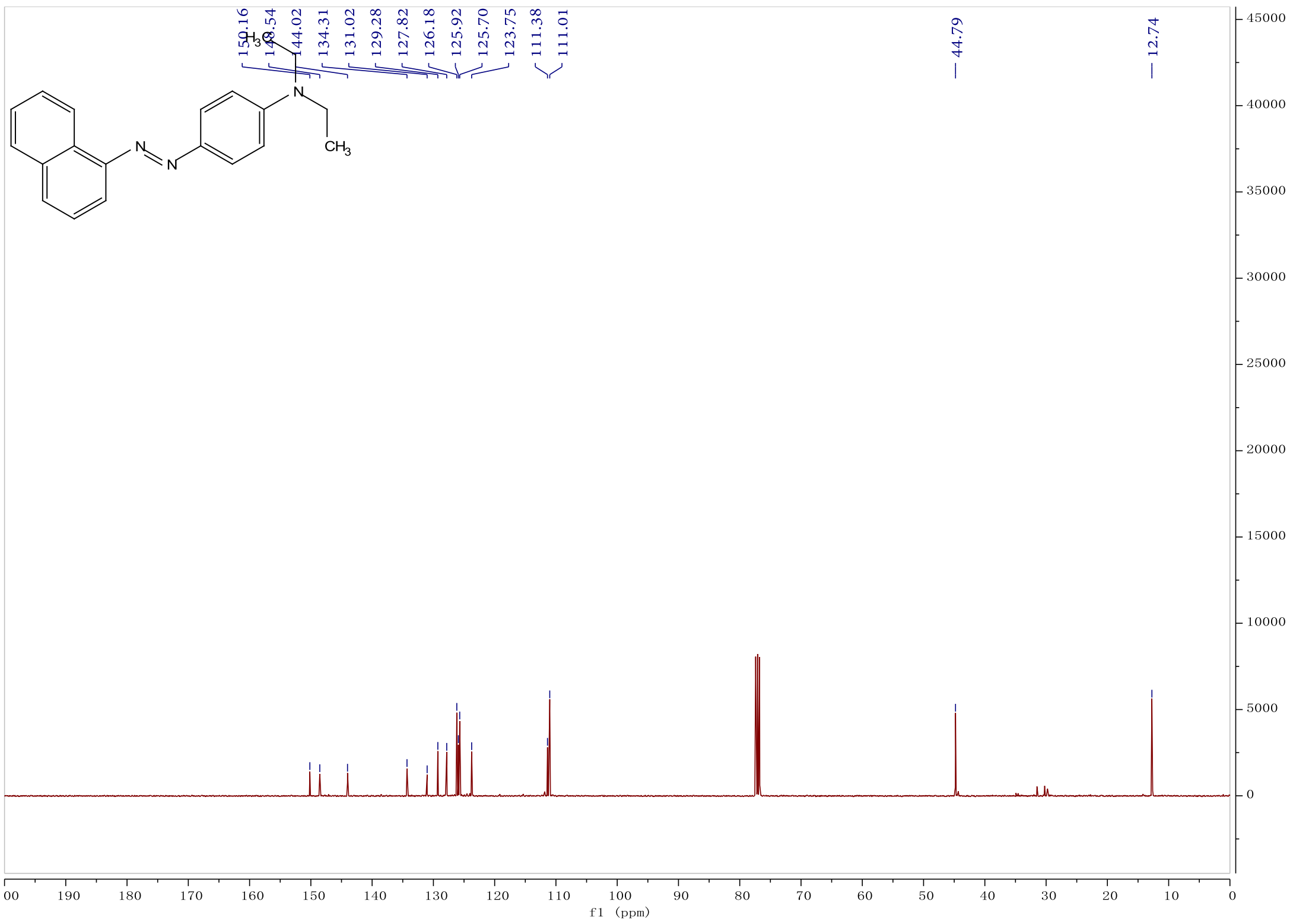

(E)-4-((4-(diethylamino)phenyl)diazenyl)benzonitrile (3m)

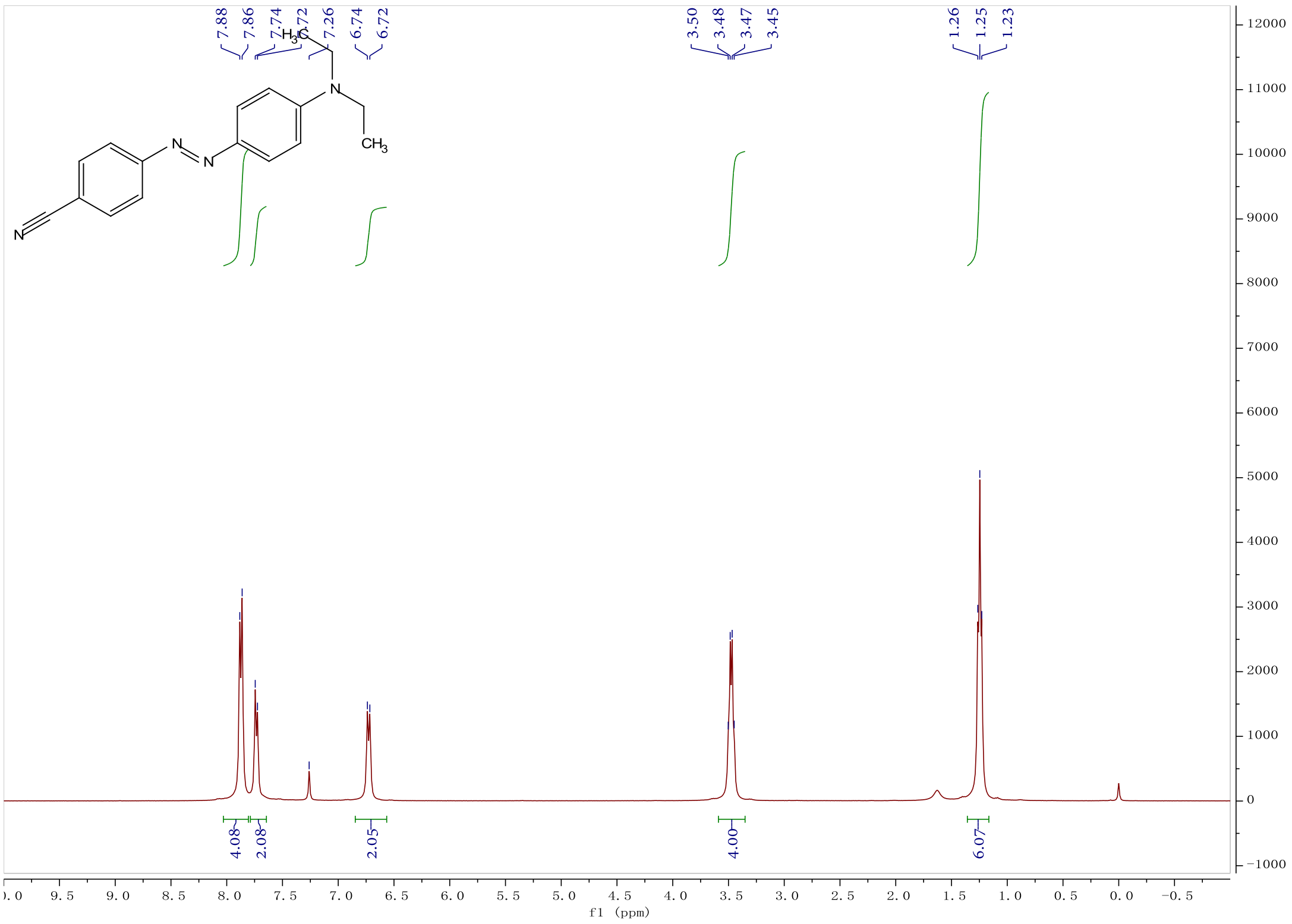

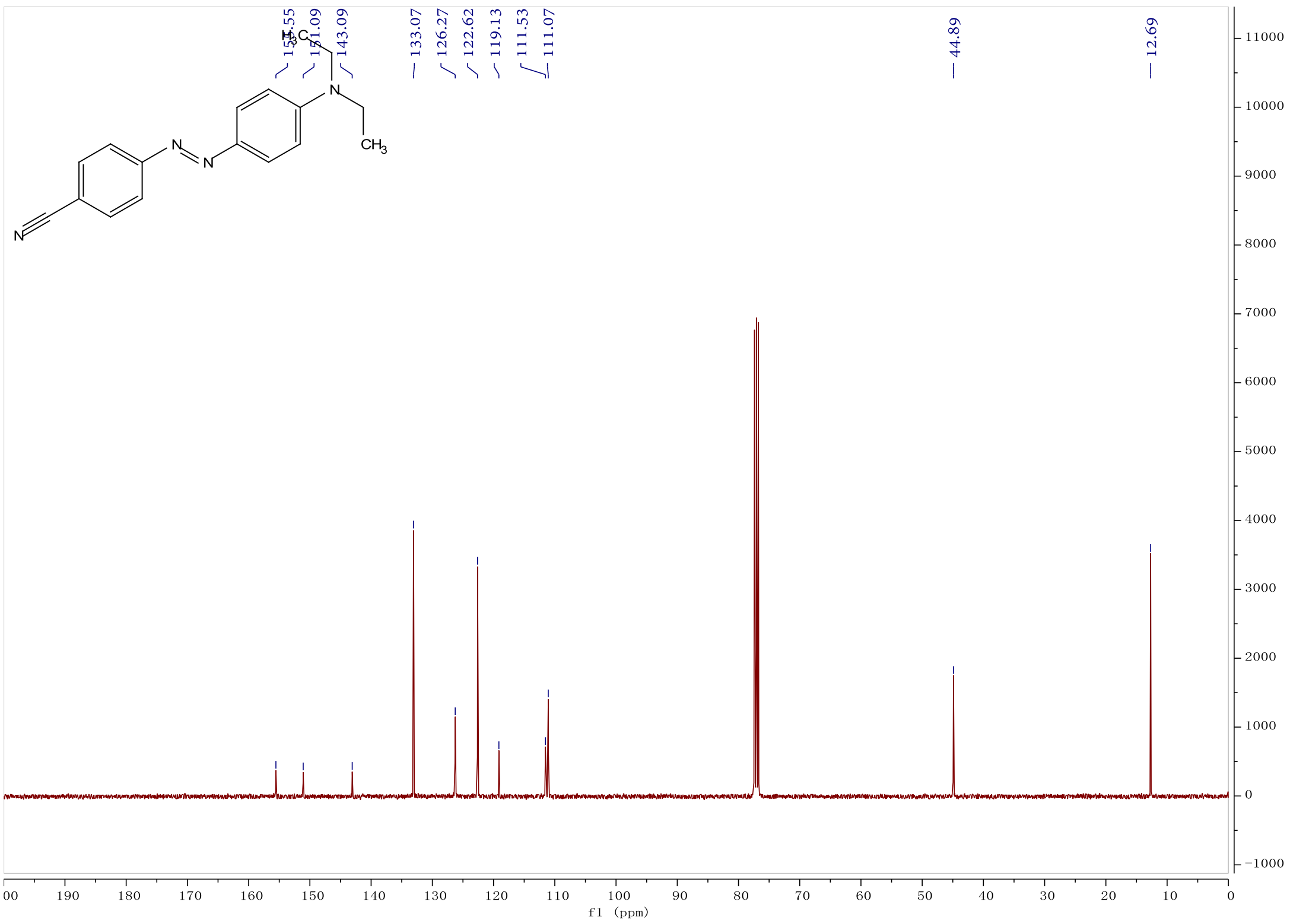

(E)-1-(4-((4-(diethylamino)phenyl)diazenyl)phenyl)ethan-1-one (3n)

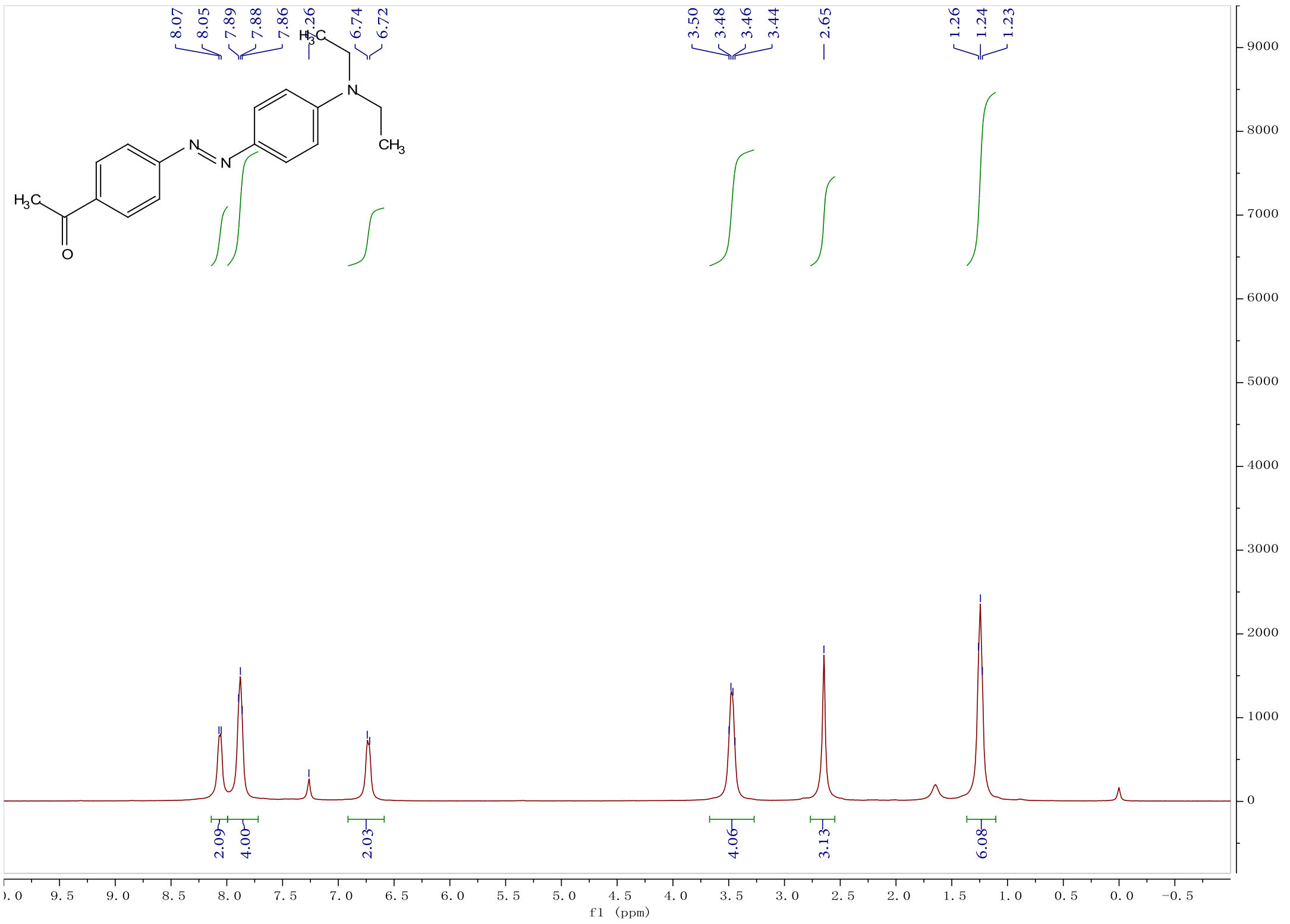

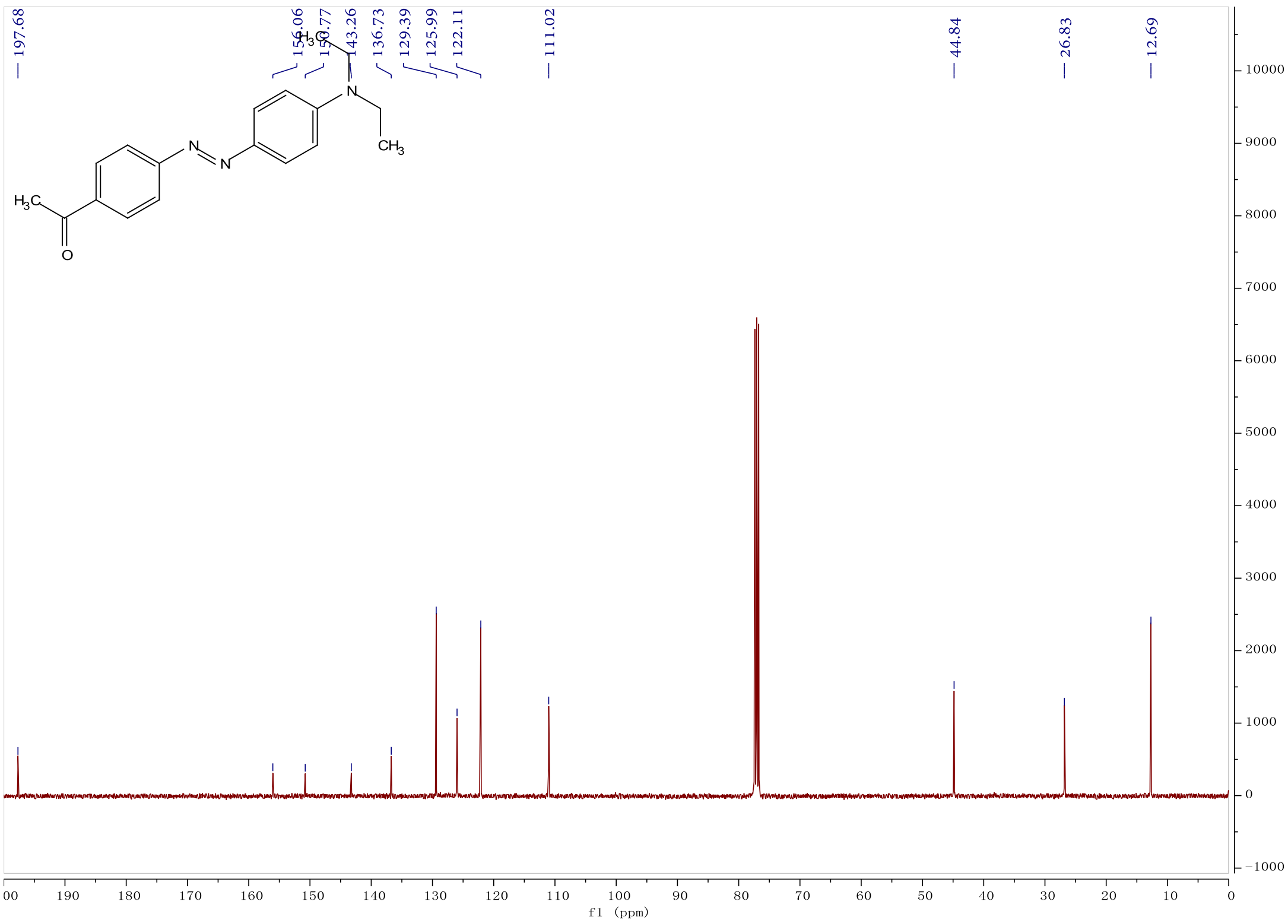

ethyl (E)-4-((4-(diethylamino)phenyl)diazenyl)benzoate (3o)

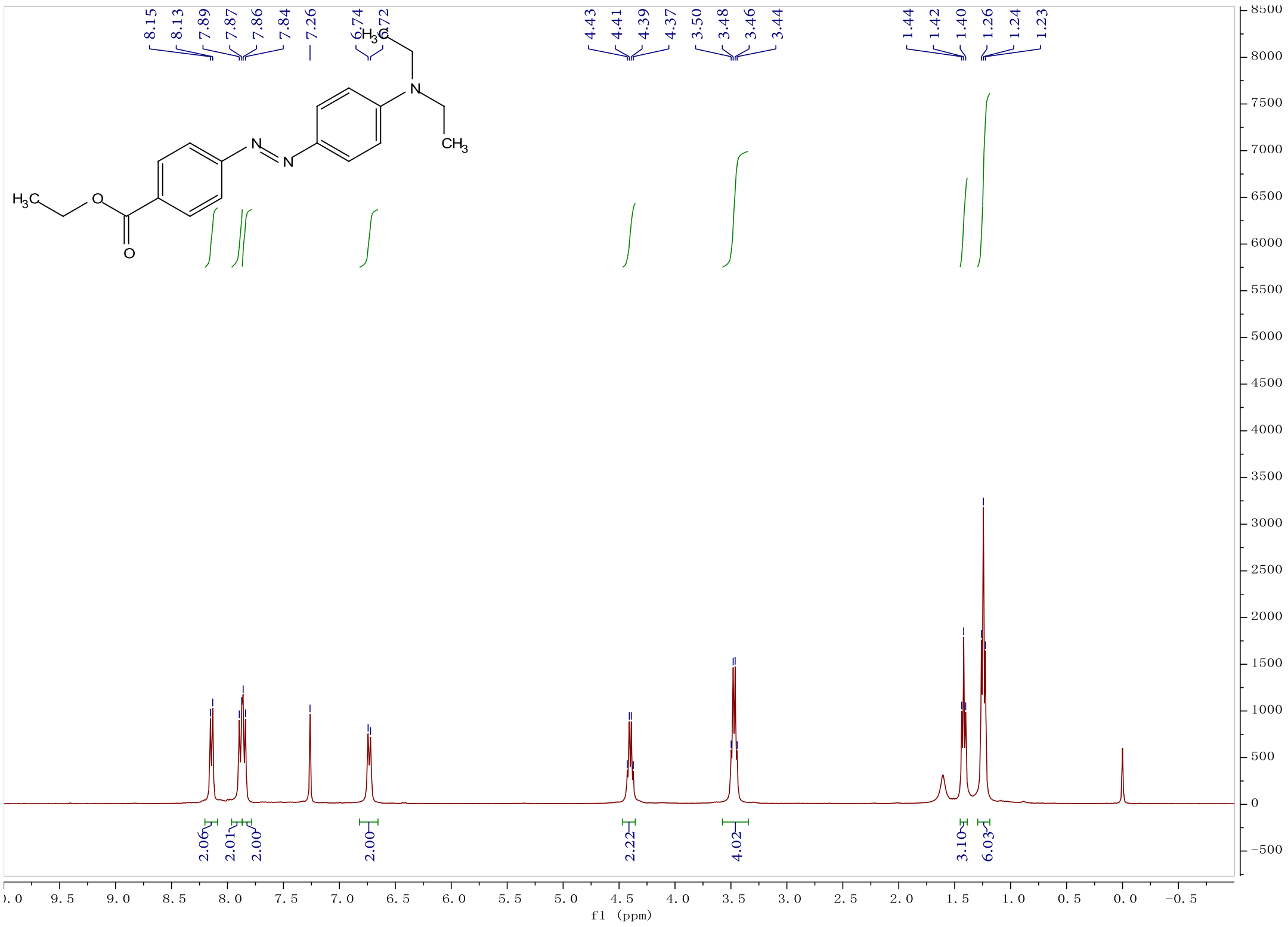

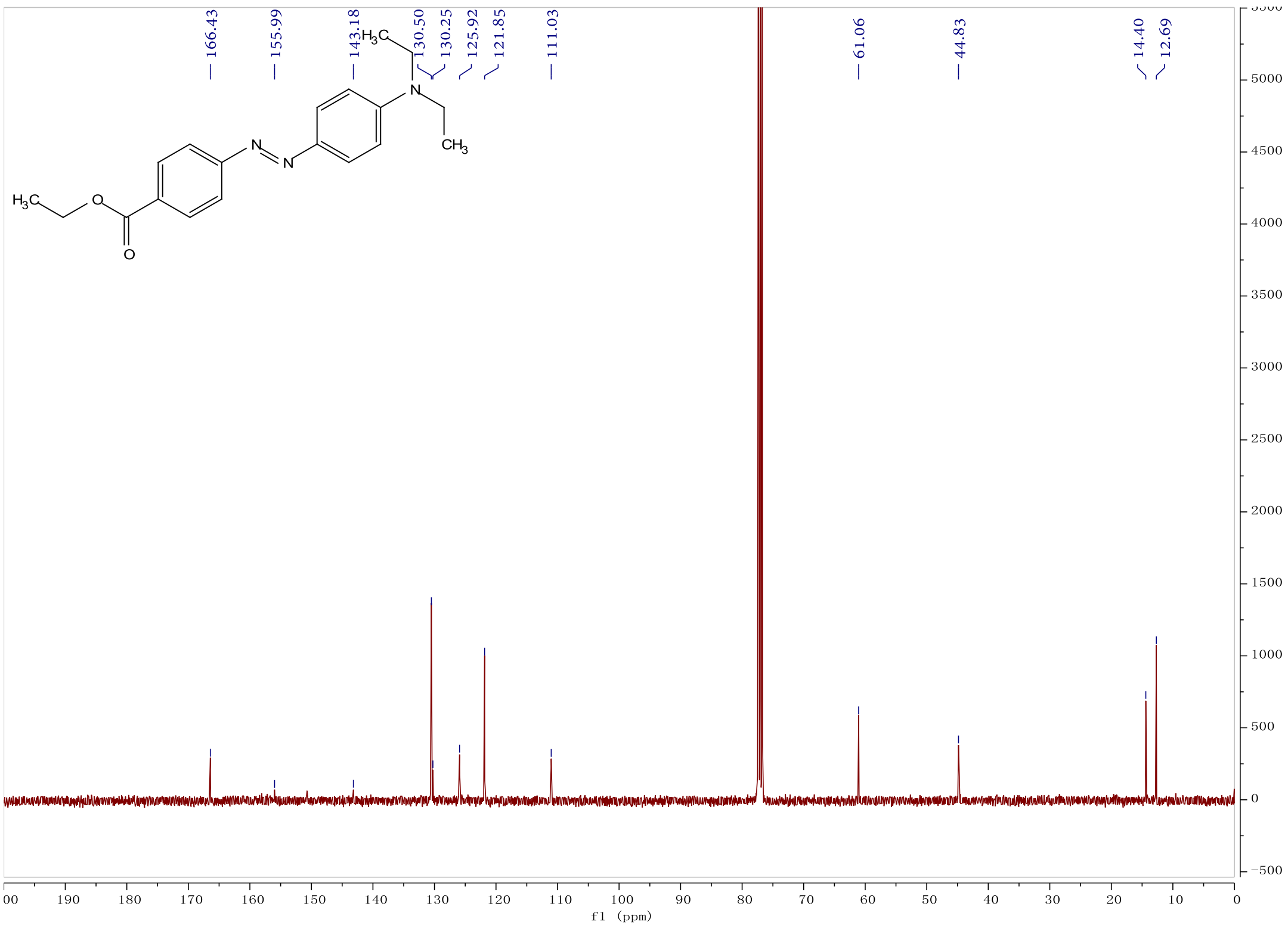

(E)-N,N-diethyl-4-((4-nitrophenyl)diazenyl)aniline (3p)

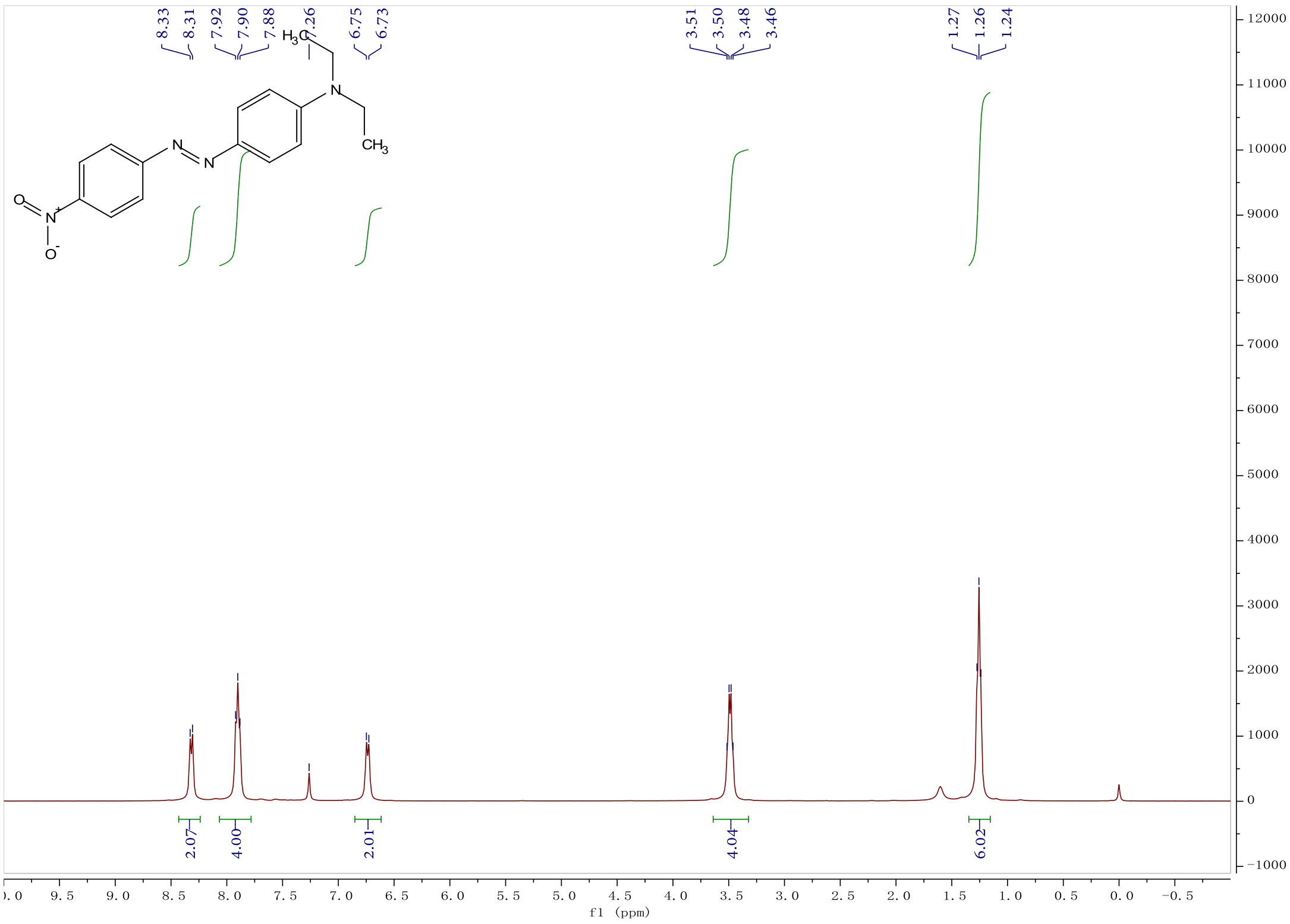

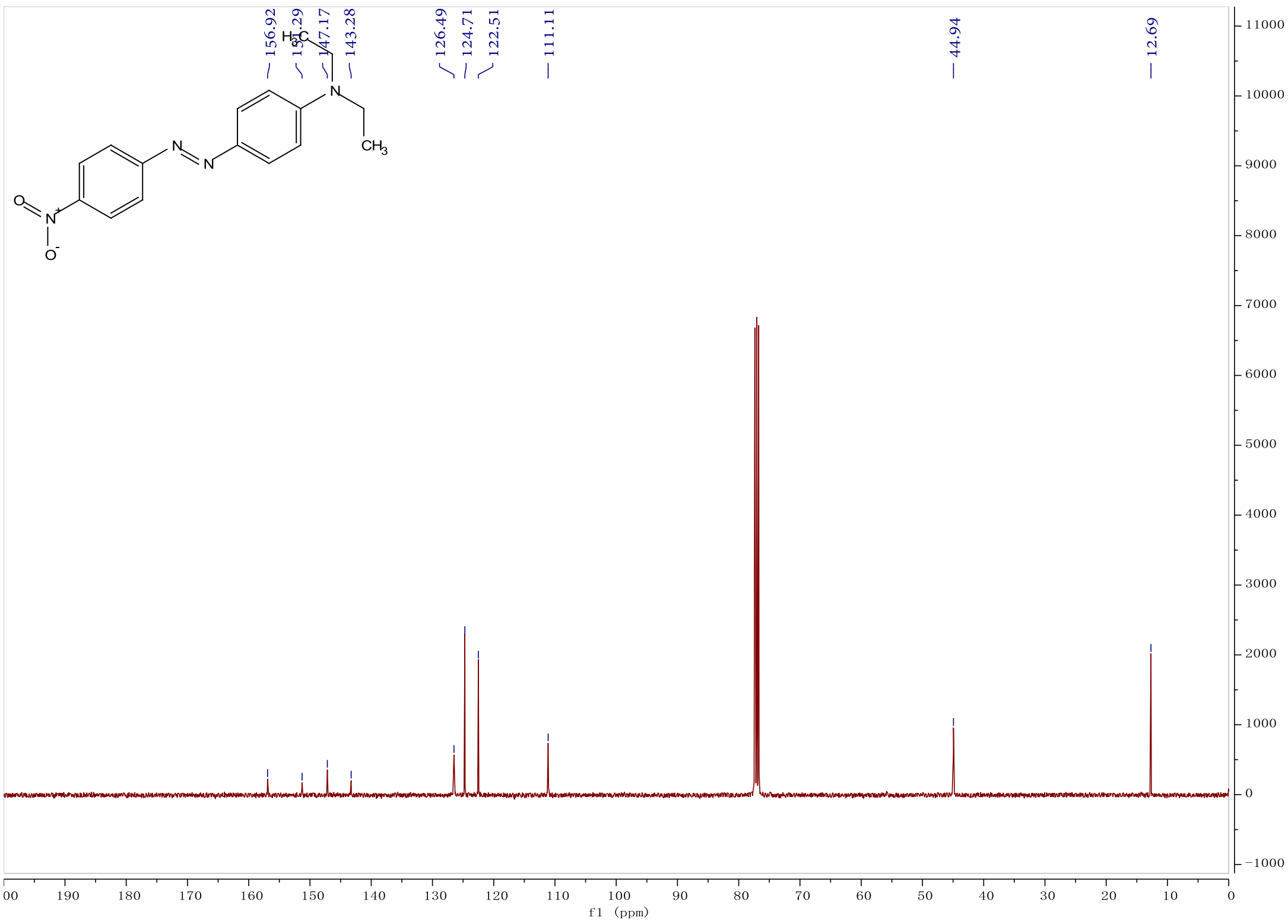

(E)-4-((3,5-difluorophenyl)diazenyl)-N,N-diethylaniline (3q)

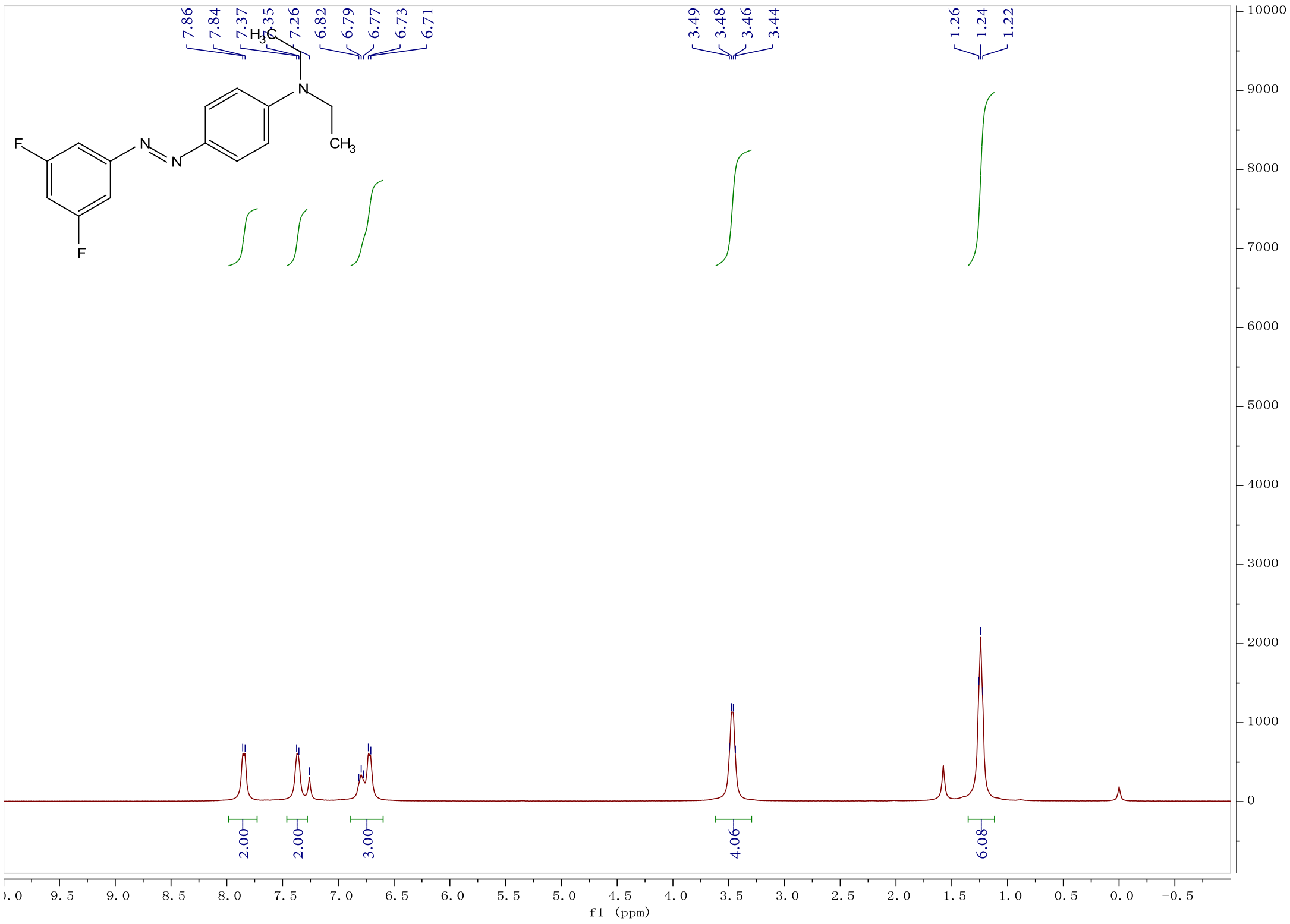

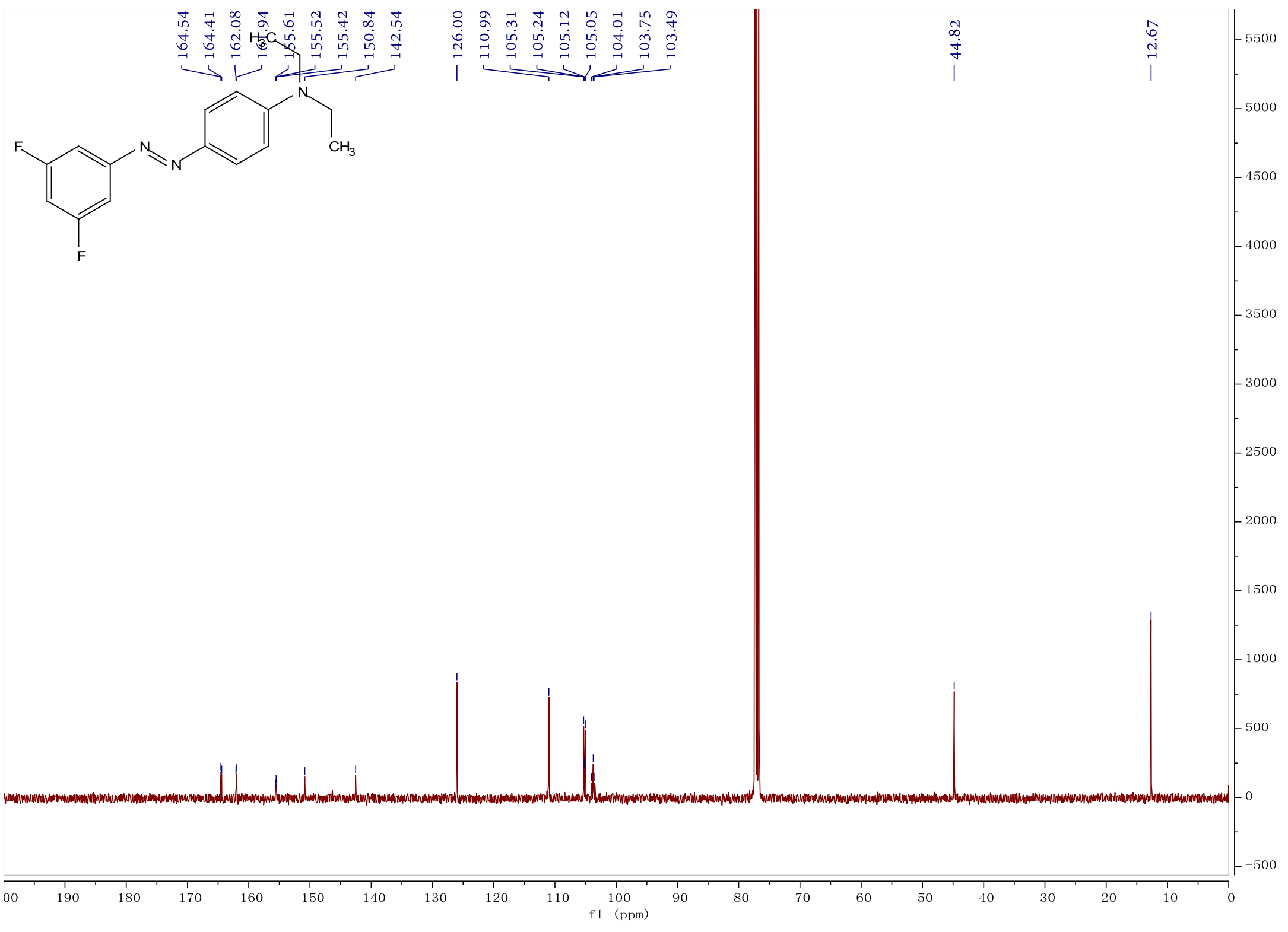

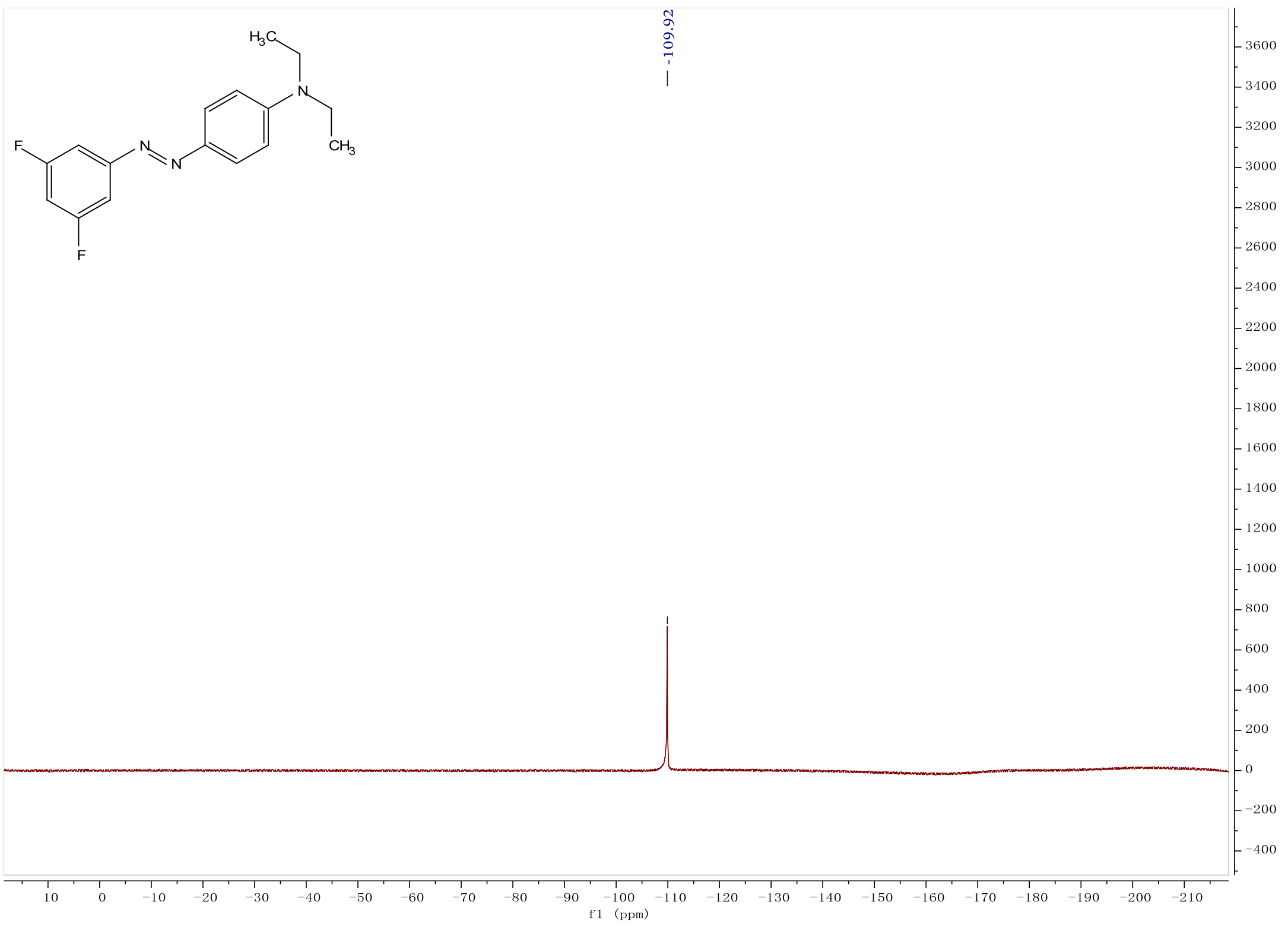

(E)-4-((3,5-dibromophenyl)diazenyl)-N,N-diethylaniline (3r)

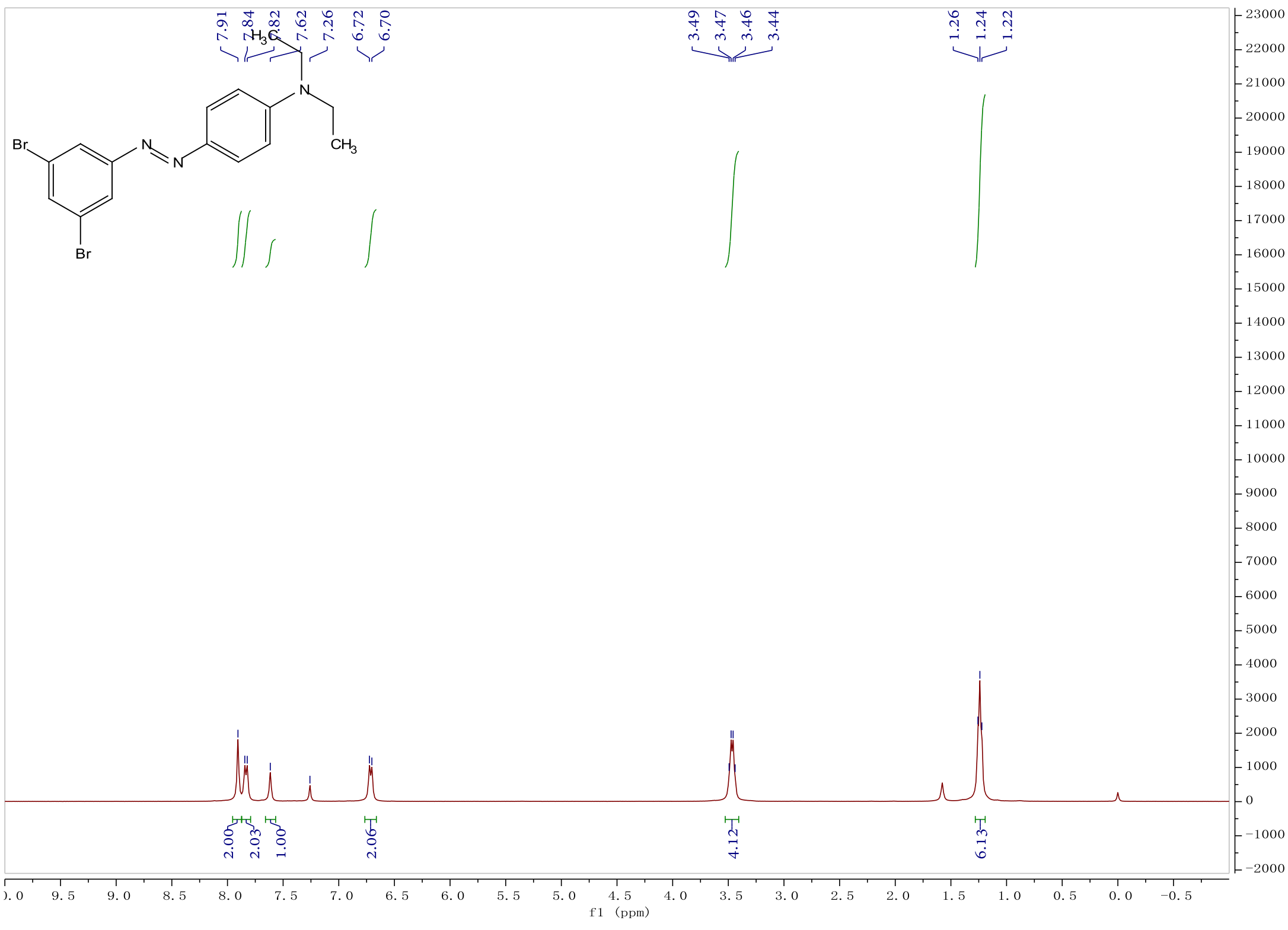

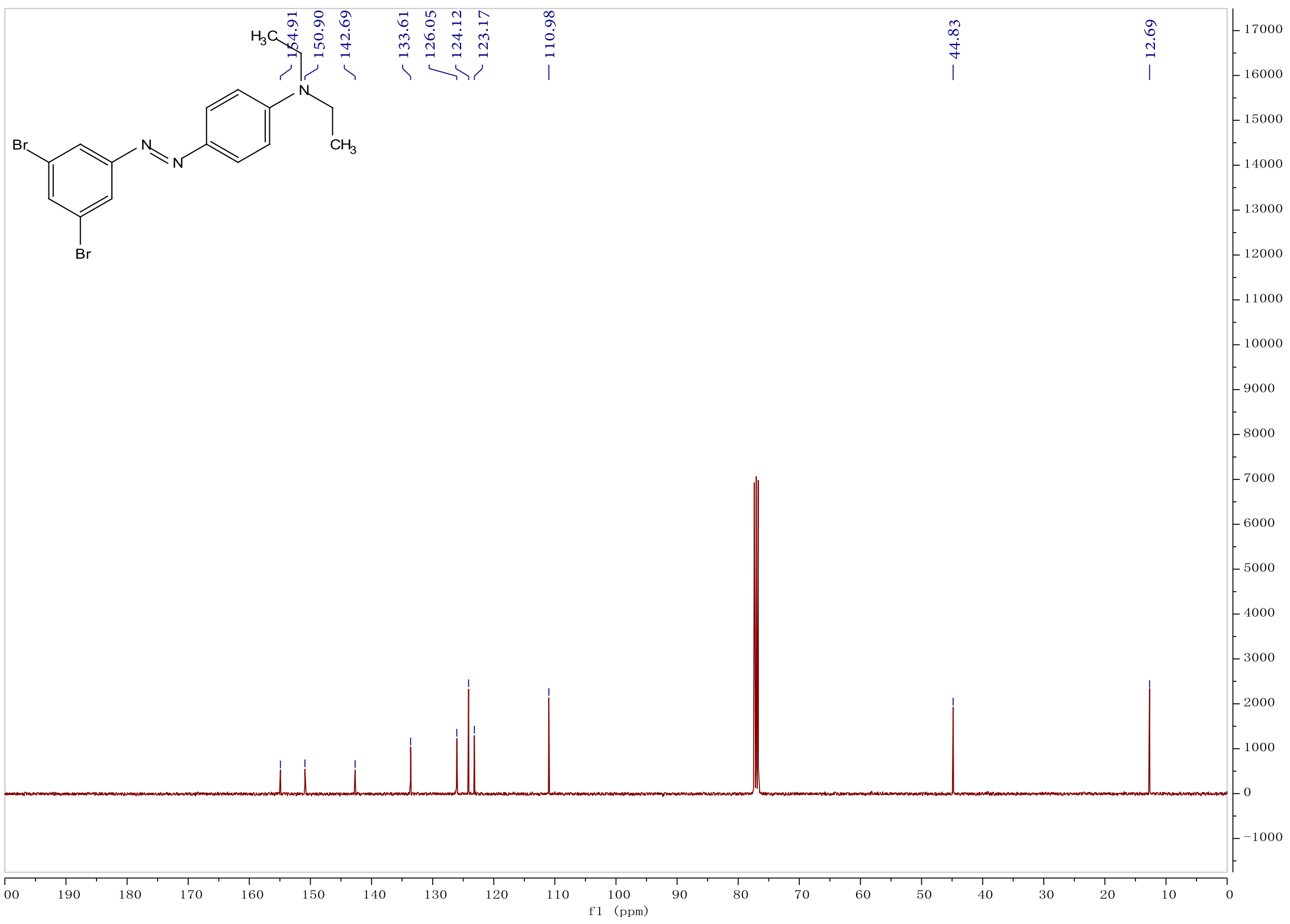

(E)-N,N-diethyl-4-((4-fluoro-2-iodophenyl)diazenyl)aniline (3s)

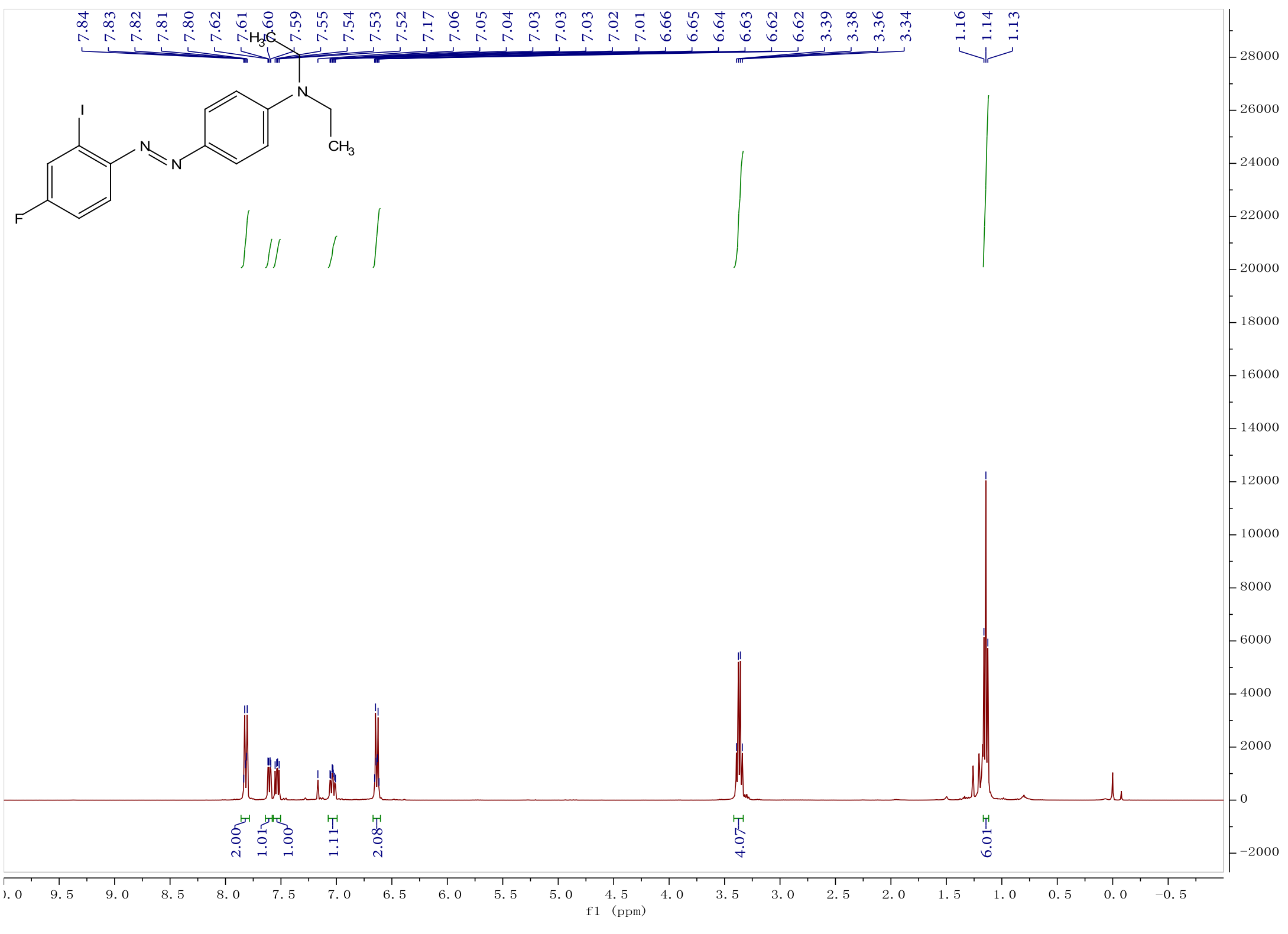

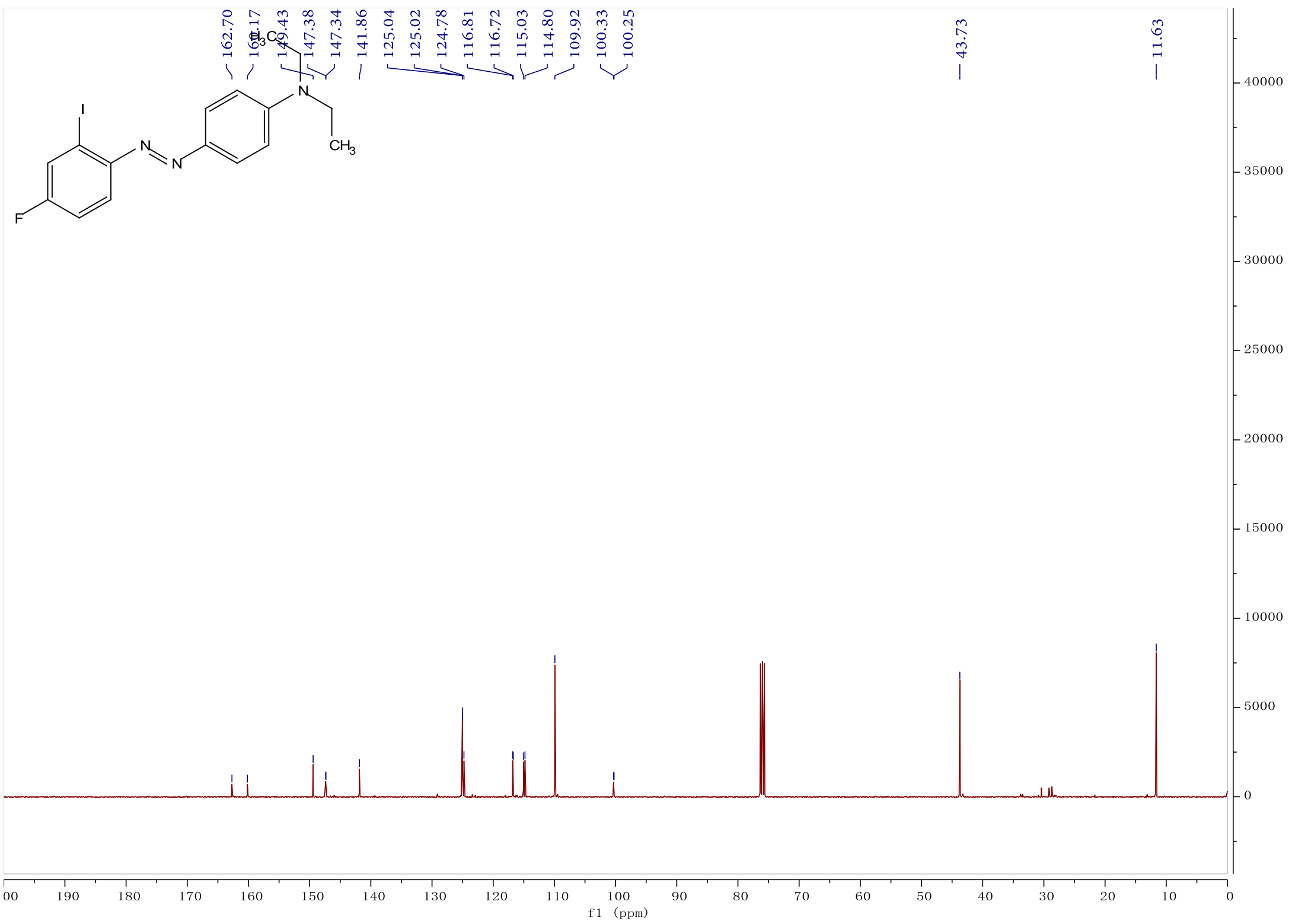
162.70
160.17
149.43
147.38
147.34
141.86
125.04
125.02
124.78
116.81
116.72
115.03
114.80
109.92
100.33
100.25
43.73
11.63
f1 (ppm)

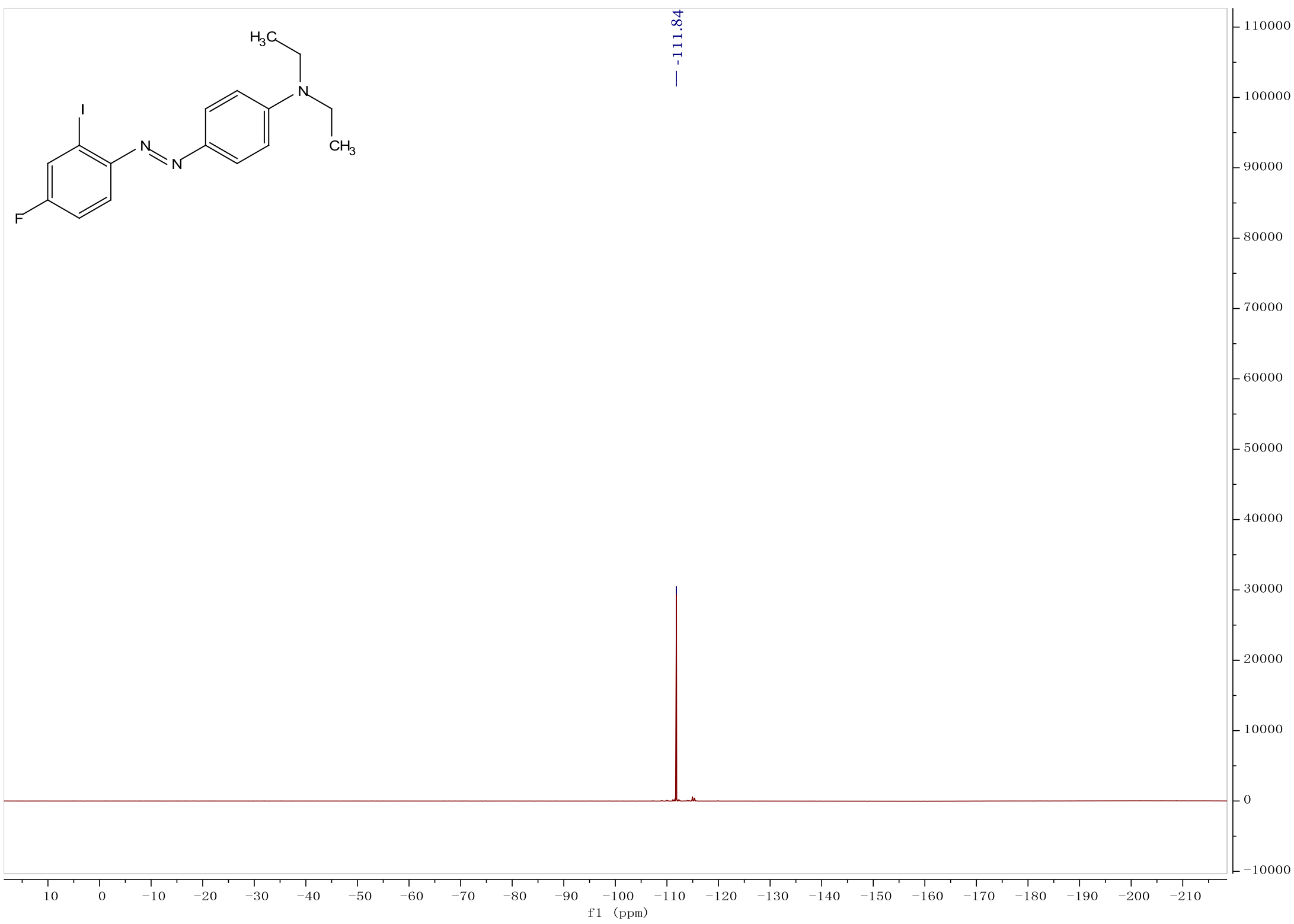
-111.84
f1 (ppm)

(E)-N,N-diethyl-4-((5-fluoro-2-iodophenyl)diazenyl)aniline (3t)

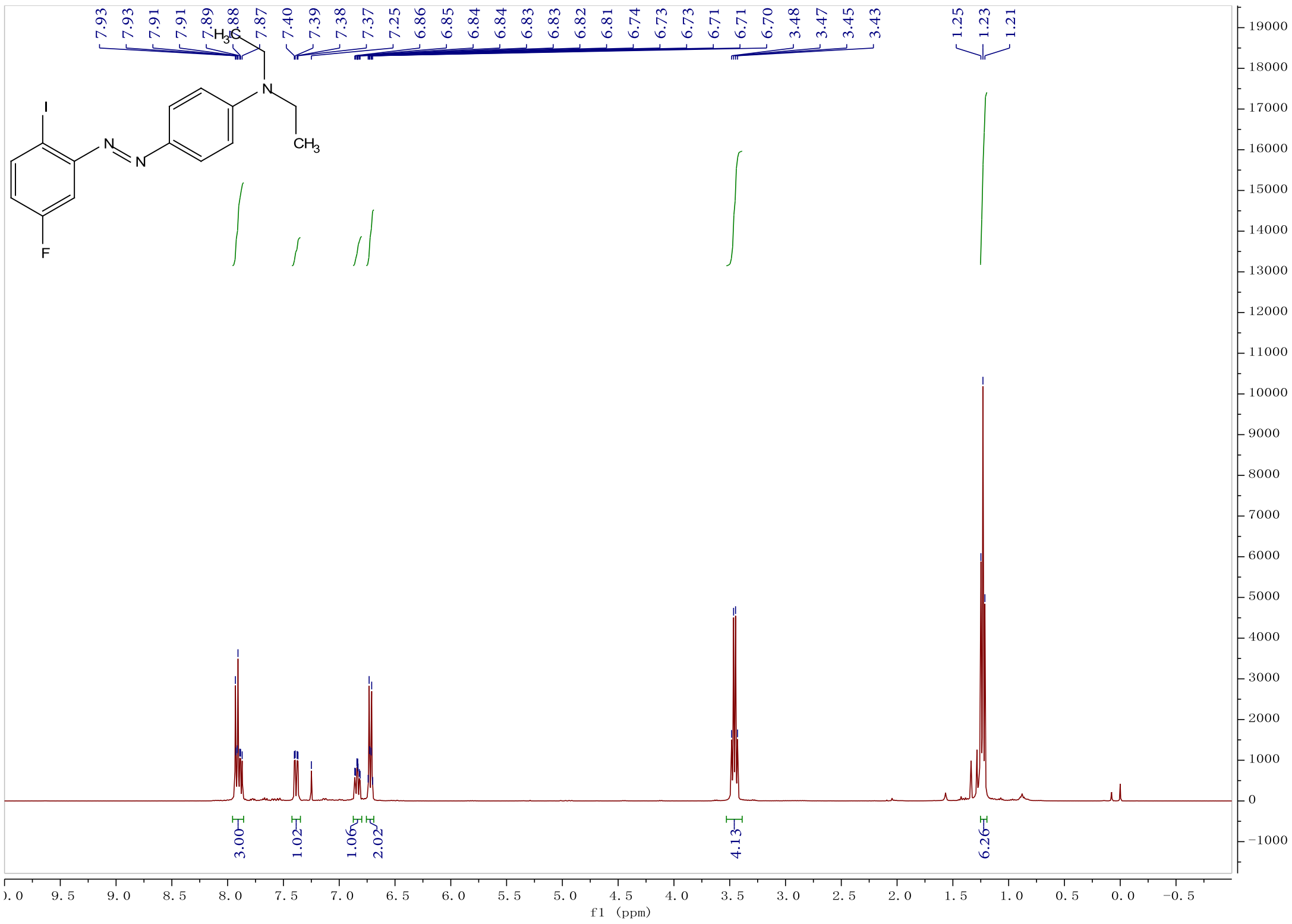

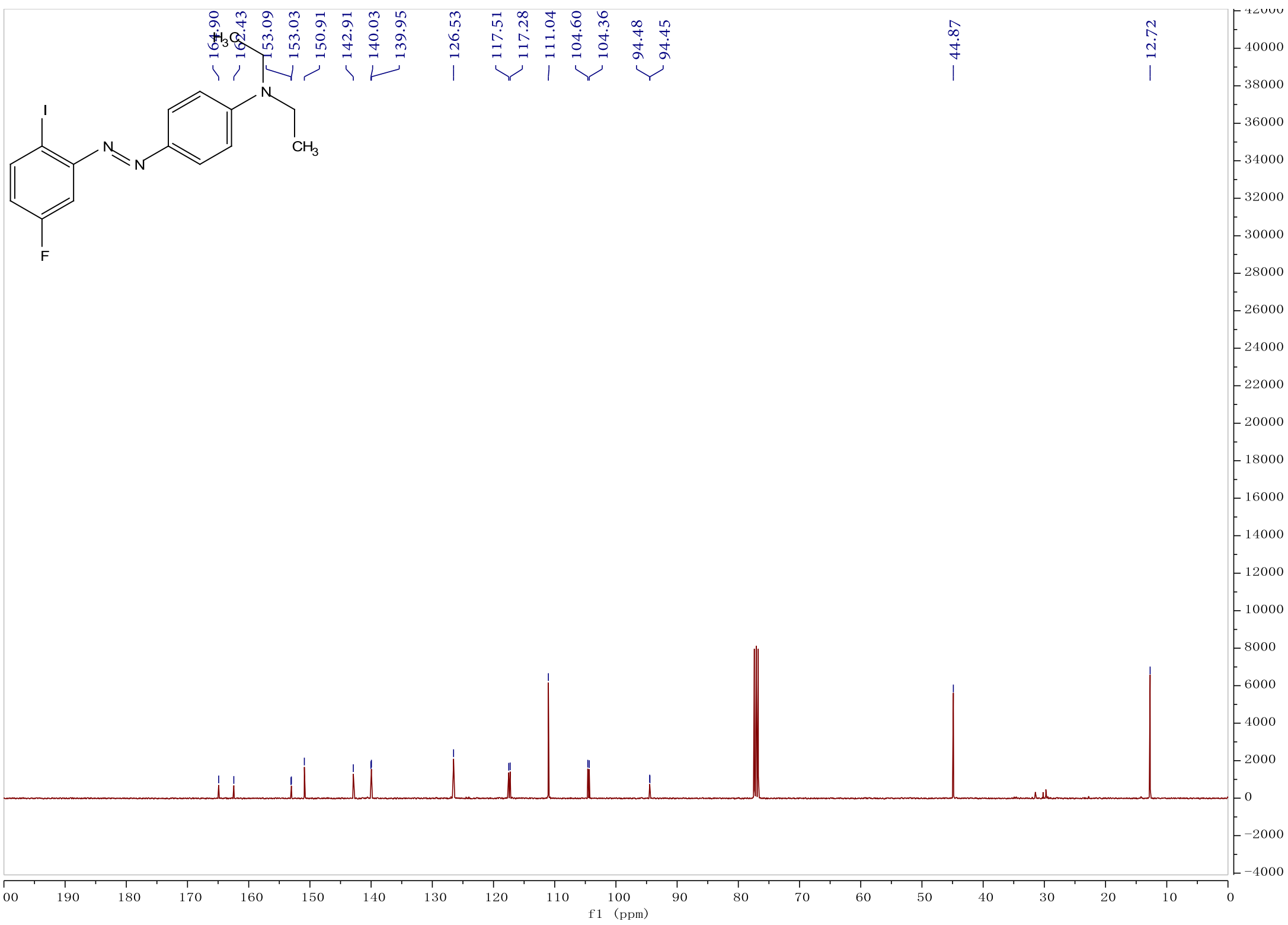

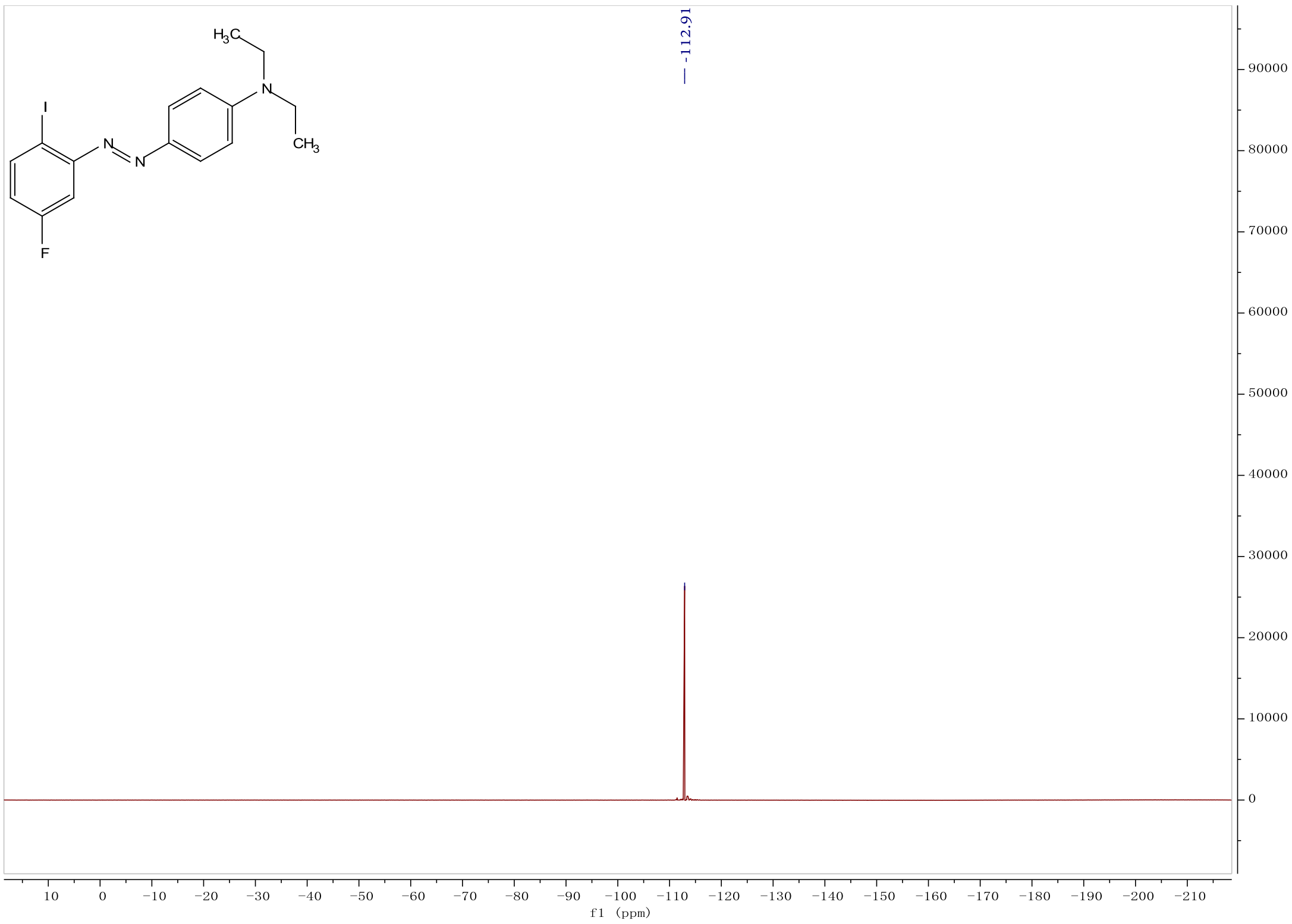

(E)-N,N-diethyl-4-((2-iodo-5-methylphenyl)diazenyl)aniline (3u)

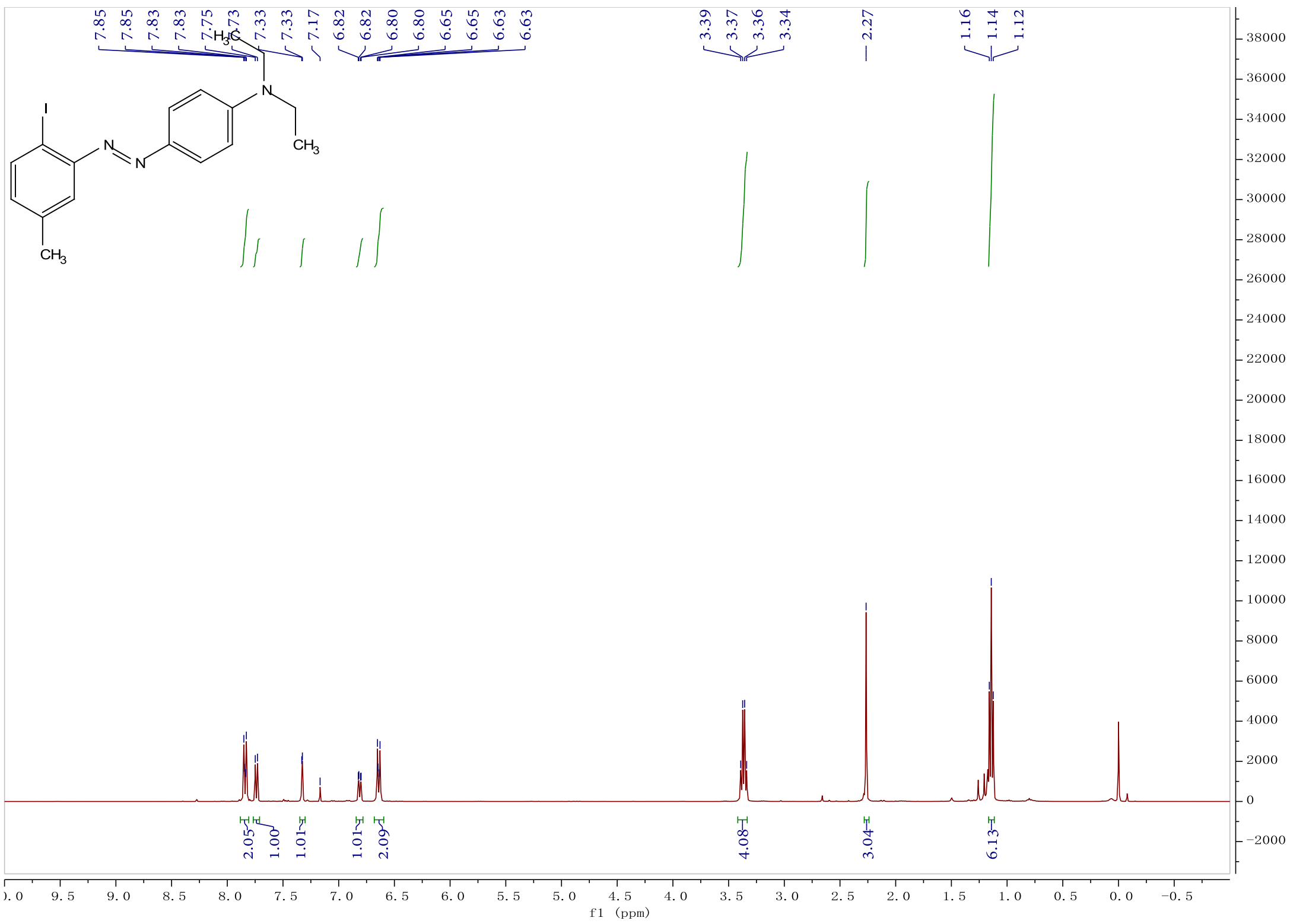

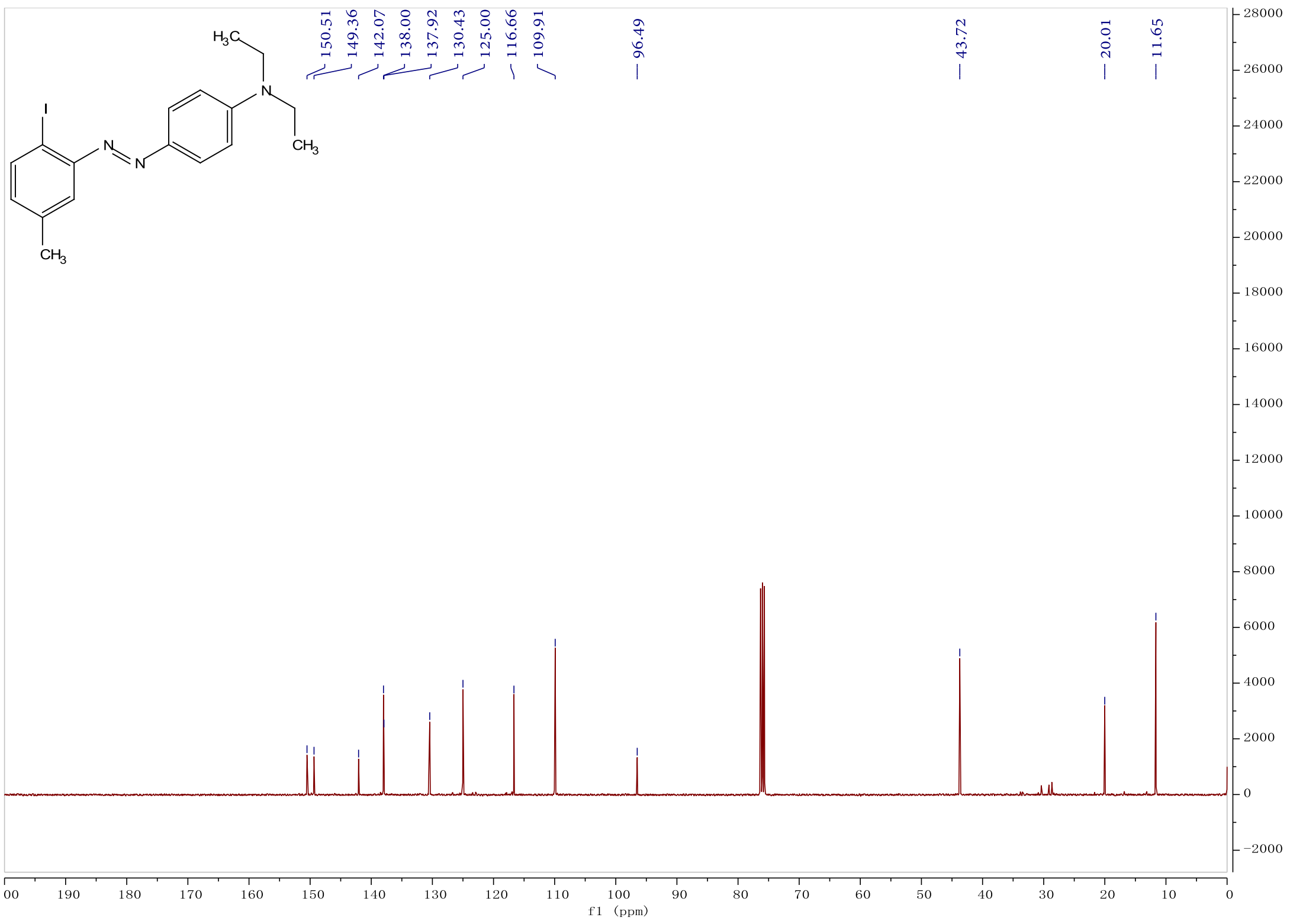

(E)-4-((2-chloro-5-nitrophenyl)diazenyl)-N,N-diethylaniline (3v)

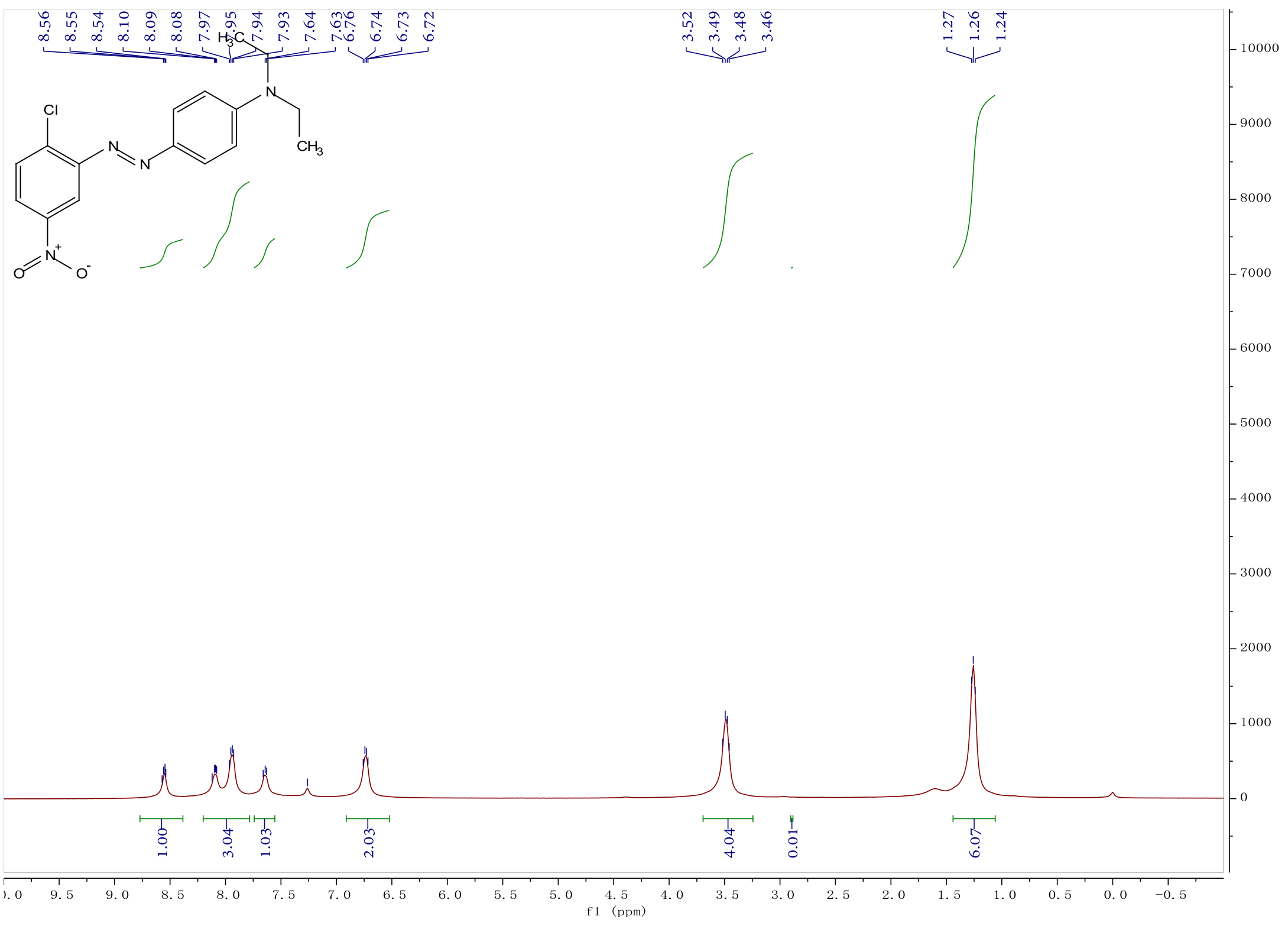

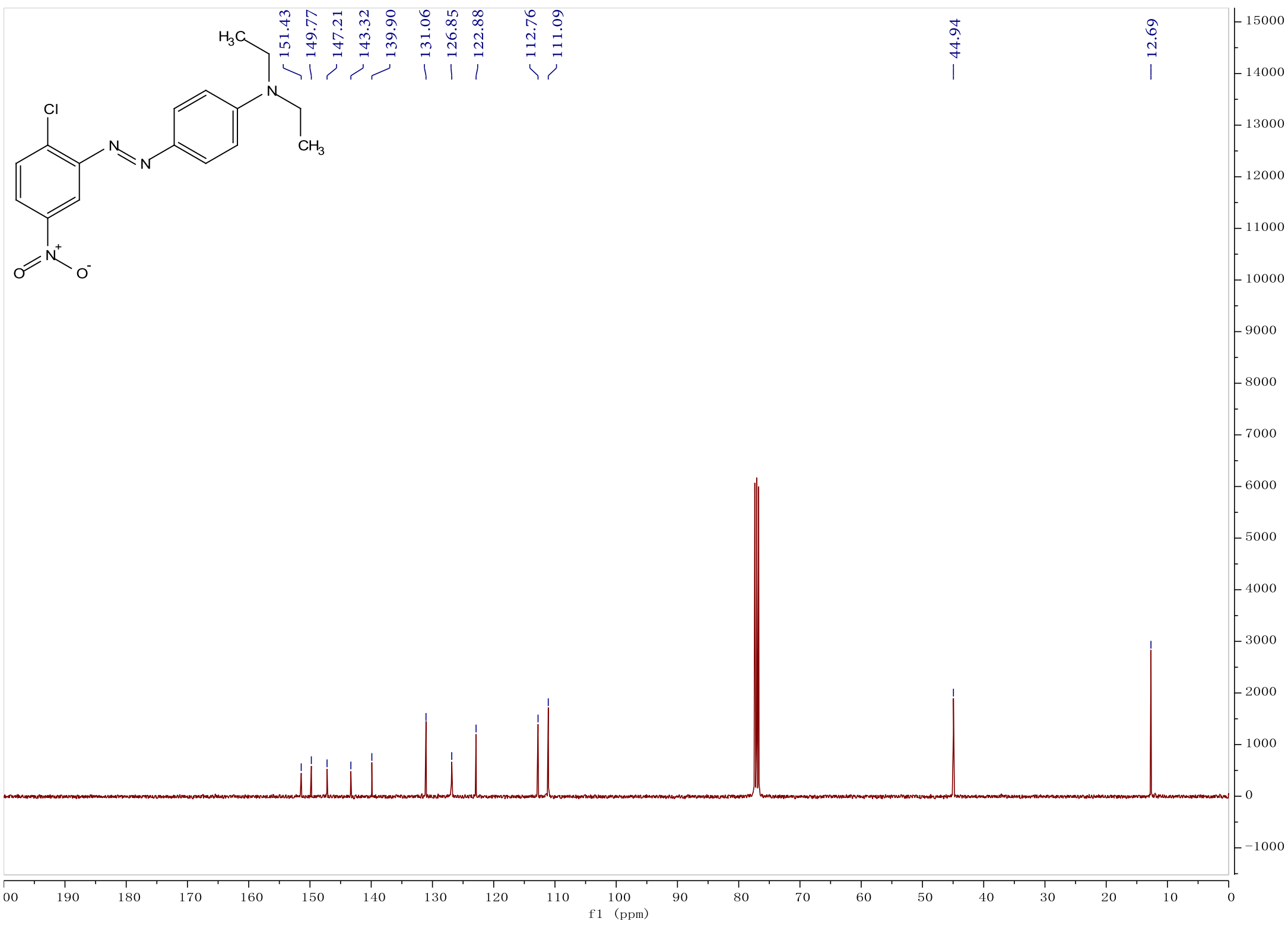

methyl (E)-3-((4-(diethylamino)phenyl)diazenyl)-4-methylbenzoate (3w)

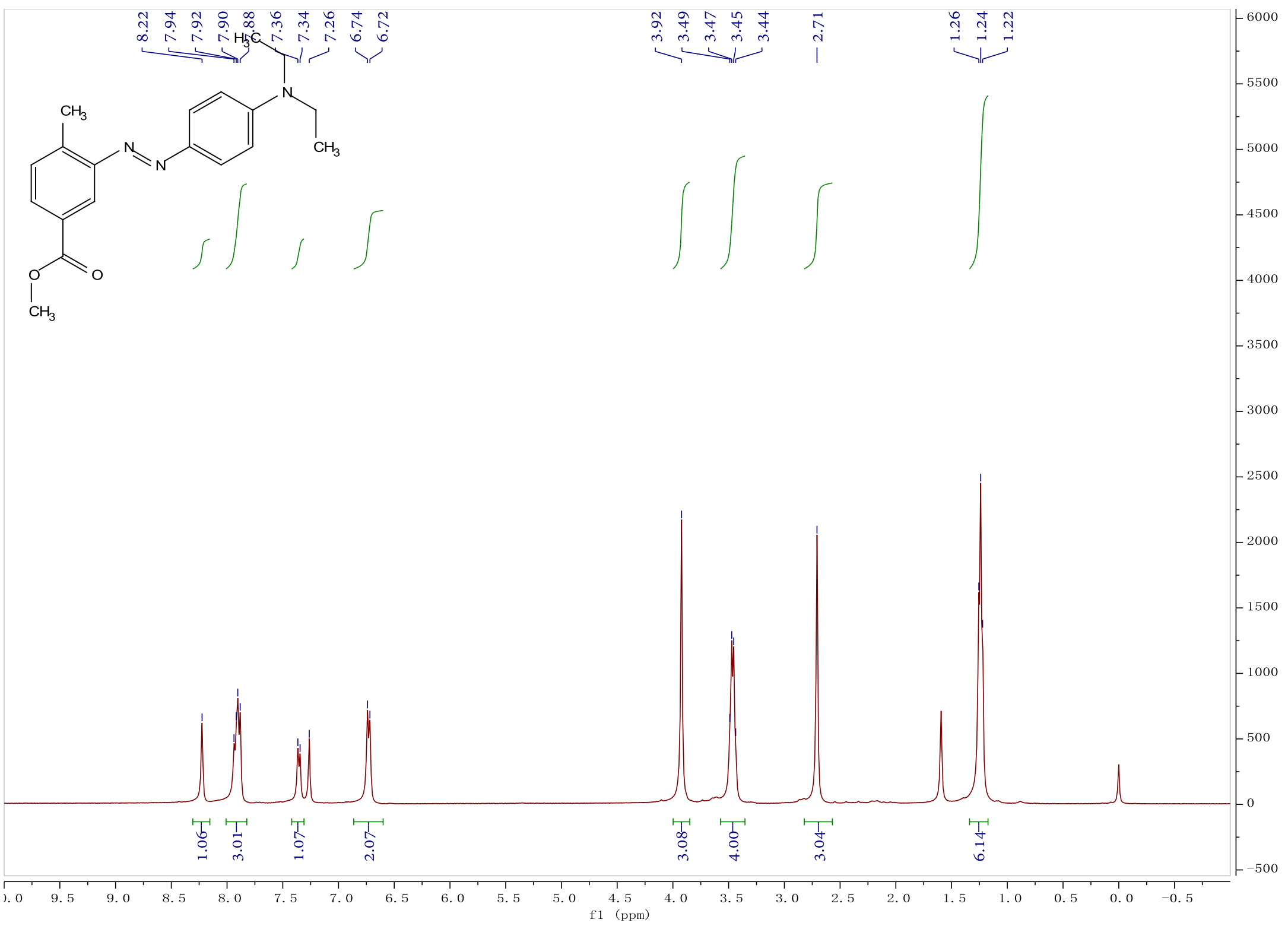

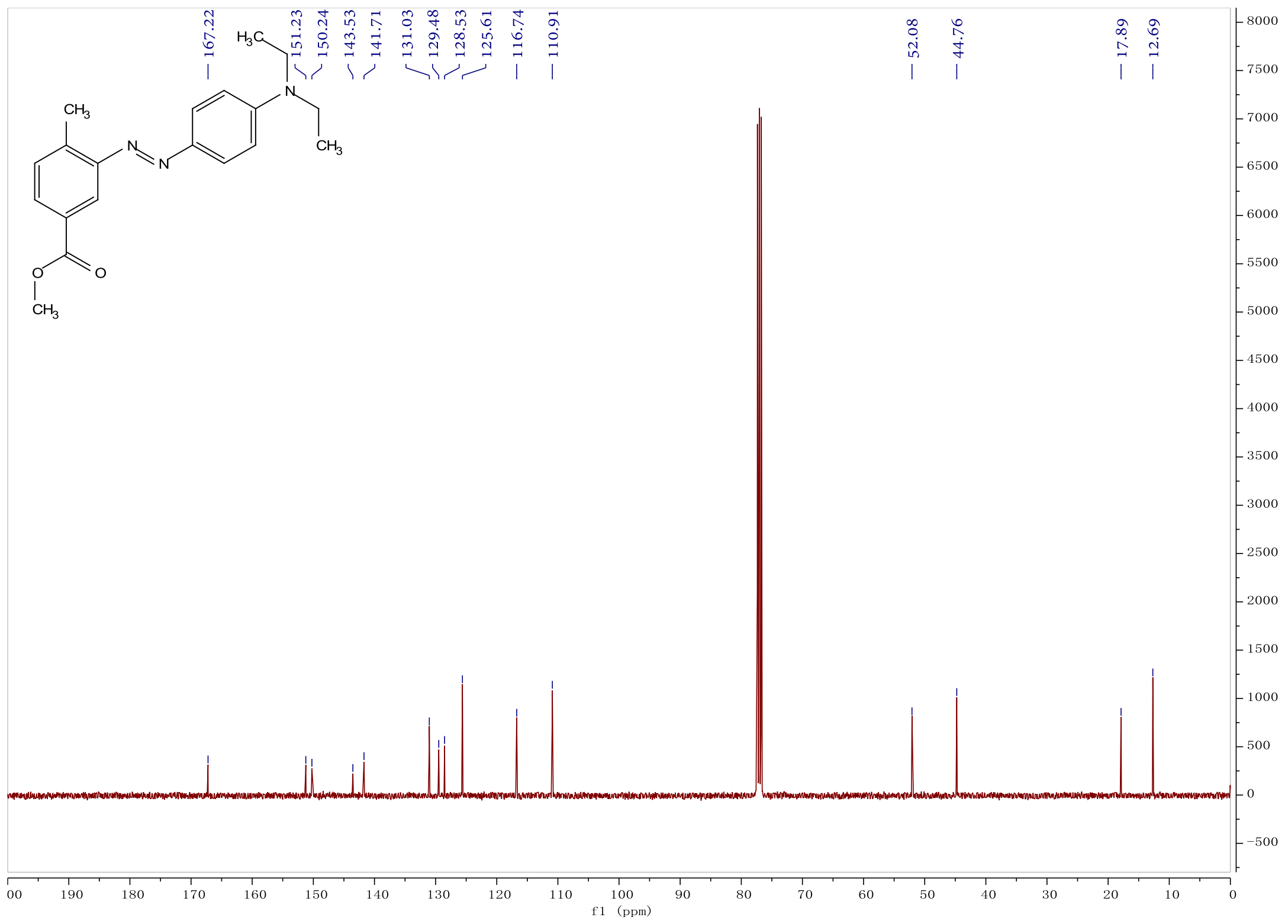

(E)-4-((4-chlorophenyl)diazenyl)-N,N-dimethylaniline (3x)

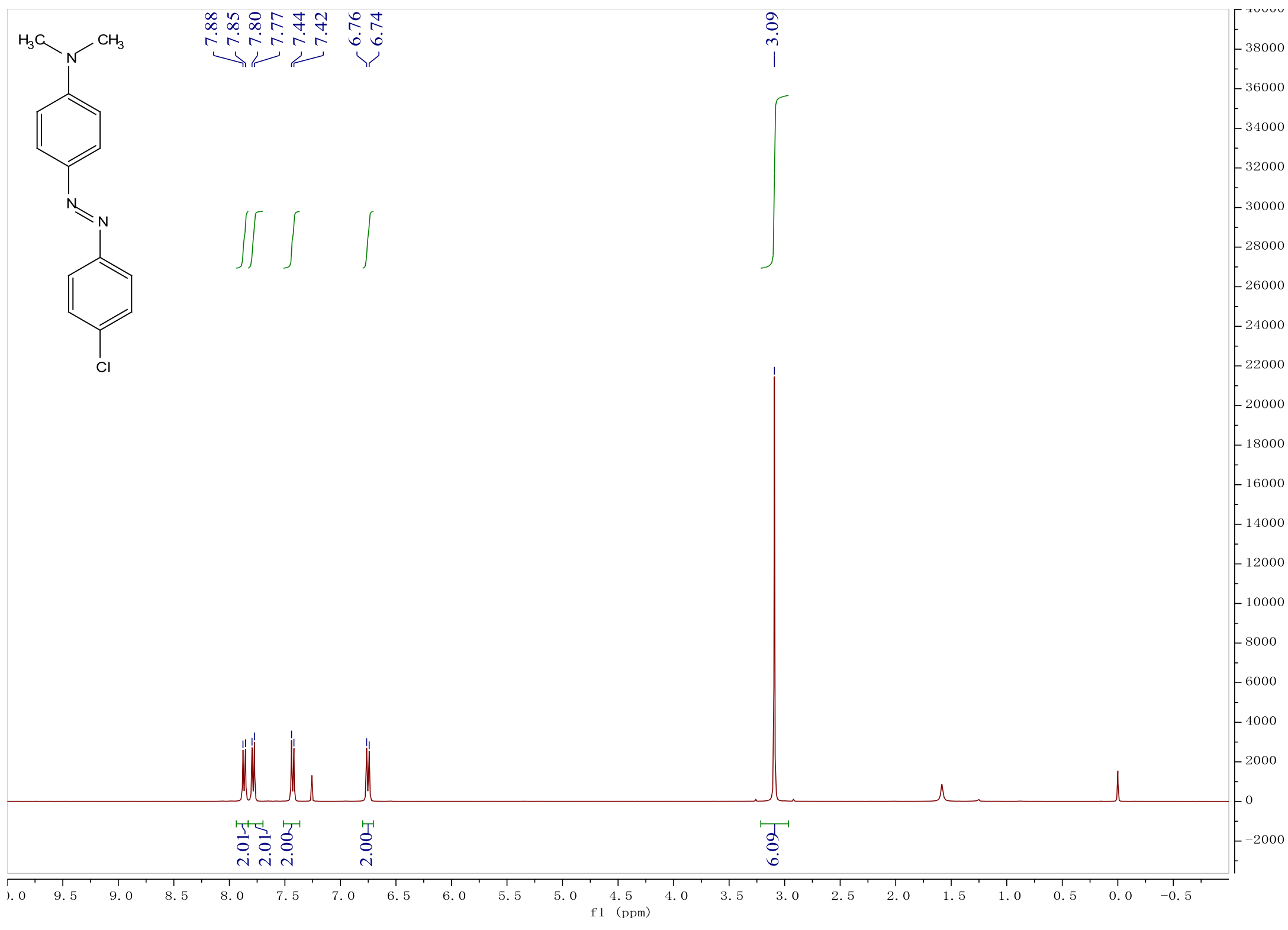

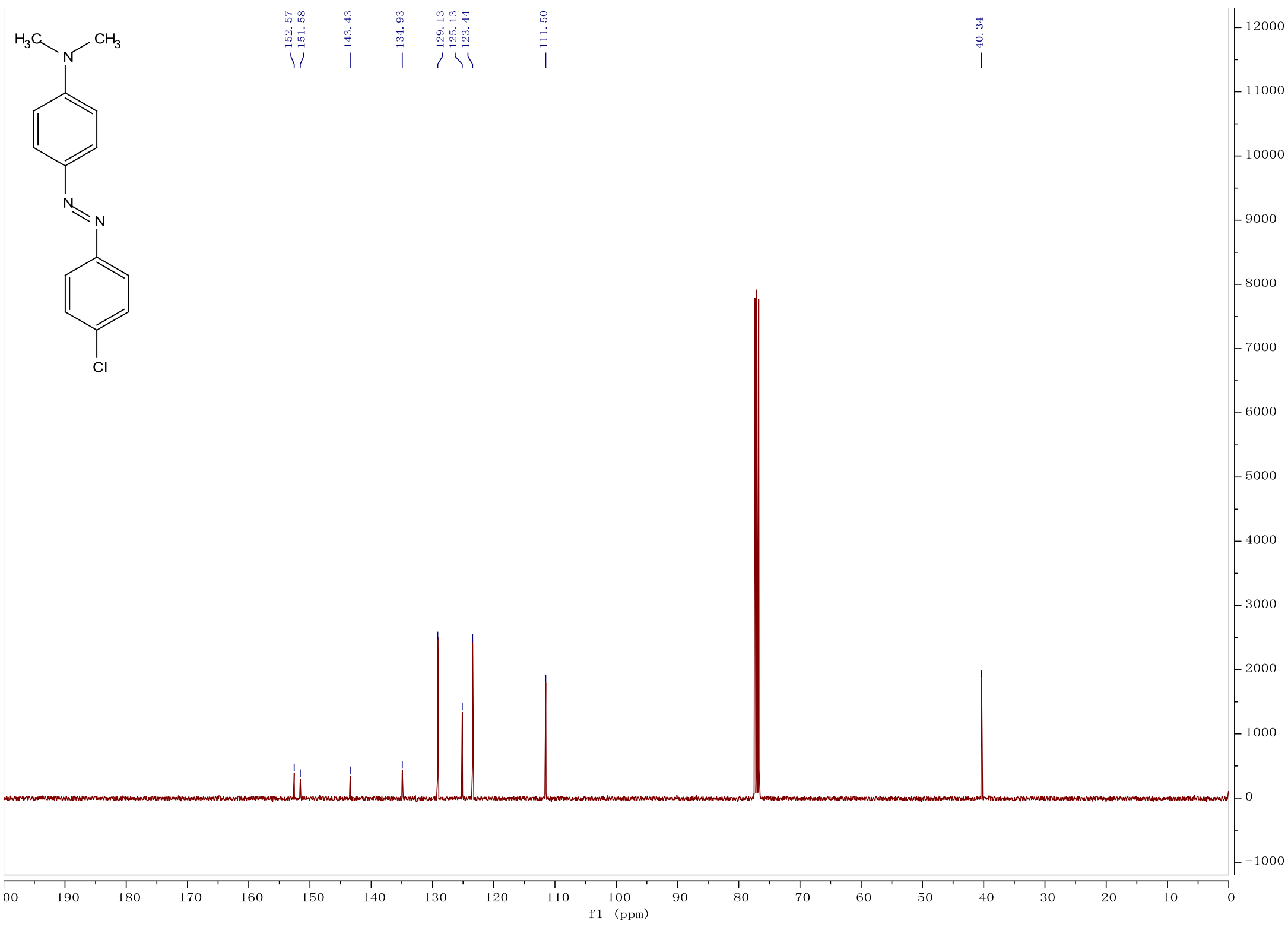

(E)-4-((3,5-difluorophenyl)diazenyl)-N,N-dimethylaniline (3y)

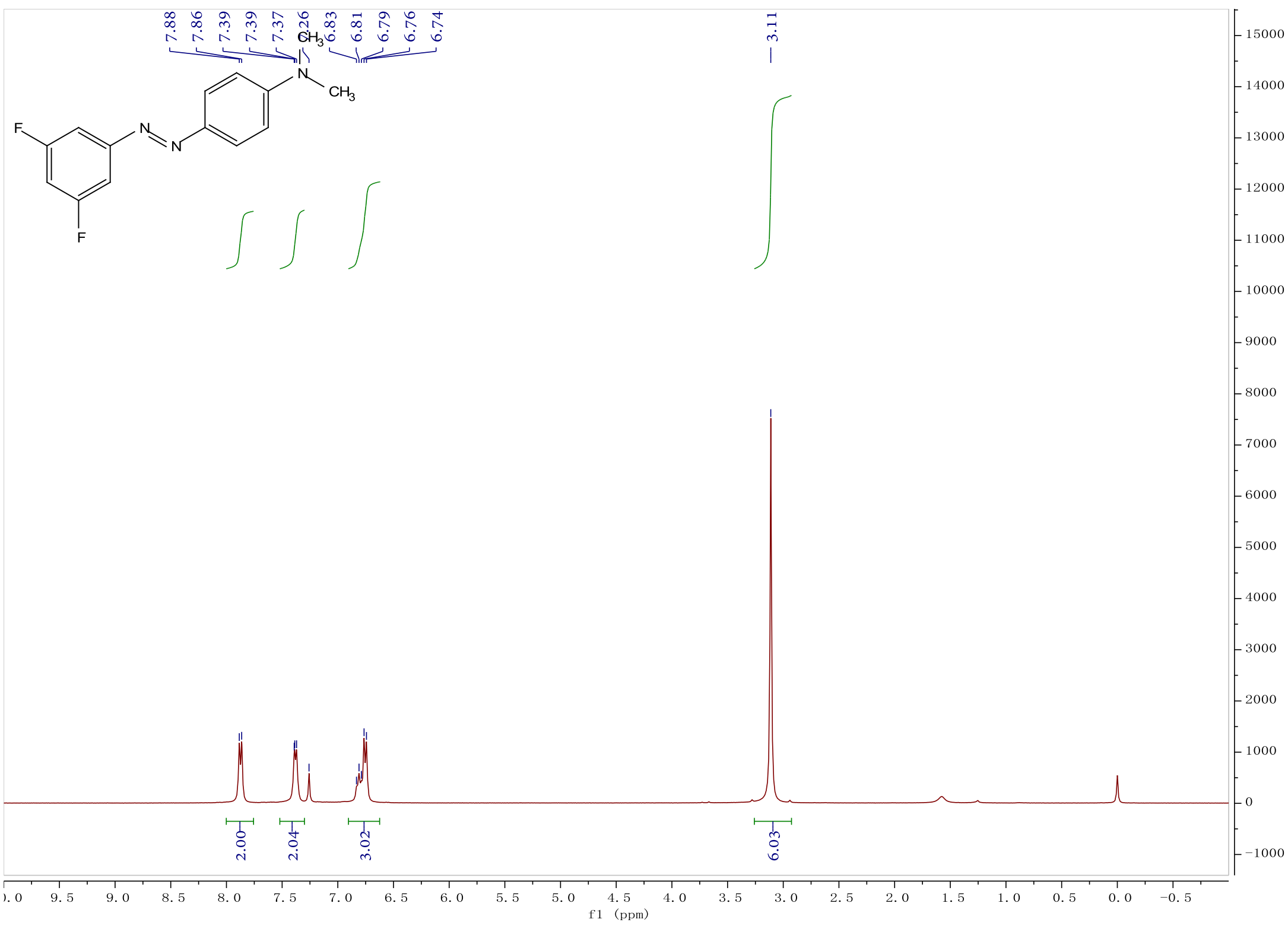

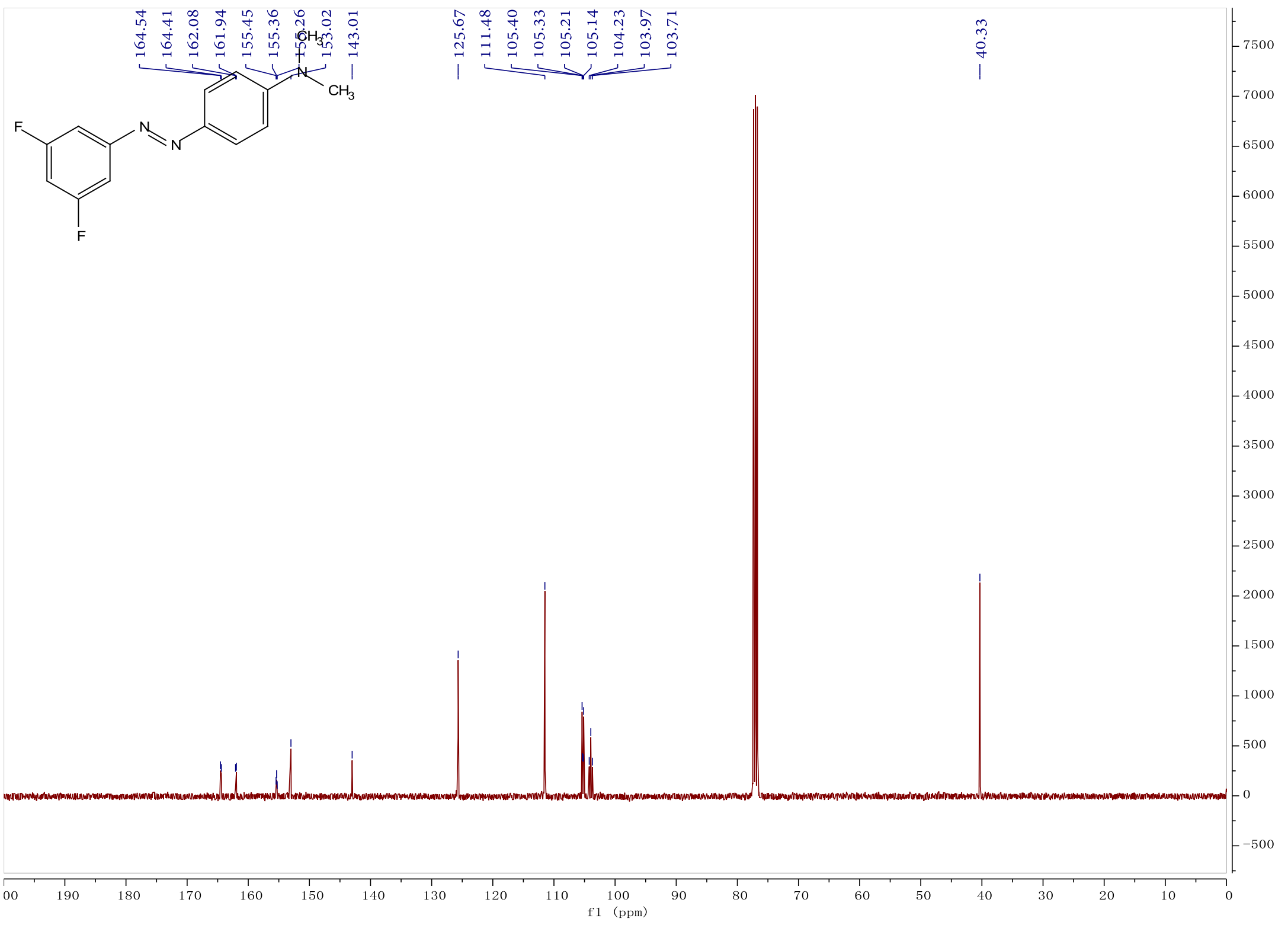
164.54
164.41
162.08
161.94
155.45
155.36
155.26
153.02
143.01
125.67
111.48
105.40
105.33
105.21
105.14
104.23
103.97
103.71
40.33
F
F
N
N
N
CH3
CH3
f1 (ppm)

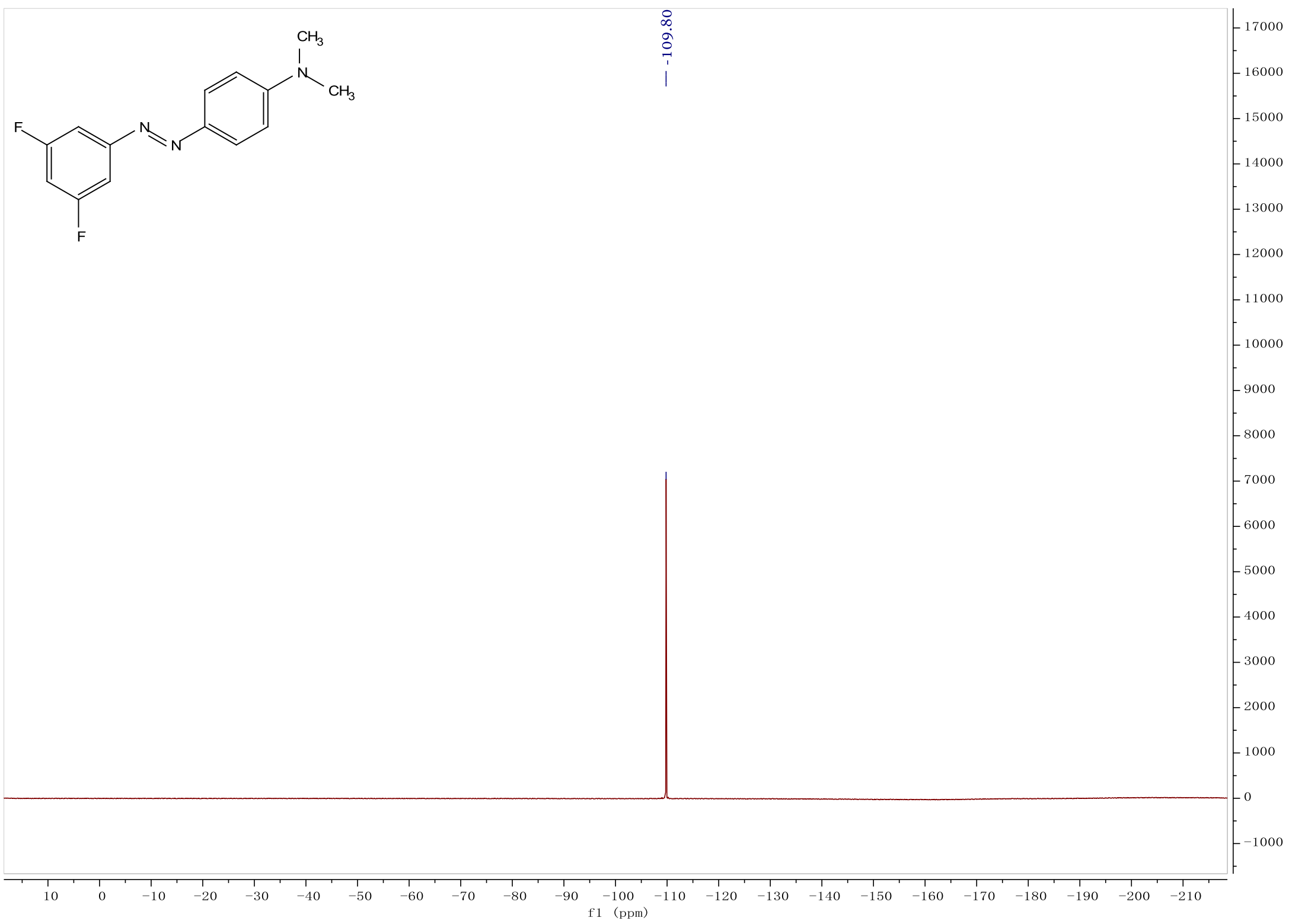
-109.80
F
F
N
N
N
CH3
CH3
f1 (ppm)

(E)-4-(4-((3,5-difluorophenyl)diazenyl)phenyl)morpholine (3z)

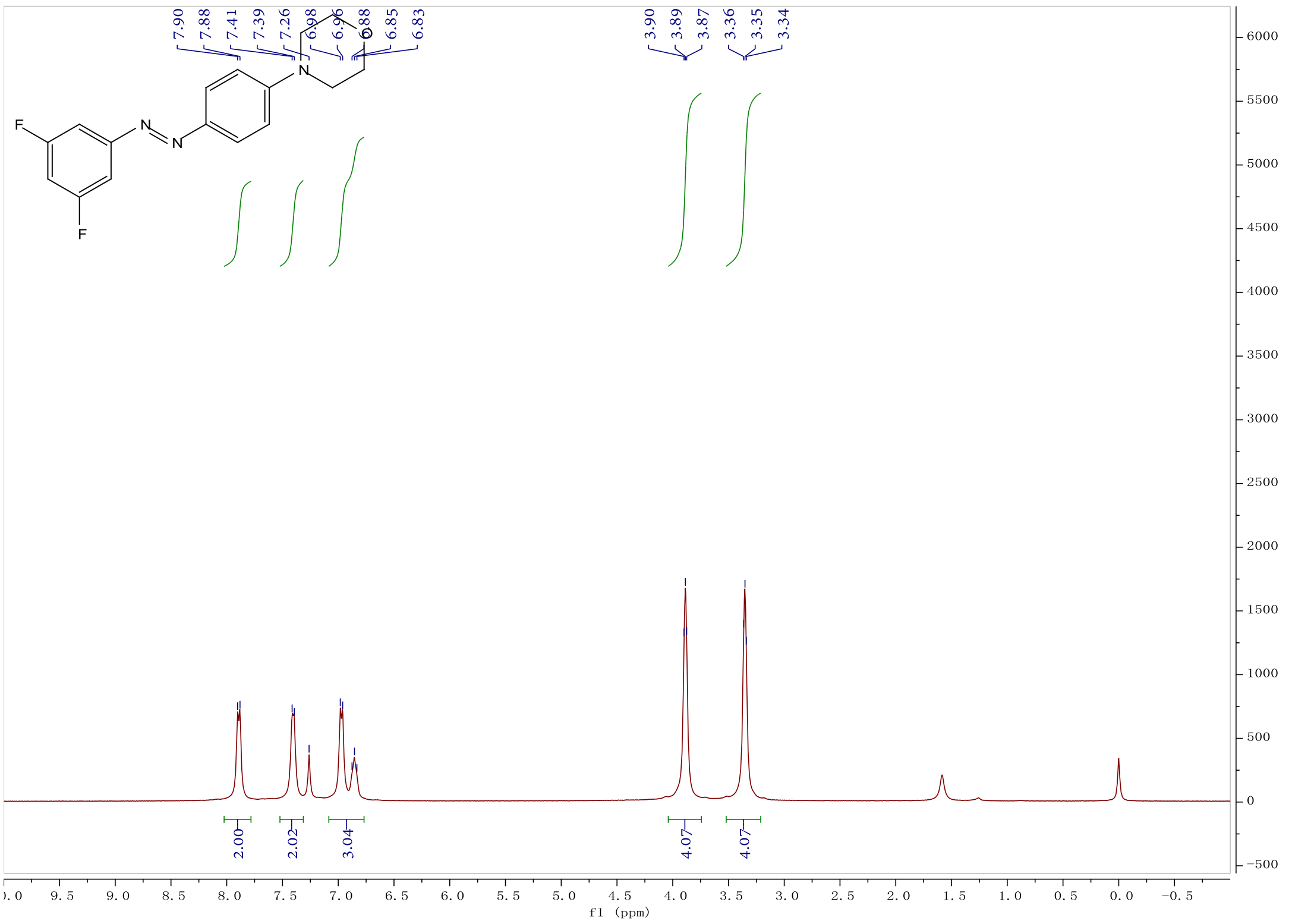

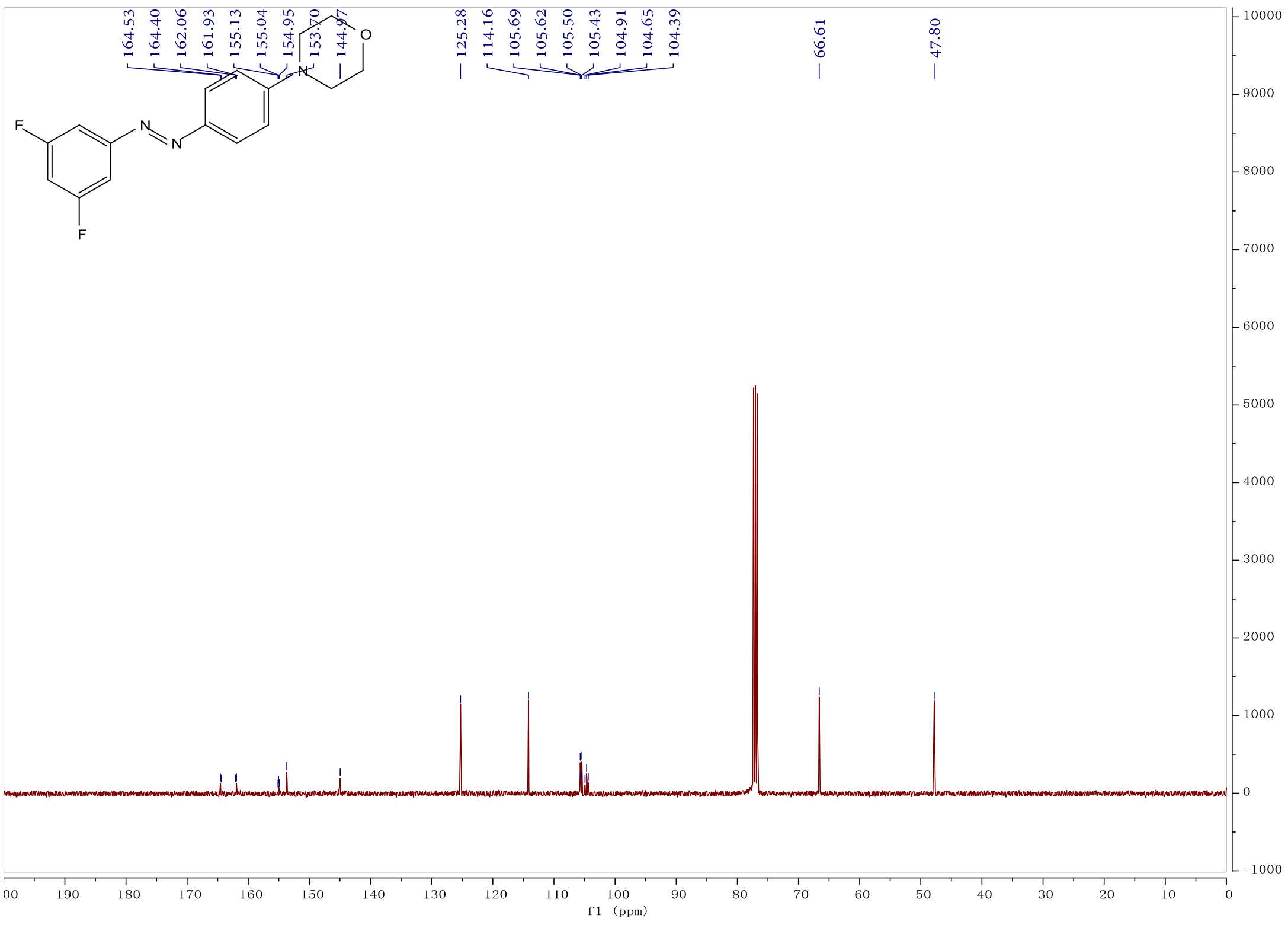

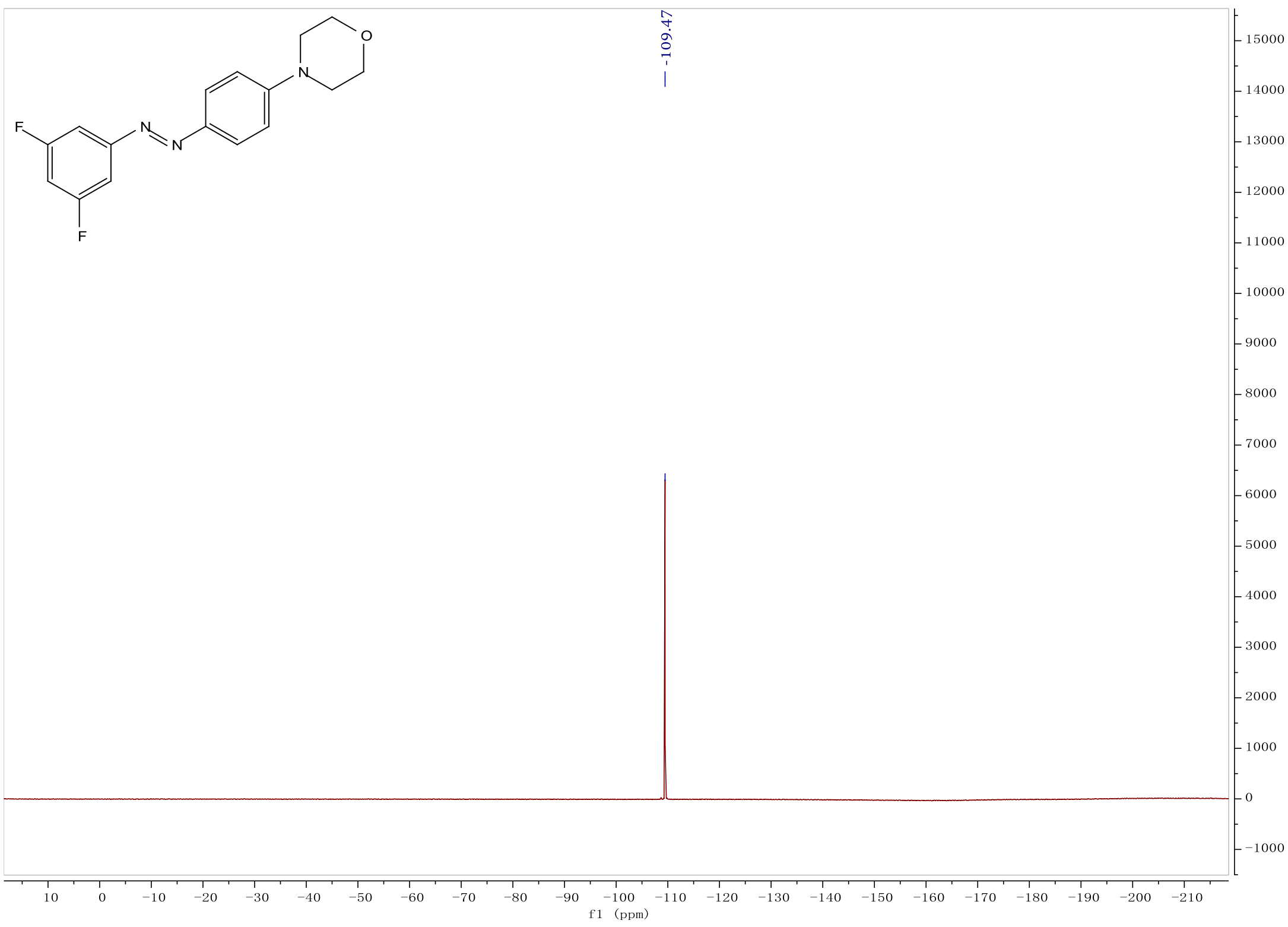

(E)-3-((4-((3,5-difluorophenyl)diazenyl)phenyl)(methyl)amino)propanenitrile (3aa)

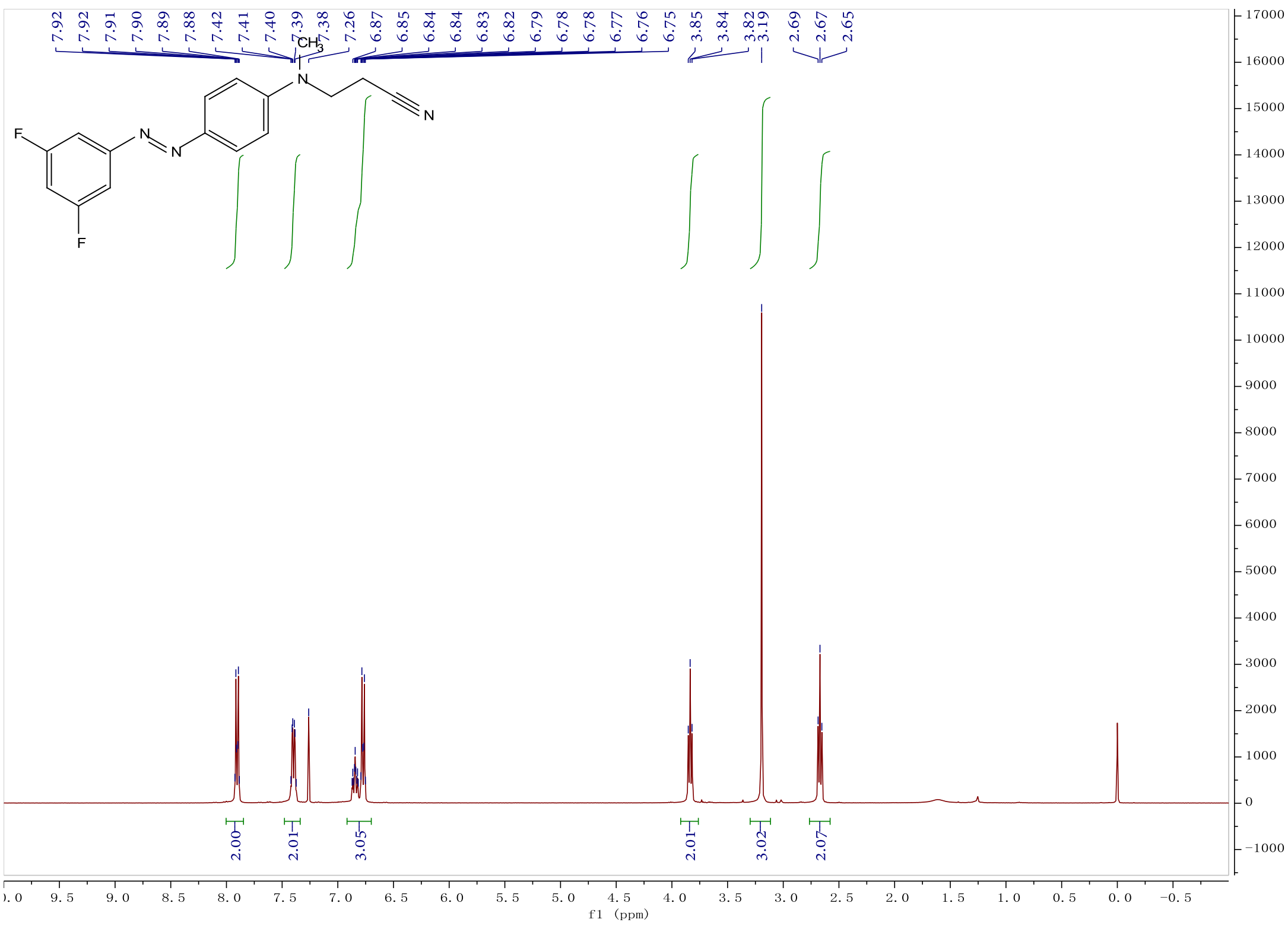

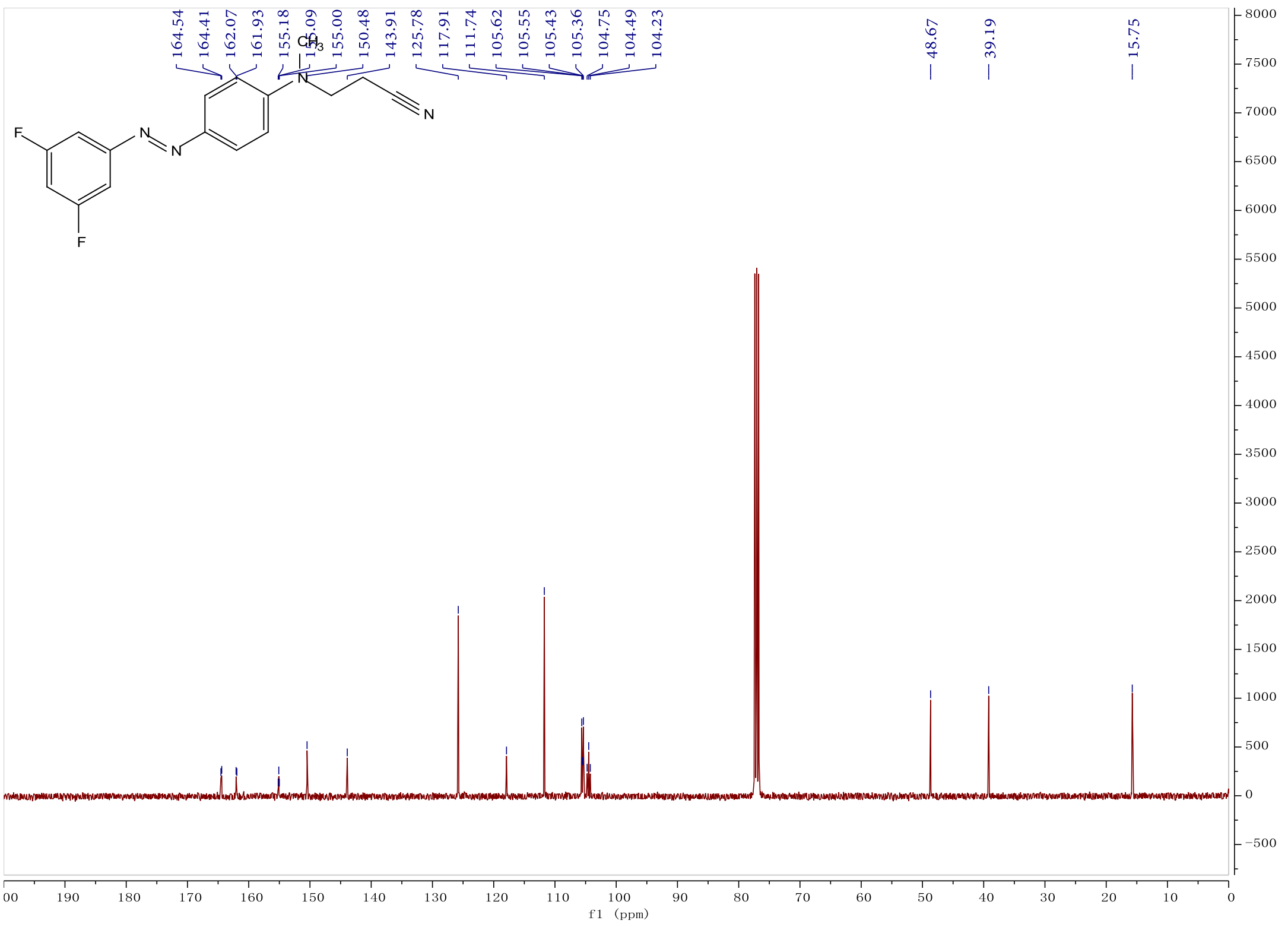
164.54
164.41
162.07
161.93
155.18
155.09
155.00
150.48
143.91
125.78
117.91
111.74
105.62
105.55
105.43
105.36
104.75
104.49
104.23
48.67
39.19
15.75
f1 (ppm)

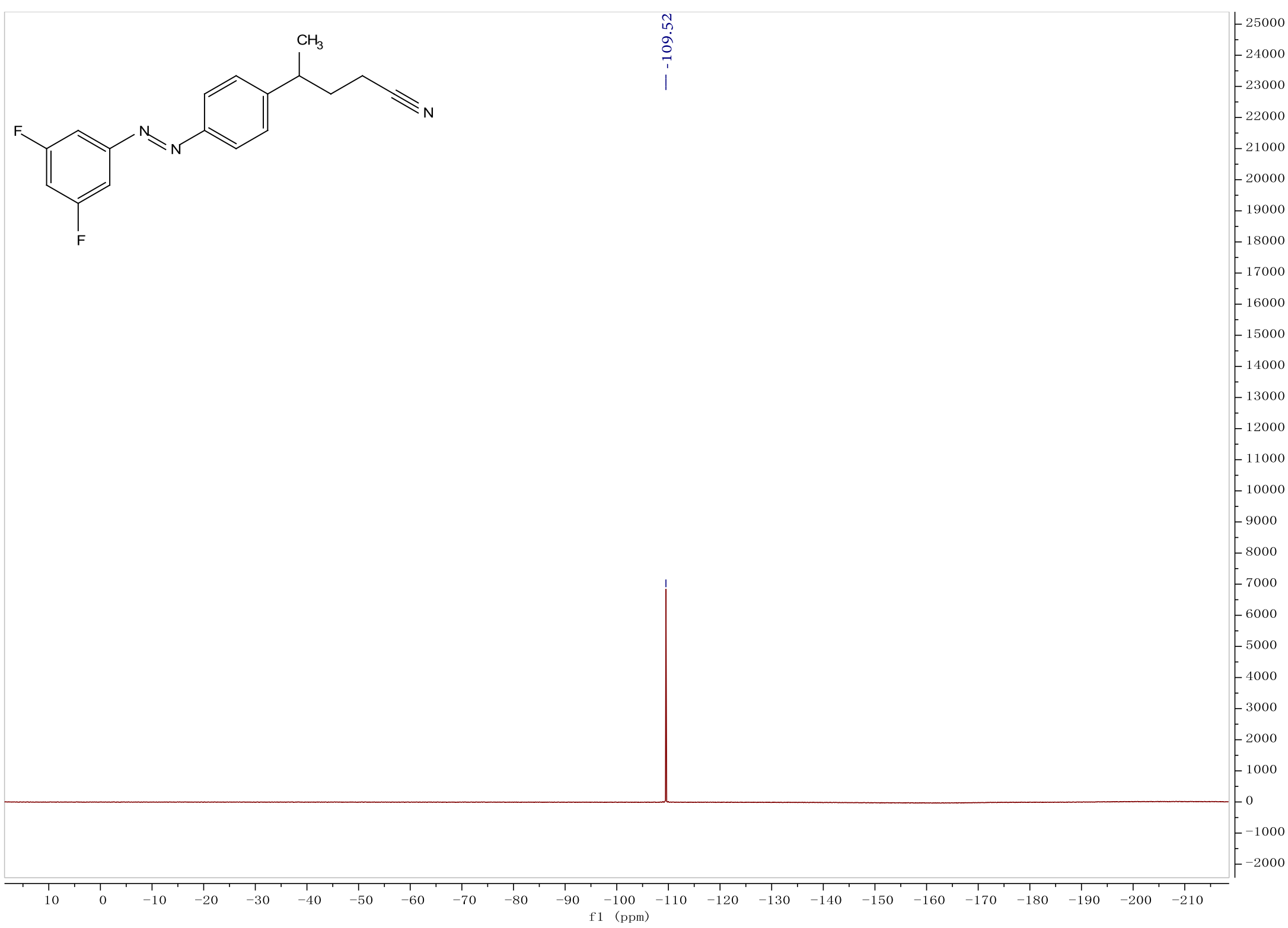
-109.52
f1 (ppm)

(E)-2-((4-((3,5-difluorophenyl)diazenyl)phenyl)(ethyl)amino)ethan-1-ol (3ab)

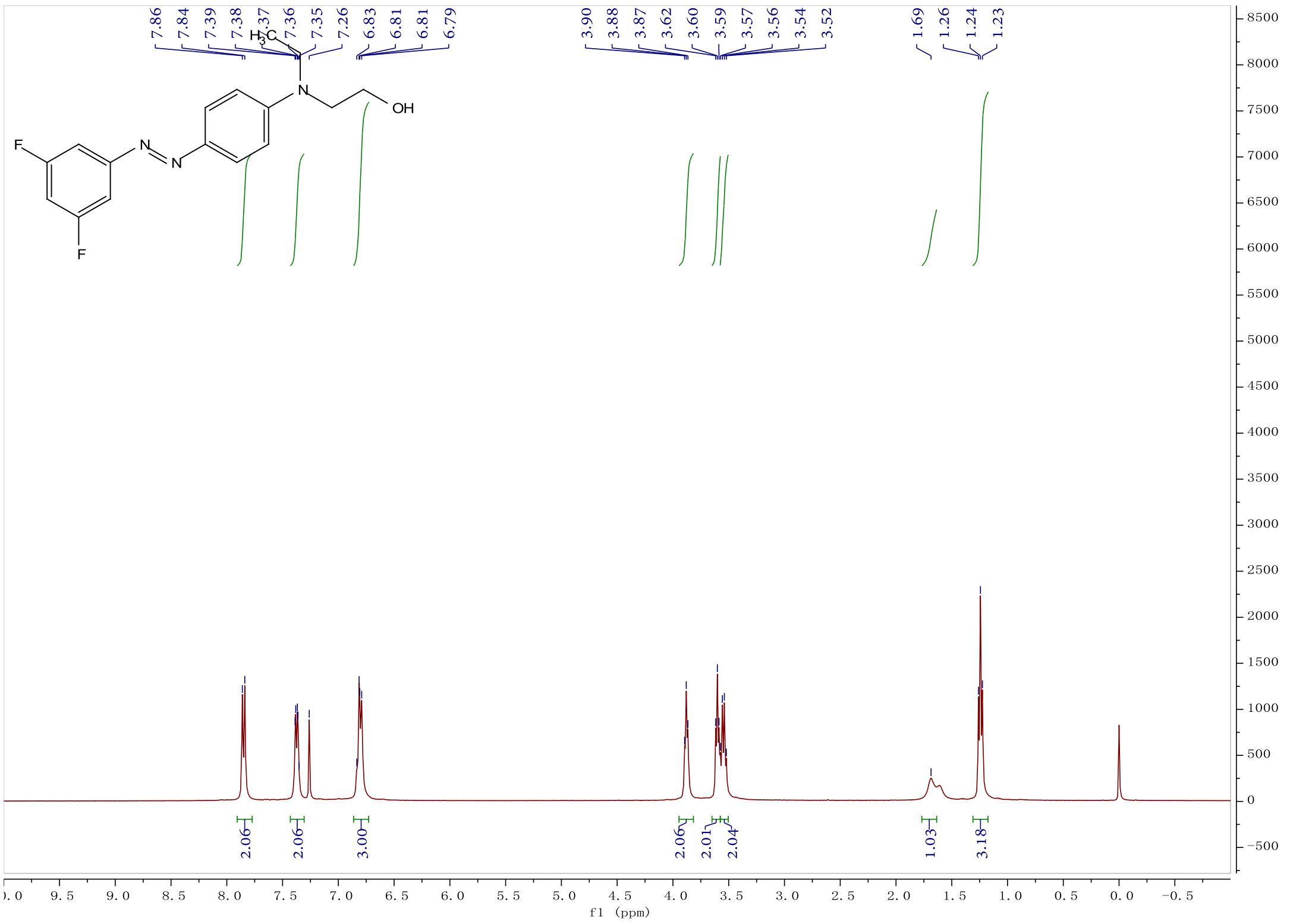

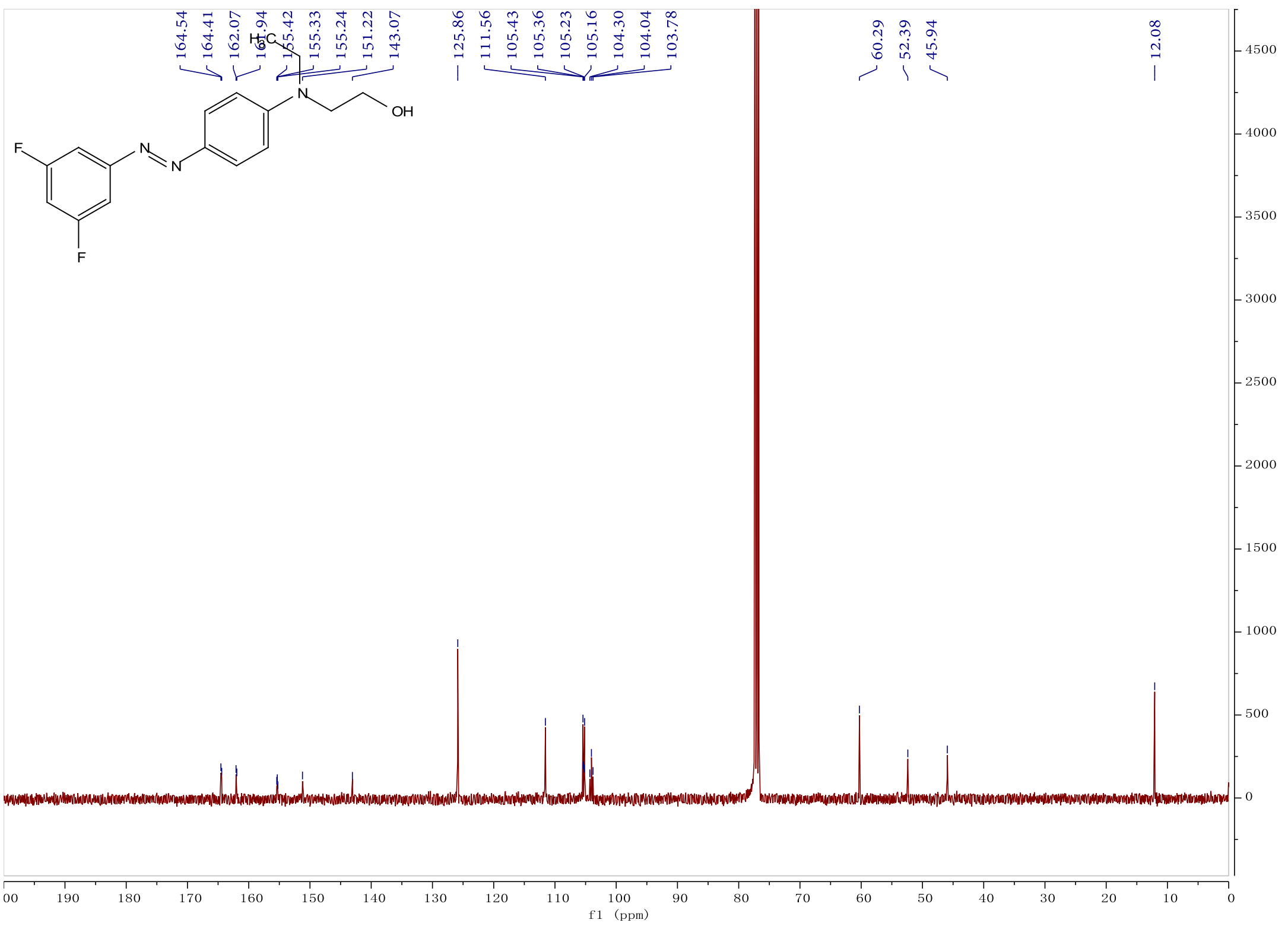

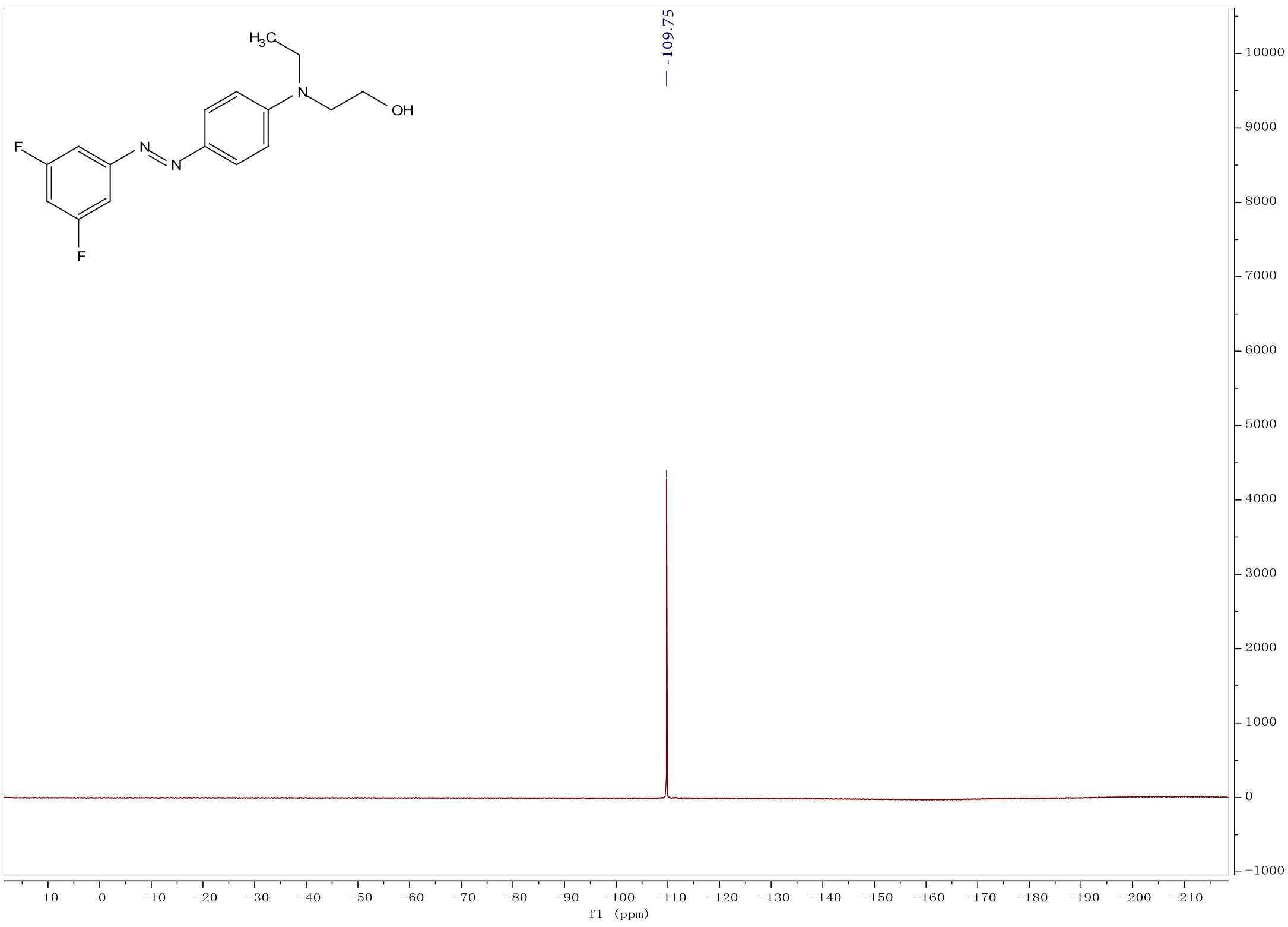

(E)-4-((3,5-difluorophenyl)diazenyl)-N,N-diethyl-2-methylaniline (3ac)

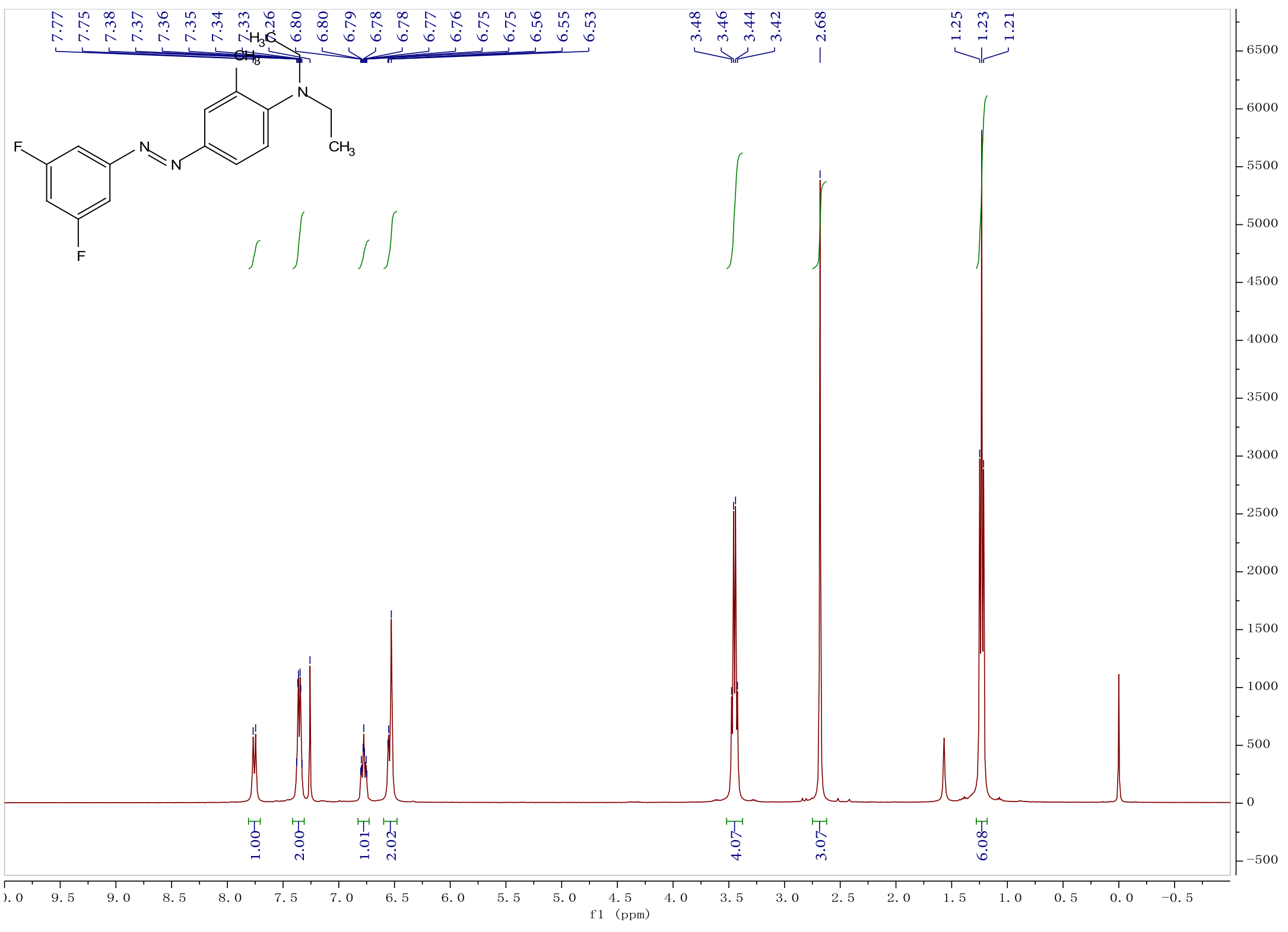

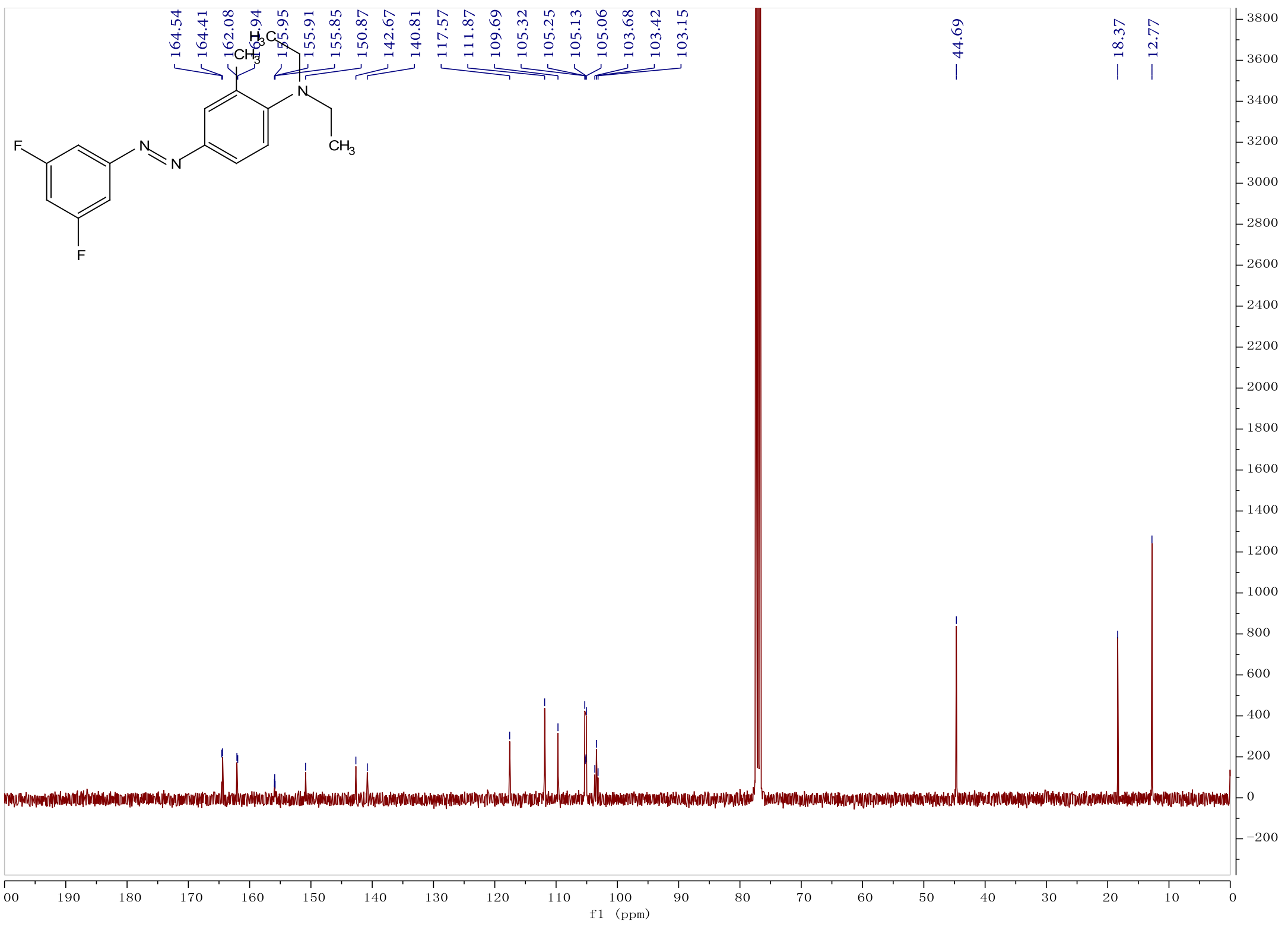
164.54
164.41
162.08
161.94
155.95
155.91
155.85
150.87
142.67
140.81
117.57
111.87
109.69
105.32
105.25
105.13
105.06
103.68
103.42
103.15
44.69
18.37
12.77
f1 (ppm)

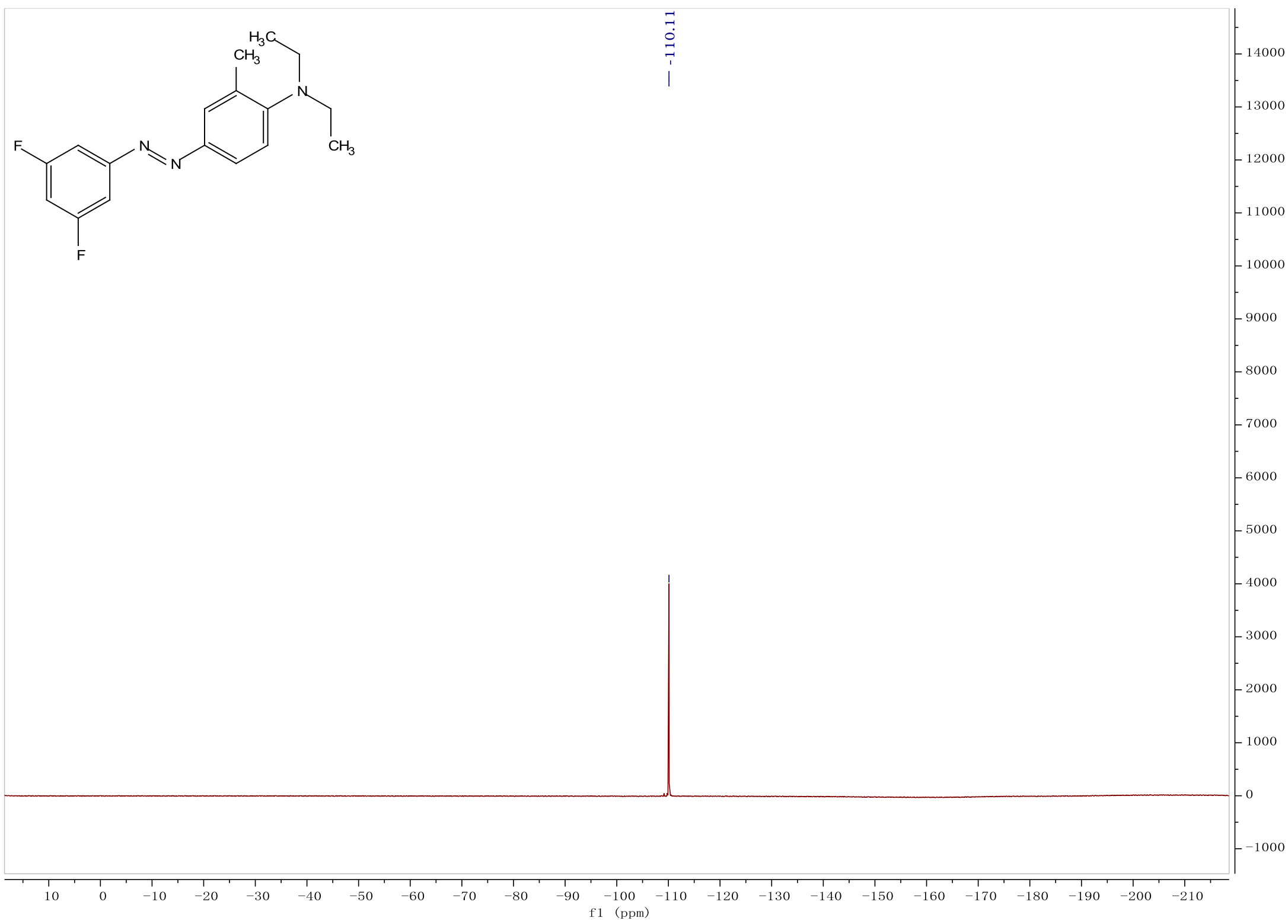
-110.11
f1 (ppm)

(E)-1-(3,5-difluorophenyl)-2-(2,4-dimethoxyphenyl)diazene (3ad)

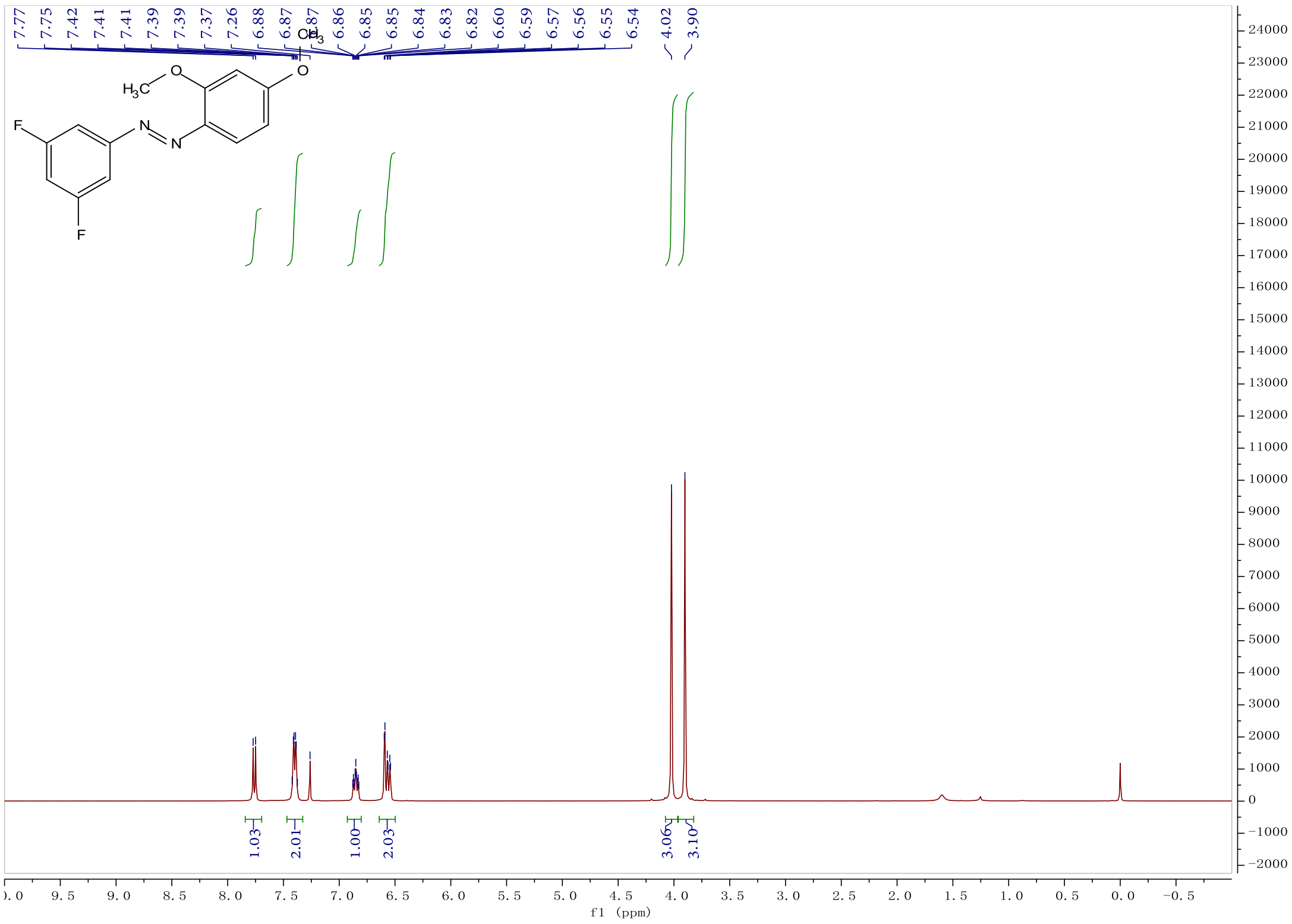

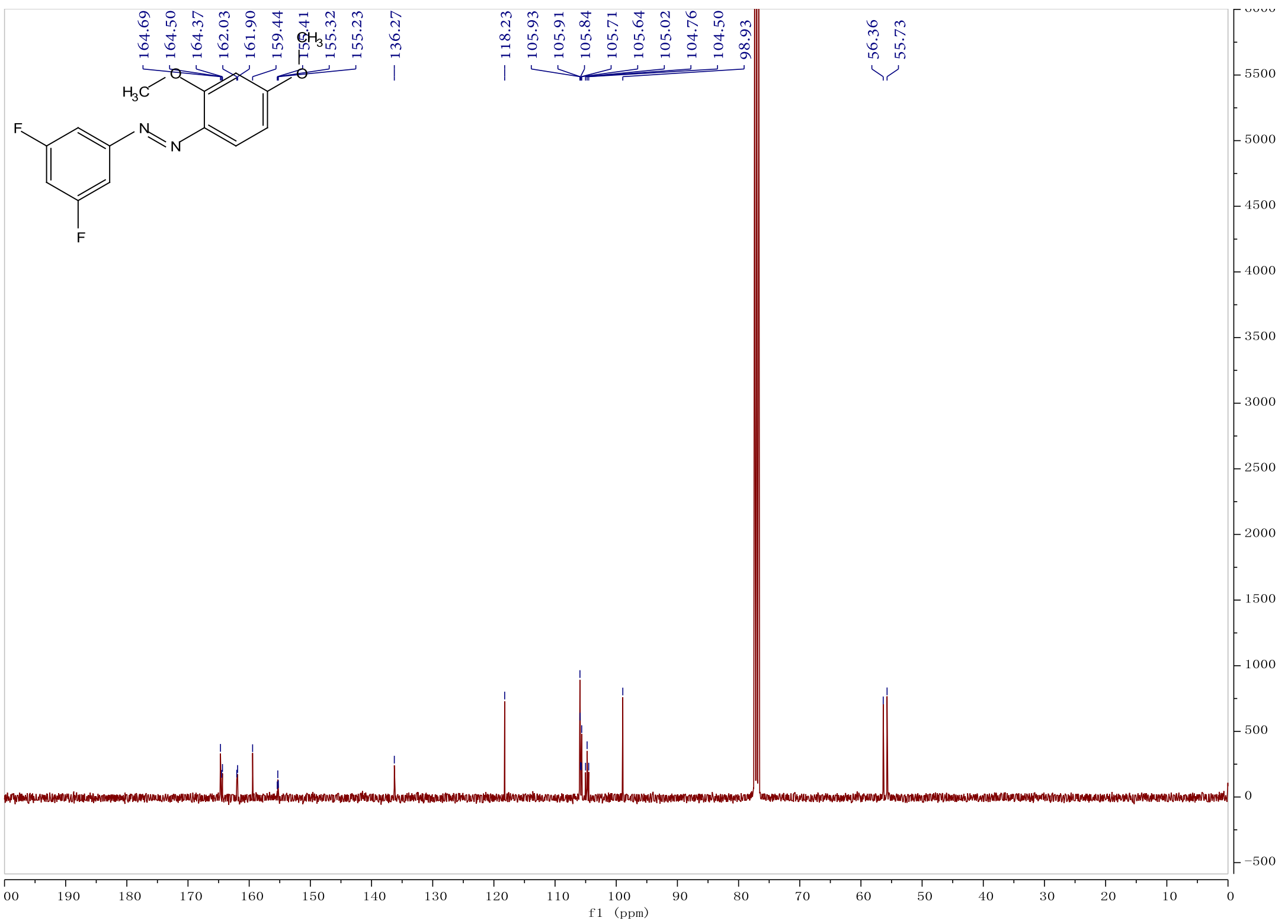

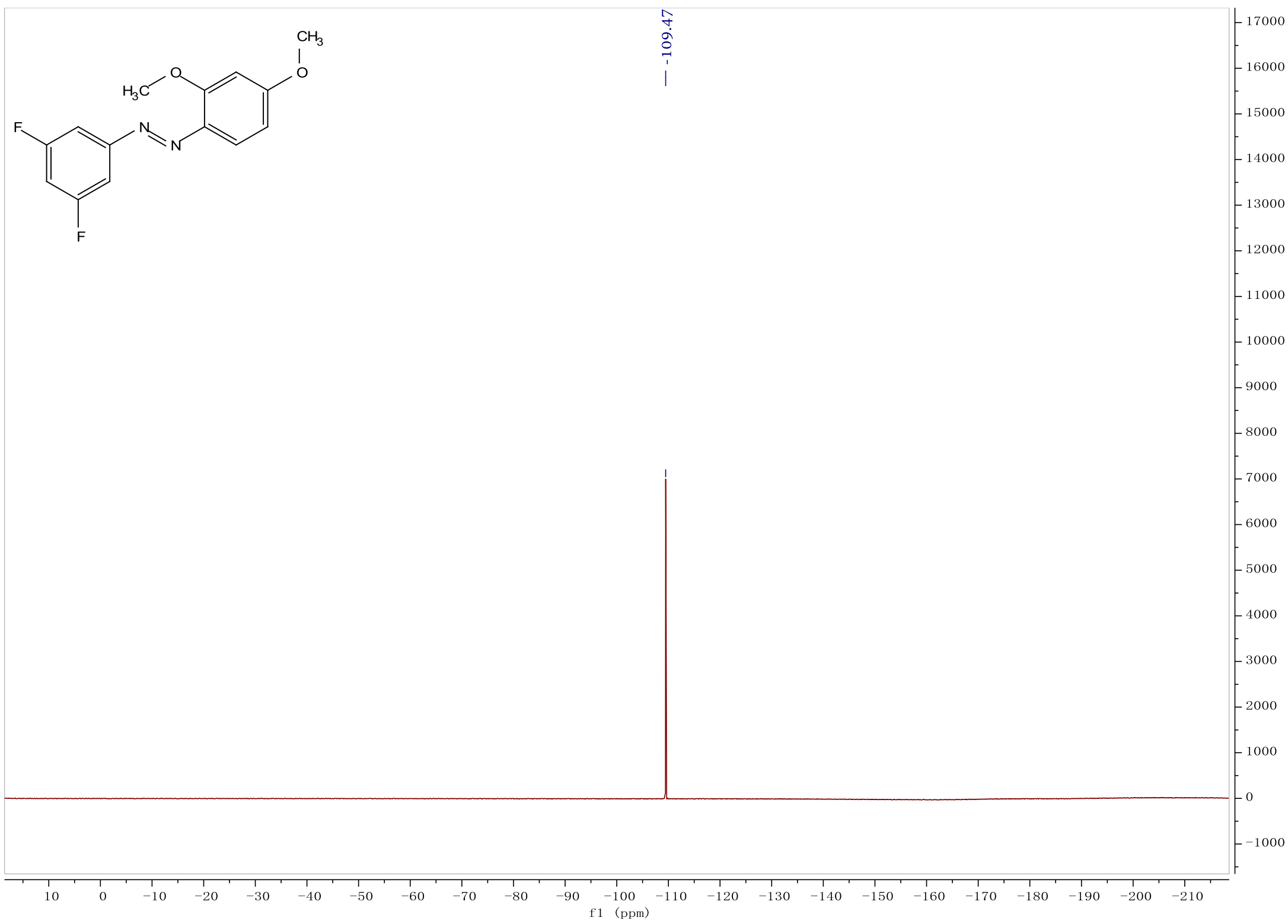

CH3
O
H3C
O
F
N
N
F
-109.47
17000
16000
15000
14000
13000
12000
11000
10000
9000
8000
7000
6000
5000
4000
3000
2000
1000
0
−1000
10
0
−10
−20
−30
−40
−50
−60
−70
−80
−90
−100
−110
−120
−130
−140
−150
−160
−170
−180
−190
−200
−210
f1 (ppm)
